# Manipulating Peierls-Nabarro stress to realize extraordinary thermoelectric performance in n-type PbTe

**DOI:** 10.1038/s41467-026-77575-0

**Published:** 2026-09-04

**Authors:** Chen Zhu, Hongwei Ming, Wenbin Gong, Guoxian Zhang, Feng Hu, Shouxi Jiang, Bin Hu, Xiaoying Qin, Fali Chong, Jian Zhang

**Affiliations:** 1 School of Physics and New Energy, Xuzhou University of Technology, Xuzhou, China; 2 Key Lab of Photovoltaic and Energy Conservation Materials, Institute of Solid State Physics, HFIPS, Chinese Academy of Sciences, Hefei, PR China; 3 University of Science and Technology of China, Hefei, China; 4 Fujian Science & Technology Innovation Laboratory for Optoelectronic Information of China, Fuzhou, China

**Keywords:** Thermoelectric devices and materials, Thermoelectrics

## Abstract

PbTe is recognized as one of the best intermediate-temperature thermoelectric materials; however, its commercial applications are largely restricted by the comparatively lower performance of its n-type counterpart. Here we show that Peierls-Nabarro stress is manipulated through solute-induced lattice softening, which generates high density helical dislocations, dislocation loops, and subgrain boundaries to block heat transport phonons. Concurrently, in-situ formation of Sb_6_O_13_ nanoinclusions resulting from a displacement reaction between Sb and TeO_2_ further suppresses thermal transport, leading to an ultra-low lattice thermal conductivity (*κ*_L_ ≈ 0.21 W m^−1^ K^−1^ at 773 K). Moreover, Sb doping can simultaneously enhance electronic density of states, leading to the improvement of power factor (16 μW cm^−1^ K^−2^ at 563 K). As a result, a remarkably high figure of merit (*ZT* ≈ 2.2) is obtained at 723 K in n-type PbTe-1.5 wt%TeO_2_-1.8 wt%Sb sample. Our findings provide an effective approach to synergistically improve the thermal and electrical properties in advanced thermoelectric materials.

## Abstract (web summary)

The authors manipulated Peierls-Nabarro stress via solute-induced lattice softening to generate dense dislocations in n-type PbTe. Combined with Sb_6_O_13_ nanoinclusions, this sharply reduces thermal conductivity, yielding a peak *ZT* of 2.2 at 723 K.

## Introduction

Thermoelectric materials, converting waste heat into electricity without greenhouse gas emissions, provide an effective method to resolve the problems of energy shortage and environmental pollution^1–7^. A superior thermoelectric material demands high figure of merit, *ZT*, expressed by *ZT* = *S*^2^*σT*/(*κ*_L_ + *κ*_e_), where *S*, *σ*, *T*, *κ*_L_ and *κ*_e_ denote thermopower, electrical conductivity, absolute temperature, lattice thermal conductivity and electronic thermal conductivity, respectively^8^. Due to the intercoupling of *κ*_e_, *σ*, and *S*, it is a major challenge to significantly elevate *ZT* through reducing the total thermal conductivity (*κ*_tot_ = *κ*_L_ + *κ*_e_) and improving power factor (*PF* = *S*^2^*σ*) at the same time.

Up to now, many promising thermoelectric materials have been discovered and developed, such as Bi_2_Te_3_^9,10^, SnSe^2,3,11^, PbTe^1,12–16^, GeTe^10,17^, etc. Therein, PbTe is recognized as one of the best intermediate-temperature thermoelectric materials due to its high crystal symmetry and distinctive electronic band structure. Various strategies have been implemented to improve the *ZT* of PbTe, including band convergence^12,18,19^, distortion of electronic density of states (DOS)^20^, energy-filtering effect^21^, nanostructures^22,23^ and all-scale hierarchitectures^13^. In particular, research shows that suppressing the relatively independent parameter *κ*_L_ by nanostructure or all-scale hierarchitectures is highly effective in boosting *ZT*. For instance, the pseudo-nanostructures, identified as clusters of cation vacancies at the nanoscale, are demonstrated to play an important role in scattering phonons^1^. The typical example is Pb_0.97_Na_0.03_Te-2%MgTe-0.75%GeTe composite with numerous pseudo-nanostructures that effectively scatter heat-carrying phonons and allow for the effective movement of charge-carrying holes because of the lattice coherence with the PbTe matrix. A peak *ZT* = 2.8 was obtained at 850 K, representing among the highest reported *ZT* values in p-type PbTe-based systems. Notably, the significant improvement is achieved in p-type PbTe. For n-type PbTe, despite remarkable progress, the *ZT*_max_ value reaches only ≈1.5-2.0^21,24–28^, which is well below the benchmark *ZT* of 2.2-2.8^1,13^ achieved in p-type counterparts. This performance gap between n-type and p-type PbTe significantly limits the overall conversion efficiency of PbTe-based thermoelectric modules, as high-performance devices require a close match between p- and n-type legs.

Hence, we focus on the further reduction of *κ*_L_ without degrading electrical transport properties in n-type PbTe. Dislocation engineering is pivotal for achieving broadband phonon scattering-a fundamental prerequisite for minimizing lattice thermal conductivity in high-performance thermoelectrics. Theoretically, the Peierls-Nabarro (P-N) stress^29–32^ represents the intrinsic lattice friction arising from the periodic potential energy landscape (the Peierls potential) that atoms within a dislocation core must traverse. Consequently, minimizing this stress effectively lowers the activation energy barrier for dislocation glide. Guided by this principle, we propose a strategy of solute-induced lattice softening to facilitate the generation and proliferation of dislocations^33^, thereby intensifying phonon scattering. We implemented this approach in n-type PbTe by systematically screening dopants (Sb, Bi, In, and Ga) to reduce the Peierls-Nabarro stress. To experimentally realize this, we designed a specific PbTe with 1.5 wt% TeO_2_ and *x* wt% Sb composition. The rationale behind this selection is that the fixed 1.5 wt% TeO_2_ triggers an in situ reaction with a portion of Sb to form endotaxial nanoinclusions for thermal stress, while the remaining variable Sb serves as the solute to soften the lattice. This dual-approach is projected to successfully minimize lattice thermal conductivity without compromising carrier mobility.

Indeed, our results demonstrate that substituting Sb for Pb yields an approximate 29% reduction in the Peierls-Nabarro stress for the (111) < 110> slip system (decreasing from 0.07 to 0.05 GPa) and high-density multiple types of dislocations (including helical dislocations, sub-grain boundaries and dislocation loops) were formed in the PbTe-1.5 wt%TeO_2_-*x*wt%Sb composites. Moreover, a large number of isostructural nanophases Sb_6_O_13_ (with a diameter of ≈20-80 nm) were created in PbTe matrix. The multiple dislocations and Sb_6_O_13_ nanoinclusions allow for remarkable mid- and low-frequency phonon scattering, which combined with high-frequency phonon scattering from point defects (e.g., ${{Sb}}_{{Pb}}^{{\prime} }$), leading to the sharp reduction of *κ*_L_ and *κ*_tot_ (*κ*_L_ ≈ 0.21 W m^−1^ K^−1^, *κ*_tot_ ≈ 0.42 W m^−1^ K^−1^). On the other hand, the substitution of Sb for Pb pushes the Fermi level *E*_F_ into conduction bands and introduces Sb 5p states that cause the increment of DOS at *E*_F_, improving the *S* and *PF* to ≈303 μV K^−1^ at 673 K and ≈16 μW cm^−1^ K^−2^ at 563 K, respectively. Consequently, a peak *ZT* value of 2.2 is obtained in PbTe-1.5 wt%TeO_2_-1.8 wt%Sb sample, which is about 10-fold larger than that of our pristine PbTe. The maximum *ZT* acquired here exceeds previously reported values for n-type PbTe thermoelectric materials^21,24–28^. Correspondingly, the three-dimensional finite-element simulated single-leg conversion efficiency reaches 14%.

## Results

### Peierls-Nabarro stress in doped PbTe

**Fig. 1:**
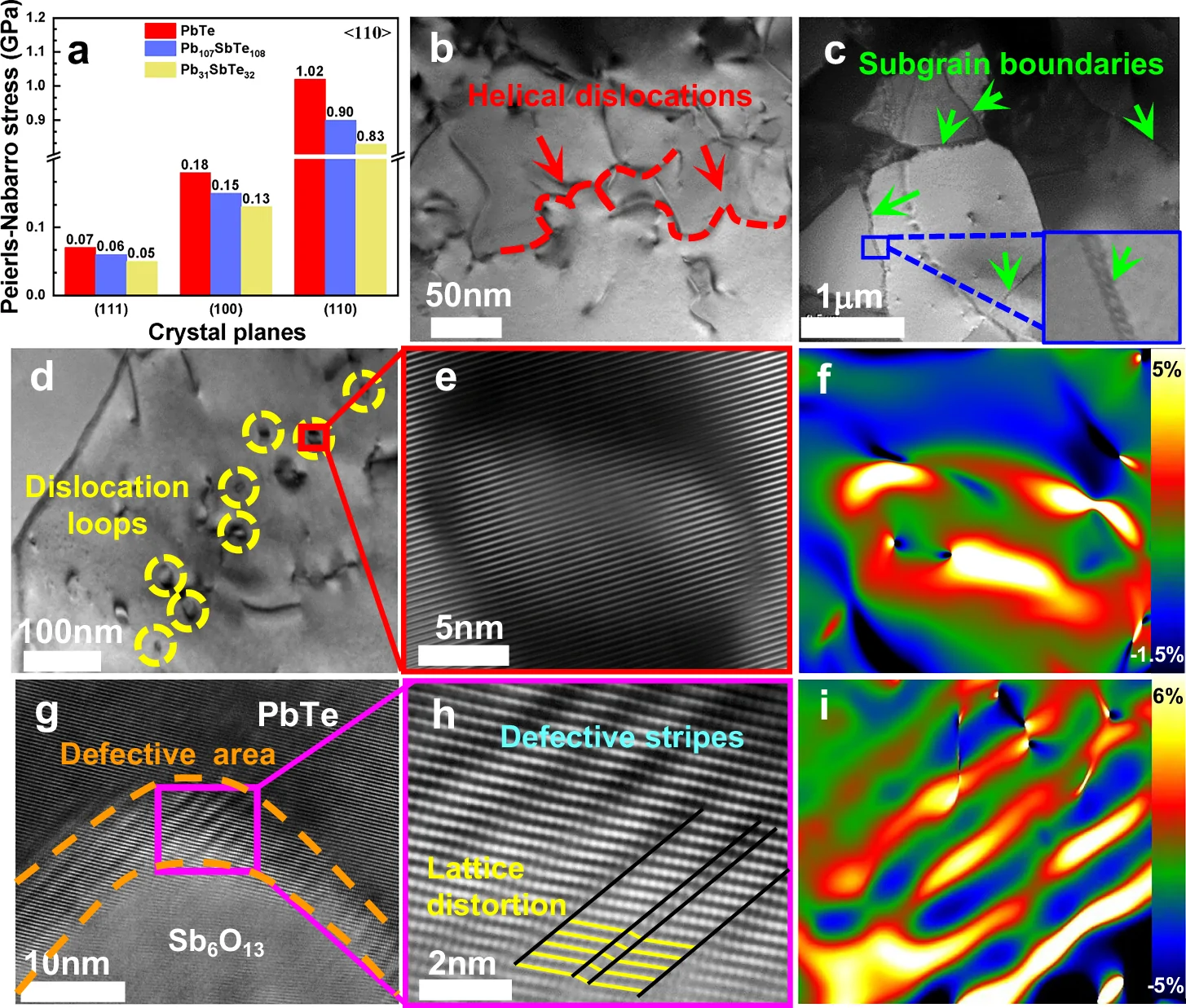
Peierls-Nabarro stress and microstructure characterization. **a** Low-index planes Peierls-Nabarro stress of PbTe and Sb-substituted PbTe samples. **b–d** Low-magnification TEM images of helical dislocations, subgrain boundaries and dislocation loops in PbTe-1.5 wt%TeO_2_-1.8 wt%Sb composite sample; **e** the HRTEM images of dislocation loop; **f** GPA strain analysis of (**e**); the HRTEM images of (**g**) defective area and **h** lattice distortion; **i** GPA strain analysis of (**h**).

In order to artificially promote the motion and multiplication of dislocations, we firstly investigated the effects of different elements substitutions on Peierls-Nabarro stress, which is the force needed to move a dislocation within a plane of atoms in the unit cell, of PbTe via first-principle calculation (the details are shown in section 2 of Supplementary Information). One can see from Fig. 1a that the substitution of Sb for Pb can effectively reduce the Peierls-Nabarro stress for (111) < 110> slip system from 0.07 to 0.05 GPa, which means that Sb-substituted PbTe samples are easily plastically deformed^33^ compared to pristine PbTe, and Sb substitution is beneficial for the dislocation motion and multiplication. Besides, Fig. 1a shows the low-index planes Peierls-Nabarro stress of PbTe and Sb-substituted PbTe samples. The Peierls-Nabarro stress for (111) close-packed atomic plane is lower than that for (100) and (110) crystal planes, which is consistent with the established fact that in FCC crystals, slip occurs between close-packed (111) atomic planes. The Peierls-Nabarro stress of In/Ga/Bi/Sb substituted PbTe is summarized in Supplementary Fig. 2.

### Phase and microstructure characterization

According to theoretical results mentioned above, we prepared PbTe-1.5 wt%TeO_2_-*x*wt%Sb samples. The electronegativity of Te is 2.1, which is larger than 2.05 of Sb and 1.8 of Pb. Thus, the extra TeO_2_ may react with Pb or Sb, leading to the in-situ formation of lead oxide or the oxide of antimony nanoprecipitates. The X-ray diffraction patterns of PbTe-1.5 wt%TeO_2_-*x*wt%Sb samples are displayed in Supplementary Fig. 3a. All major diffraction peaks can be indexed to the cubic structure of PbTe (PDF No. 03-065-2953). Besides, some weak impurity peaks are observed in the composite samples, which can be indexed to Sb_6_O_13_ phase (PDF No. 01-071-1091). The presence of Sb_6_O_13_ proves the occurrence of displacement reaction:

1 $$13{{\rm{TeO}}}_{2}+12{\rm{Sb}}\to 2{{\rm{Sb}}}_{6}{{\rm{O}}}_{13}+13{\rm{Te}}.$$

The XRD Rietveld refinements results in Supplementary Figs. 3c, d and Supplementary Table 2 show that with *x* increasing from 0 to 2.16, the mass fraction (*f*) of second phase Sb_6_O_13_ rises from 0 to 1.3 wt%. Besides, Supplementary Fig. 3b shows that the lattice parameter *a* drops monotonically from 6.457 to 6.439 Å, as *x* rises from 0 to 2.16, indicating continuous lattice shrinkage, which can be attributed to the substitution of Sb^3+^ (*r*_i_ = 0.92 Å) for Pb^2+^ (*r*_i_ = 1.20 Å)^34^. To further evaluate the chemical homogeneity of these samples, the energy-dispersive X-ray spectroscopy (EDS) mapping was performed as shown in Supplementary Fig. 4. The results suggest that Pb, Te, and Sb are macroscopically distributed in a homogeneous manner throughout the matrix. However, this apparent uniformity is primarily a reflection of the resolution limitations of SEM. While these observations confirm successful doping and elemental mixing at the micrometer scale, the identification of the Sb_6_O_13_ nanophases requires the application of higher resolution characterization techniques.

To confirm the effect of Sb substitution on the dislocations multiplication in PbTe materials, the transmission electron microscope (TEM) was performed to characterize the microstructures of PbTe-1.5 wt%TeO_2_-*x*wt%Sb composite samples. As we expected, Fig. 1 and Supplementary Fig. 5c, d show that PbTe-1.5 wt%TeO_2_-1.8 wt%Sb composite is embedded with a large number of defects, including helical dislocations, sub-grain boundaries, dislocation loops, lattice distortion, etc. The helical dislocations (Fig. 1b), which are closely related to interstitials or vacancies^35^ are usually observed in the rapidly quenched alloys^36^, and here we attribute the production of helical dislocations to the existence of intrinsic Pb vacancies^37^ and quenching technique. Figure 1c shows the low-resolution TEM images of sub-grain boundaries, which are characterized by sets of parallel and equidistant dislocations^38^. In addition, a high density of dislocation loops^39,40^ (the linear and enclosed dislocations), which originate from the lattice imperfections (e.g., helical dislocations/interstitial/vacancy), can be observed from Fig. 1d. The enlarged lattice image of red-square marked region in Fig. 1d shows the detailed lattice structure of dislocation loops (Fig. 1e). To evaluate the spatially distributed strain fields of dislocation loops, Geometric phase analysis (GPA^41^) of Fig. 1e was carried out. One can see from Fig. 1f that the loop strain lines are captured and demonstrate the intense strains along with dislocation loops. Although SEM-EDS suggests apparent compositional uniformity at the macroscale, high-resolution TEM reveals a PbTe/Sb_6_O_13_ two-phase microstructure at the nanoscale. As shown in Fig. 1g, a defective area exists at the interface between the PbTe matrix and Sb_6_O_13_ nanophase. These interfaces are characterized by the presence of periodic defective stripes. The defective stripes include high-density lattice distortion, which stems from the lattice mismatch between Sb_6_O_13_ and PbTe matrix. The lattice distortion can be clearly seen in the enlarged lattice image of defective area shown in Fig. 1h and the corresponding strain map are displayed in Fig. 1i. The lattice distortion in the PbTe matrix and the corresponding strain map are provided in Supplementary Fig. 6. Besides, quantitative statistical analysis of multiple TEM micrographs via Ham’s linear-intercept method reveals a pronounced elevation of dislocation density (*N*_D_) with increasing Sb solute content. For PbTe-1.5 wt% TeO_2_-0.36 wt% Sb, *N*_D_ reaches 1.14 × 10^10^ cm^−2^; this value rises to 3.89 × 10^10^ cm^−2^ for PbTe-1.5 wt% TeO_2_-1.08 wt% Sb, and peaks at an ultrahigh magnitude of 6.99 × 10^10^ cm^−2^ in the optimal PbTe-1.5 wt% TeO_2_-1.8 wt% Sb composite (Supplementary Table 4). In PbTe-1.5 wt% TeO_2_-1.8 wt% Sb sample, the average spatial separation of entangled dislocation networks is reduced to ~37.8 nm, a length scale comparable to the intrinsic phonon scattering cross-section of individual dislocations (~28 nm). Such dense dislocation populations originate from Sb-induced lattice softening and suppressed P-N stress, which drastically lower the energetic barrier for dislocation multiplication during melt quenching and spark plasma sintering. The overlapping strain fields emanating from interconnected dislocation lines produce strong broadband scattering of mid- and low-frequency heat-carrying phonons, and play a significant role in suppressing lattice thermal conductivity.

**Fig. 2:**
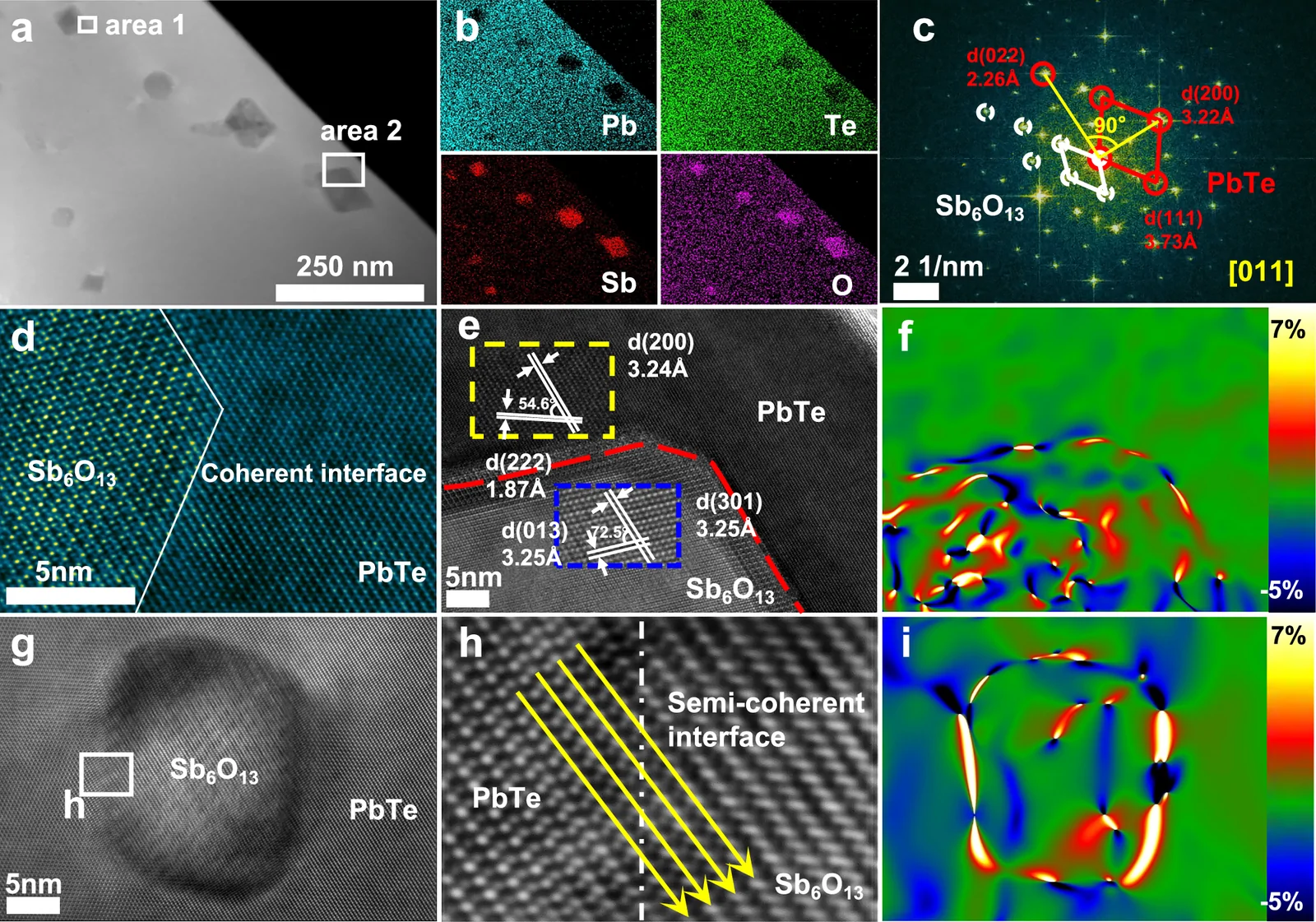
Microstructures of nanoprecipitate in PbTe-1.5 wt%TeO_2_-1.8 wt%Sb sample. **a** Low-magnification STEM ADF image of Sb_6_O_13_ nanoprecipitates; **b** EDS elemental mapping of Pb, Te, Sb, and O; **c** FFT image of the selected area 1 in (**a**); **d** atomic-resolution interface image of the selected area 1 in (**a**); **e** high-magnification ABF image of the selected area 2 in (**a**); **f** GPA analysis of (**e**); **g** high-magnification ABF image of Sb_6_O_13_ nanoprecipitate; **h** atomic-resolution interface image of the selected area in (**g**) as marked with a white rectangle, where the semi-coherent interface relations are shown; **i** GPA analysis of (**g**).

In addition to the multiple dislocations, well-faceted nanophases with diameter of ≈40-80 nm were observed in PbTe-1.5 wt%TeO_2_-1.8 wt%Sb composite sample as shown in Fig. 2 and Supplementary Figs. 7a, b. Figure 2a is a scanning transmission electron microscopy (STEM) annular dark-field (ADF) image of the composite sample. The EDS elemental mapping of Fig. 2a shows that all of these nanophases are rich in Sb and O elements (Fig. 2b). The fast fourier transform (FFT) results of selected area 1 in Fig. 2a confirms the nanophases possess Sb_6_O_13_ cubic phase with space group Fd-3m, being consistent with XRD results (Supplementary Fig. 3a). Moreover, the FFT indexing along the [011] zone axis (Fig. 2c) reveals that the Sb_6_O_13_ nanophases exhibit a specific orientation relationship with the host lattice. Notably, the diffraction spots of the secondary phase are coaxially aligned with the {111} reflections of the PbTe matrix (*d* = 3.73 Å), confirming that the nanoprecipitates preferentially form a Widmanstätten-like pattern along the {111} habit planes. This oriented growth is driven by the minimization of interfacial energy and explains the directional alignment observed in high-resolution images. Besides, the spots belong to PbTe are overlapped with Sb_6_O_13_, indicating that PbTe/Sb_6_O_13_ form coherent/semi-coherent interfaces in the certain direction of the crystal structure. Our atomic-resolution interface images (Fig. 2d, g, h) also prove the point. One can clearly see the coherent/semi-coherent interfaces and uniform lattice orientation between PbTe and Sb_6_O_13_ phases (Fig. 2d, h). This special interface relationship is beneficial to electronic transport. Figure 2e displays the high-resolution annular bright-field (ABF) images of area 2 in Fig. 2a. The measured lattice spacing and angle are close to the theoretical value in PDF database (PDF No. 03-065-2953 for PbTe and PDF No. 01-071-1091 for Sb_6_O_13_), manifesting the existence of Sb_6_O_13_ nanophases in PbTe matrix. Lattice mismatch between the nanophases Sb_6_O_13_ and matrix PbTe produced dense strains, as shown in Fig. 2f. Additionally, some smaller nanophases (diameter ≈20-30 nm) were observed, as presented in Fig. 2g and Supplementary Figs. 7a, b. The GPA of Fig. 2g was performed to show the strain fields, as displayed in Fig. 2i. Strong strain around the interfaces is induced due to these small-sized and high dense nanophases, which combined with multiple dislocation mentioned above, would definitely lead to the sharp reduction of *κ*_L._

### Thermal transport properties

**Fig. 3:**
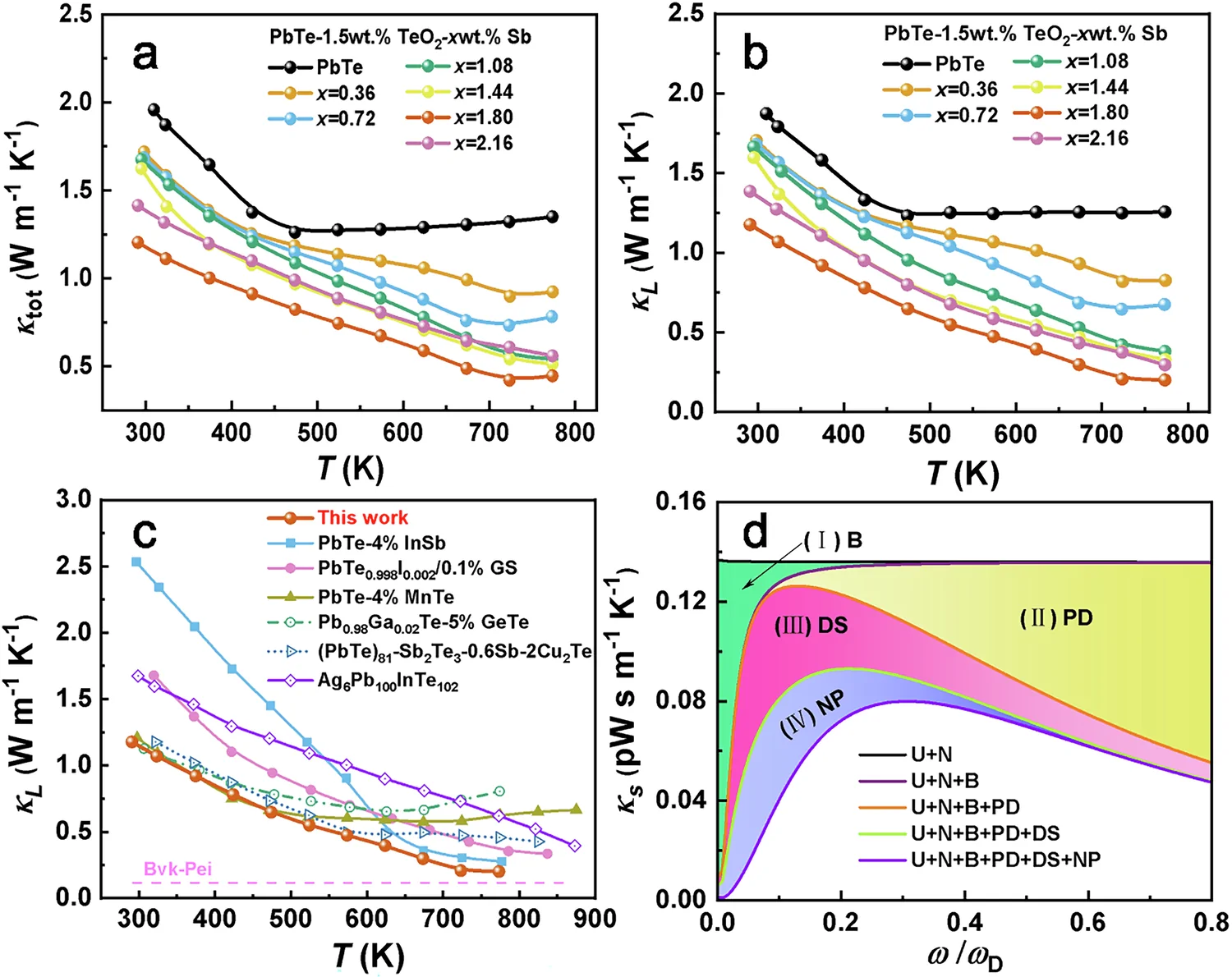
Thermal conductivity of PbTe-1.5 wt%TeO_2_-*x*wt%Sb samples. Temperature-dependent total thermal conductivity (*κ*_tot_) (**a**) and lattice thermal conductivity (*κ*_L_) (**b**) for PbTe-1.5 wt%TeO_2_-*x*wt%Sb samples. **c** shows the *κ*_L_ for PbTe-1.5 wt%TeO_2_-1.8 wt%Sb sample compared with the typical reported results^21,24–28^; the dashed line in (**c**) is the theoretical *κ*_Lmin_ calculated by Bvk-Pei model. **d** Calculated spectral lattice thermal conductivity *κ*_s_ (at 300 K) for PbTe-1.5 wt%TeO_2_-1.8 wt%Sb sample with various phonon scattering.

The temperature dependence of total thermal conductivity *κ*_tot_ is shown in Fig. 3a. It can be seen that the thermal transport of PbTe-1.5 wt%TeO_2_-*x*wt%Sb (*x* > 0) is significantly suppressed compared with pristine PbTe in the whole investigated temperature range. The *κ*_tot_ of PbTe-1.5 wt%TeO_2_-1.8 wt%Sb is 0.45 W m^−1^ K^−1^ at 773 K, which is only one-third of the value for pristine PbTe (1.35 W m^−1^ K^−1^). The minimum *κ*_tot_ ≈ 0.42 achieved at 723 K in PbTe-1.5 wt%TeO_2_-1.8 wt%Sb is lower than previously reported values for PbTe-based systems^21,24–28^, as shown in Supplementary Fig. 9. The *κ*_L_ is obtained by subtracting *κ*_e_ from *κ*_tot_ and shown in Fig. 3b. One can see that the *κ*_L_ of pristine PbTe first reduces, as *T* < 473 K, then rises a little bit as *T* increases further due to the bipolar diffusion effect, similar to the results reported in other PbTe-based systems^24,42,43^. The *κ*_L_ of PbTe-1.5 wt%TeO_2_-*x*wt%Sb continuously decreases with increasing temperature, but a slight rise occurs at *T* ˃ 723 K for *x* = 0.36 and 0.72 samples. The variation of the upturn of *κ*_L_ indicates the suppression of bipolar diffusion^44^, which can be attributed to the broaden effective band gap due to the increased electron concentration^24^. Moreover, PbTe-1.5 wt%TeO_2_-*x*wt%Sb samples have smaller *κ*_L_ as compared to PbTe especially at high temperatures (for instance, the *κ*_L_ reduced from 1.26 W m^−1^ K^−1^ in pristine PbTe to 0.21 W m^−1^ K^−1^ in PbTe-1.5 wt%TeO_2_-1.8 wt%Sb at 773 K), resulting from the broadband phonon scattering (discussed later) and suppression of bipolar diffusion. One can see from Fig. 3c that the *κ*_L_ for PbTe-1.5 wt%TeO_2_-1.8 wt%Sb sample is lower than that of the typical reported results in the whole investigated temperature range; besides, the *κ*_L_ obtained in this work is higher than theoretical minimum lattice thermal conductivity of PbTe calculated via Bvk-Pei model^45,46^.

To quantitatively analyze the impact of the microstructure regulation (i.e., multiple dislocations, and isostructural nanophases) on the sharply reduced *κ*_L_, we invoke the Debye-Callaway model defined by^47,48^:

2 $${{\rm{\kappa }}}_{{\rm{L}}}=\frac{4\pi {k}_{{\rm{B}}}^{4}{T}^{3}}{v{h}^{3}}{\int }_{0}^{{\theta }_{{\rm{D}}}/T}{\tau }_{{\rm{T}}}\frac{{z}^{4}{e}^{z}}{{[{e}^{z}-1]}^{2}}{dz}$$

in which *v*, *θ*_D_, *τ*_T_ and *z* denote average sound velocity, Debye temperature, total phonon relaxation time and reduced phonon frequency (*z* = *ħω*/*κ*_B_*T*), respectively. *τ*_T_ includes the contributions from Umklapp (*τ*_U_) and Normal (*τ*_N_) phonon-phonon, grain/phase boundaries (*τ*_B_), point defect (*τ*_PD_), dislocation strain fields and dislocation cores (*τ*_DS_) and nanophases (*τ*_NP_) scattering. *τ*_T_ can be expressed via the following formula^48^:

3 $${{\tau }}_{{\rm{T}}}^{-1}={{\tau }}_{{\rm{U}}}^{-1}+{{\tau }}_{{\rm{N}}}^{-1}+{{\tau }}_{{\rm{B}}}^{-1}+{{\tau }}_{{\rm{PD}}}^{-1}+{{\tau }}_{{\rm{DS}}}^{-1}+{{\tau }}_{{\rm{NP}}}^{-1}.$$

To validate the physical reliability of the dislocation-induced relaxation rates used in the Debye-Callaway modeling, the quantified individual dislocation characteristics are summarized in Supplementary Table 4. One can see from Supplementary Fig. 10 that the calculated results (black curve) fit well with our experimental results (dots). Besides, as *x* rises from 0.36 to 0.72, 1.08, 1.44 and 1.80, *κ*_L_ at 723 K reduces from 0.82 to 0.65, 0.38 and 0.21 W m^−1^ K^−1^ and then increases to 0.37 W m^−1^ K^−1^ as *x* increases further to 2.16. For the *x* = 2.16 sample, the upturn in *κ*_L_ originates from the formation of larger Sb_6_O_13_ nanophases (≈120-200 nm, Supplementary Fig. 7c-e), which reduces the density of effective scattering centers. Specifically, the number density *N*_P_ decreases from 6.5 × 10^21 ^m^−3^ for *x* = 1.80 to 1.5 × 10^21 ^m^-3^ for *x* = 2.16 due to particle coarsening (Supplementary Table 3). This reduction in *N*_P_ diminishes the efficiency of phonon scattering, leading to the increased *κ*_L_. Besides, the spectral lattice thermal conductivity *κ*_s_ was calculated for PbTe-1.5 wt%TeO_2_-1.8 wt%Sb sample to elucidate the contribution of different scattering mechanisms by using the model shown below:

4 $${{\rm{\kappa }}}_{{\rm{s}}}=\frac{4\pi {k}_{{\rm{B}}}^{4}{T}^{3}}{v{h}^{3}}{\tau }_{{\rm{T}}}\frac{{z}^{4}{e}^{z}}{{[{e}^{z}-1]}^{2}}.$$

One can see from Fig. 3d that the grain/phase boundaries (2D defects) and point defects (0D defects) scatter low- and high-frequency phonons, respectively; whereas the multiple dislocations (1D defects) and the nanophases (3D defects) strongly scatter low- and mid-frequency phonons. Ultimately, broadband phonons were intensively scattered by multidimensional defects, resulting in the dramatic reduction of *κ*_L_.

### Electrical transport properties

**Fig. 4:**
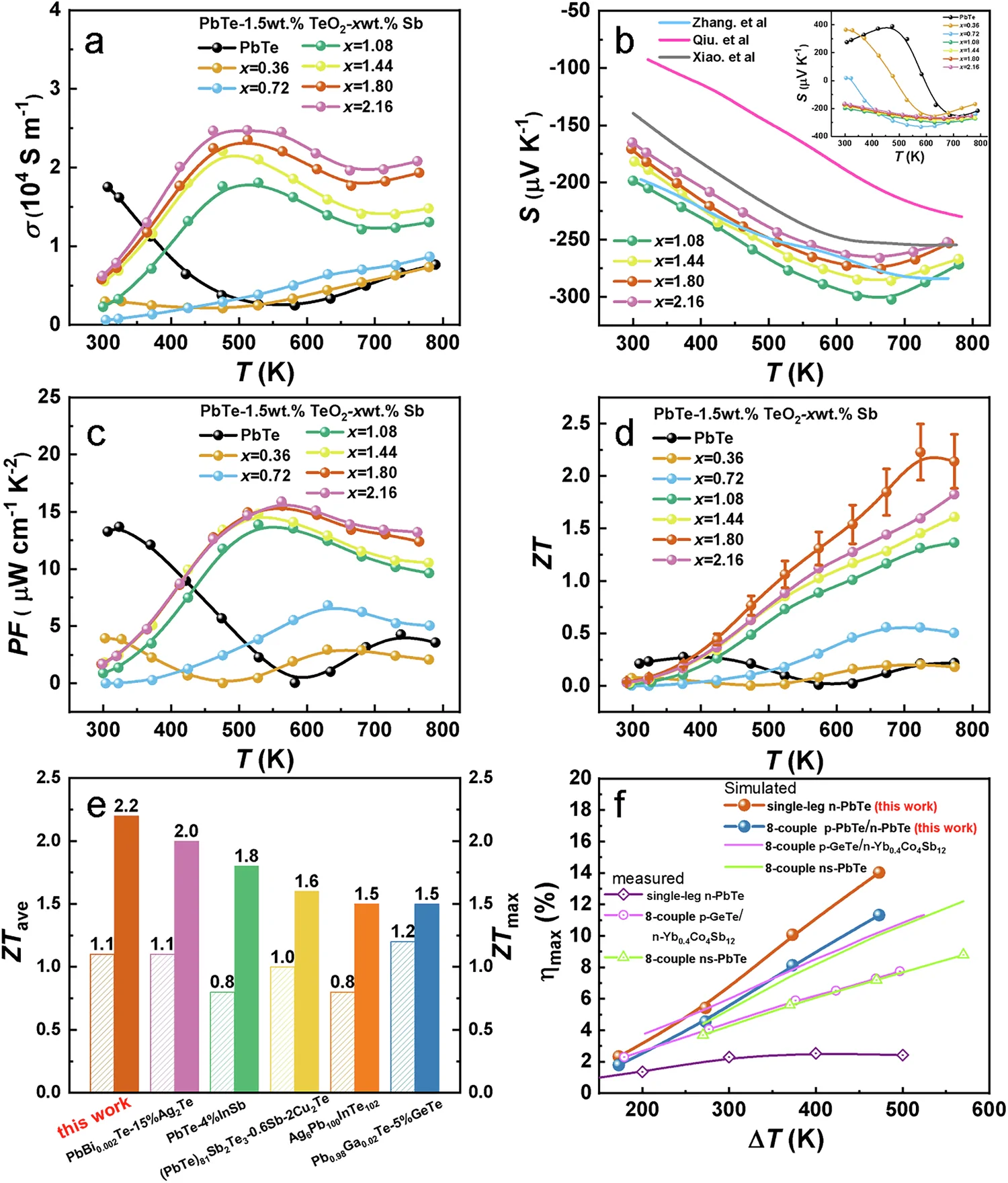
Thermoelectric properties of PbTe-1.5 wt%TeO_2_-*x*wt%Sb samples. Temperature dependence of (**a**) electrical conductivity (*σ*), (**b**) thermopower (*S*), (**c**) *PF* and (**d**) *ZT*. The solid lines in (**b**) are the *S* of state-of-the-art n-type PbTe composites in literatures^21,25,26^. **e** shows average *ZT* and maximum *ZT* values for PbTe-1.5 wt%TeO_2_-1.8 wt%Sb sample compared with reported data^21,24,27,50,52^; **f** The three-dimensional finite-element simulated highest *η* compared with reported values for various types of thermoelectric devices of 8-couple PbTe/Yb_0.4_Co_4_Sb_12_ (simulated and measured)^53^, 8-couple nanostructured PbTe (simulated and measured)^54^ and single-leg n-type PbTe module^55^.

The measured electrical properties of all samples are shown in Fig. 4. One can see from Fig. 4a, b that the electricity conductivity *σ* and thermopower *S* of pristine PbTe exhibit an apparent transition (from p- to n-type^49^) at ≈573 K. As the content of Sb increases to 0.36 and 0.72, the transition temperature decreases to 473 K and 300 K, respectively, revealing that more electrons are activated by Sb doping. For the samples with *x* ≥ 1.08, the transition of carrier-type disappears completely and full electron-type dominates the electronic transport. The temperature-dependent carrier concentration is provided in Supplementary Fig. 11a. The *σ* of PbTe-1.5 wt%TeO_2_-*x*wt%Sb composites (1.08 ≤ *x* ≤ 2.16) initially rises with increasing temperature, then reduces at 500-673 K, and then increases at high temperature (*T* ≥ 673 K). The anomalous behavior of *σ* is quite different from the traditional PbTe-based systems reported before^4,26,50,51^, indicating the different charge carriers scattering mechanisms in different temperature range (as shown Supplementary Fig. 11b), which was discussed in section 7 of Supplementary Information.

The *S* of PbTe-1.5 wt%TeO_2_-*x*wt%Sb composites (1.08 ≤ *x* ≤ 2.16) is boosted over the whole working temperature range and the maximum *S* reaches 303 μV K^−1^ at 673 K in PbTe-1.5 wt%TeO_2_-1.08 wt%Sb sample, which evidently outperforms the reported values in other n-type PbTe systems^21,25,26^, as shown in Fig. 4b. Additionally, the absolute value of thermopower |*S*| monotonously decreases from 271.6 to 252.1 μV K^−1^ (at 773 K) with Sb content *x* increasing from 1.08 to 2.16. We attribute this phenomenon to the increased electron concentration (as shown in Supplementary Fig. 11a) due to the substitution of Sb^3+^ for Pb^2+^ which provides extra electrons, as *S* is inversely proportional to electron concentration based on below formulas^20^:

5 $$S=\frac{{\pi }^{2}{k}_{B}^{2}T}{3e}{\left(\frac{\partial {\mathrm{ln}}(\sigma \left(E\right))}{\partial E}\right)}_{E={E}_{{\rm{F}}}},$$

6 $$S=\frac{{\pi }^{2}{k}_{B}^{2}T}{3e}{\left(\frac{1}{n}\frac{\partial n\left(E\right)}{\partial E}+\frac{1}{\mu }\frac{\partial \mu \left(E\right)}{\partial E}\right)}_{E={E}_{{\rm{F}}}}.$$

Based on the Mahan-Sofo theory, formula (6) can be simplified for degenerate semiconductors as^20^

7 $$S=\frac{8{\pi }^{2}{{\kappa }_{B}}^{2}T}{3e{h}^{2}}{m}_{d}^{*}{(\frac{\pi }{3n})}^{2/3}$$

where *h*, *κ*_B_, and ${m}_{d}^{*}$ denote Planck constant, Boltzmann constant and density of states effective mass, respectively. According to Eq. (7), the enhanced ${m}_{d}^{*}$ would lead to the enhancement of *S*. In order to quantitatively analyze the behavior of thermopower, a single parabolic band (SPB) model was applied to calculate the density of states effective mass ${m}_{d}^{*}$ for PbTe-1.5 wt%TeO_2_-*x*wt%Sb samples with *x* ≥ 1.08. Supplementary Fig. 12 shows that the ${m}_{d}^{*}$ increases from ≈ 0.58 *m*_*e*_ for PbTe-1.5 wt%TeO_2_-*x*wt%Sb with *x* = 1.08 to 0.72 *m*_*e*_ for the sample with *x* = 2.16. Besides, our DFT calculation (Supplementary Fig. 13) indicates that the increased ${m}_{d}^{*}$ is originated from the enhanced DOS around conduction band minimum contributed by the Sb (5p) states.

### Thermoelectric performance

Combining with the optimized *σ* and significantly enhanced *S*, the *PF* is boosted and shows an obvious increase with increasing *x* (1.08 ≤ *x ≤* 2.16). One can see from Fig. 4c that the maximum power factor *PF*_max_ reaches ≈16 μW cm^−1^ K^−2^ at 563 K for PbTe-1.5 wt%TeO_2_-2.16 wt%Sb sample. The well optimized electrical and thermal properties lead to a significantly enhanced figure of merit *ZT* for PbTe-1.5 wt%TeO_2_-*x*wt%Sb, as shown in Fig. 4d. Especially, the *ZT* of PbTe-1.5 wt%TeO_2_-1.8 wt%Sb composite reaches 2.2 at 723 K, which exceeds previously reported values for n-type PbTe-based systems^21,24,27,50,52^ (Fig. 4e). This peak Z*T* value demonstrates good repeatability and thermal stability, as shown in Supplementary Fig. 14 and Supplementary Fig. 15. Additionally, the value of average *ZT* was calculated via ${{ZT}}_{{\rm{ave}}}=\frac{{\int }_{{T}_{{\rm{c}}}}^{{T}_{{\rm{h}}}}{ZT}{d}_{T}}{{T}_{{\rm{h}}}-{T}_{{\rm{c}}}}$, where *T*_h_ and *T*_c_ are the hot- and cold-side temperatures, respectively. One can see from Fig. 4e that our work presents high *ZT*_ave_ (≈1.1), which is comparable with the reported *ZT*_ave_ for advanced n-type PbTe-based systems^21,24,27,50,52^. Moreover, the electrical output and conversion efficiency *η* of single-leg PbTe module and 8-couple PbTe-based module were investigated using 3-dimensional finite-element simulation in COMSOL Multiphysics (see section 10 of Supplementary Information). One can see from Fig. 4f that the *η*_max_ of single-leg reaches 14% and the *η*_max_ of 8-couple PbTe-based thermoelectric device is 11.3%, which represent the theoretical projection of material potential.

## Discussion

In summary, we have demonstrated that manipulating the Peierls-Nabarro stress via solute-induced lattice softening offers a robust pathway to overcome the performance bottleneck in n-type PbTe. This strategy fosters a complex hierarchy of defects including helical dislocations, dislocation loops, and subgrain boundaries, which in concert with in-situ formed Sb_6_O_13_ nanoinclusions and ${{Sb}}_{{Pb}}^{{\prime} }$ point defects, realizes broadband phonon scattering and ultra-low lattice thermal conductivity. Coupled with the simultaneous enhancement of the electronic density of states, this synergistic decoupling of transport parameters yields a peak *ZT* ≈ 2.2 at 723 K. Our findings provide a generalizable blueprint for leveraging stress-targeted defect engineering to optimize the performance of state-of-the-art thermoelectric materials.

## Methods

### Sample synthesis

High purity Pb shots (5 N), Sb powder (5 N), Te chunks (4 N), TeO_2_ powder (4 N) were weighed in the stoichiometric ratio, and loaded into evacuated quartz tubes, then melted at 1323 K for 24 h and quenched in cool water. The resulting ingots were crushed and hand ground for 30 min. The obtained powders were sintered and densified by Spark Plasma Sintering (SPS) at 823 K for 5 min under a pressure of 50 Mpa in a vacuum atmosphere.

### Structural characterization

The phase structure was analyzed via X-ray diffraction patterns (Philips X’Pert PRO diffractometer, with Cu Kα radiation, *λ* = 1.5406 Å). The field-emission scanning electron microscopy (FE-SEM; SU8020) and transmission electron microscopy (TEM; JEOLF2010) were performed to characterize the microstructures.

### Property measurements

The thermopower (*S*) and electrical conductivity (*σ*) were simultaneously measured via the commercial four-probe equipment (ULVAC-RIKO, ZEM-3). The thermal conductivity (*κ*) was obtained by the relationship of *κ* = *DdC*_p_, where the thermal diffusivity coefficient (*D*) was measured by a laser flash method (NETZSCH, LFA-457) and the density *d* was measured via the Archimedes method. The heat capacity *C*_p_ was estimated by Dulong-Petit limit. In order to check whether Dulong-Petit Law is suitable for this system, the *C*_p_ of 2 typical samples (PbTe and PbTe-1.5 wt%TeO_2_-1.8 wt%Sb samples) were measured by a differential scanning calorimeter (NETZSCH, DSC-404 F3), as shown in Supplementary Fig. 1. One can see from Supplementary Fig. 1 that the Dulong-Petit approximation is consistent with our measured *C*_p_ in the whole investigated temperature range. Thus, Dulong-Petit Law is used for the determination of heat capacity for all the samples thereafter. The electronic thermal conductivity *κ*_e_ is estimated on the basis of Wiedemann-Franz law: *κ*_e_ = *LσT* (here *L* is the Lorentz number obtained from the formula *L* = (1.5+exp[−|*S*|/116]) × 10^−8^ V^2^ K^−2^ for all specimens). The Hall coefficient (*R*_H_) was obtained using a commercial Hall Effect measurement system (Lake Shore 8400 Series).

### Computational details

All the density-functional theory (DFT) calculations were carried out with the Vienna ab initio simulation package (VASP) using the Perdew-Burke-Ernzerhof (PBE) generalized gradient approximation (GGA) and projector augmented wave (PAW) pseudopotentials. Before the calculation of the elastic constant matrix and electronic structures, all the ionic positions were relaxed to equilibrium until the calculated Hellmann-Feynman forces on each atom were less than 0.02 eV*/*Å. The plane-wave cutoff energy of 500 eV was set up. Monkhorst-Pack k-point mesh of and an energy conversion criterion of 1 × 10^−5 ^eV per atom were used in the calculations. The spin-orbit coupling effect was not taken into account, nevertheless, it is still reliable to describe the morphology of the energy band.

## Supplementary information

Supplementary Information

Transparent Peer Review file

## Source data

Source Data

## Data Availability

The data generated in this study are provided in the Supplementary Information/Source Data file. Source data are provided with this paper.
