# Supplementary material for: Manipulating Peierls-Nabarro stress to realize extraordinary thermoelectric performance in n-type PbTe
Source: Nat Commun. 2026 Sep 4;17:10536. doi: 10.1038/s41467-026-77575-0 (PMC13642244; doi:10.1038/s41467-026-77575-0)
Supplement: Supplementary file 1 [file 41467_2026_77575_MOESM1_ESM.pdf]

Supplementary Information

Manipulating Peierls-Nabarro stress to realize extraordinary thermoelectric performance in n-type PbTe

Chen Zhu[1,2,3]†, Hongwei Ming[2,3,4]†, Wenbin Gong[1], Guoxian Zhang[1], Feng Hu[1], Shouxi Jiang[1], Bin Hu[1], Xiaoying Qin[2,3*], Fali Chong[1*], Jian Zhang[2,3*]

[1] School of Physics and New Energy, Xuzhou University of Technology, Xuzhou 221018, China.

[2] Key Lab of Photovoltaic and Energy Conservation Materials, Institute of Solid State Physics, HFIPS, Chinese Academy of Sciences, 230031, Hefei, PR China

[3] University of Science and Technology of China, Hefei 230026, China

[4] Fujian Science & Technology Innovation Laboratory for Optoelectronic Information of China, Fuzhou, Fujian 350108, China

† These authors contributed equally

* Corresponding authors: xyqin@issp.ac.cn, flchong2008@163.com , zhangjian@issp.ac.cn

1. Experimental specific heat values

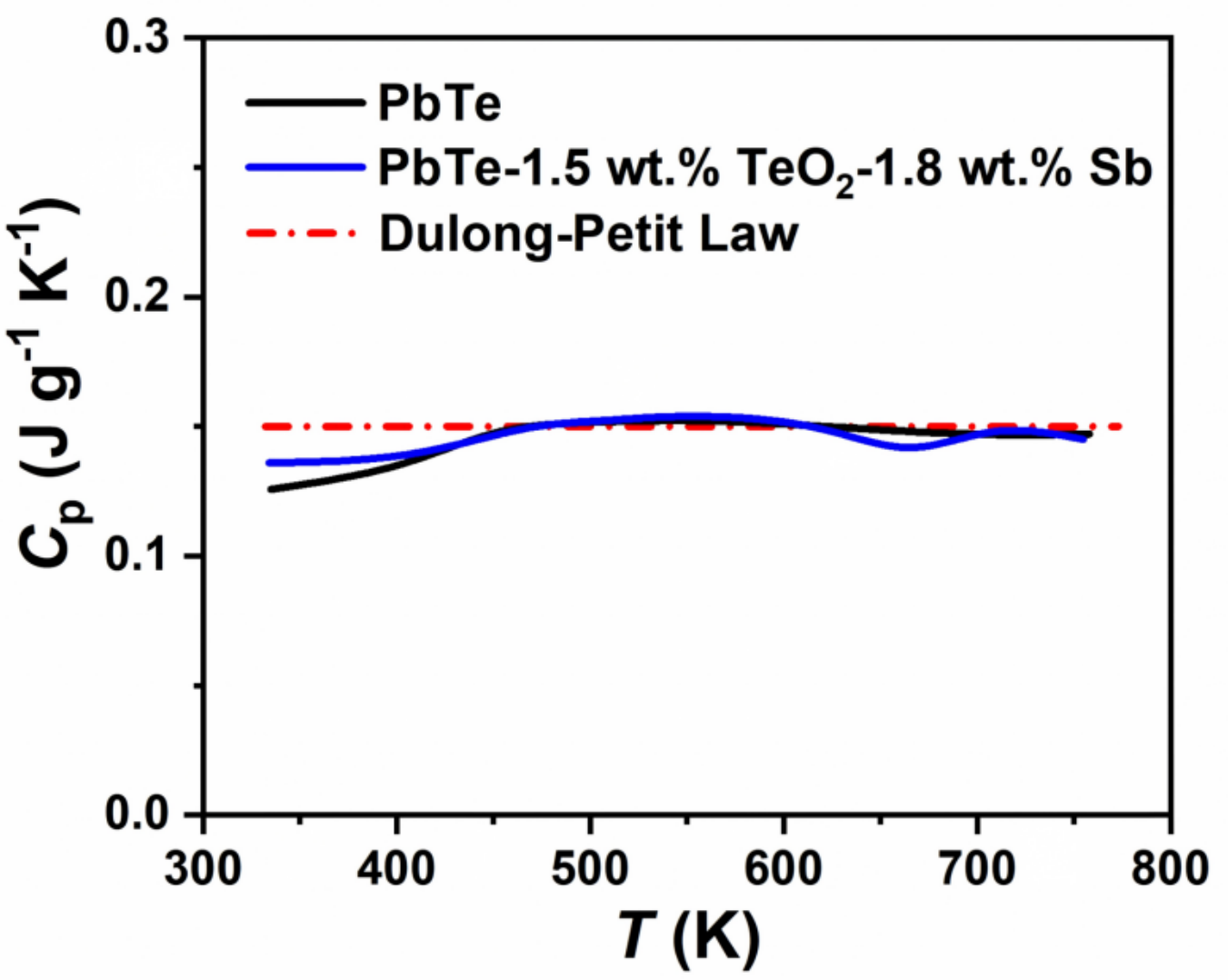

Supplementary Fig. 1 | Temperature dependence of specific heat (C_p) for PbTe and PbTe-1.5wt.%TeO_2-1.8wt.%Sb samples.

2. Theoretical calculations of Peierls-Nabarro stress

The Peierls-Nabarro stress can be calculated by the formula, as given below[1]:

$$\sigma_{PN} = \frac{2G}{1-\nu} e^{-2\pi W/b} \tag{1}$$

where $W=a/(1-\nu)$ represents the width of dislocation, G shear modulus, ν Poisson's ratio, b interatomic spacing, a interplanar crystal spacing. G and ν can be calculated *via* the following formula[2]:

$$G_R = 5(C_{11} - C_{12})C_{44}/[4C_{44} + 3(C_{11} - C_{12})] \tag{2}$$

$$G_v = (C_{11} - C_{12} + 3C_{44})/5 \tag{3}$$

$$G = (G_R + G_v)/2 \tag{4}$$

$$B = B_v = B_R = (C_{11} + 2C_{12})/3 \tag{5}$$

$$E = 9GB/(G+3B) \tag{6}$$

$$\nu = (E-2G)/2G \tag{7}$$

Where the subscript V denotes the Voigt bound, R denotes the Reuss bound, B is bulk modulus, E is Young's modulus, C_{ij} are elastic constants that can be calculated upon first-principles density functional theory method. The parameters are listed in **Supplementary Table 1**.

Supplementary Table 1. Elastic constants C_{ij}, shear modulus G (GPa), bulk modulus B (GPa), Young's modulus E (GPa), Poisson's ratio ν and Peierls-Nabarro stress σ_{PN} for PbTe doped with Sb, Bi, In, Ga elements.

Nominal composition	C_{11}	C_{12}	C_{44}	G	B	E	ν	σ_{PN}
**PbTe**	130.32	5.70	13.84	26.66	47.24	67.32	0.26	0.07
**$Pb_{0.96875}Sb_{0.03125}Te$**	125.87	7.27	11.41	23.71	46.80	60.85	0.28	0.05
**$Pb_{0.96875}Bi_{0.03125}Te$**	126.42	7.55	10.47	22.84	47.17	58.99	0.29	0.05
**$Pb_{0.96875}Ga_{0.03125}Te$**	124.01	6.72	13.57	25.59	45.81	64.73	0.26	0.06
**$Pb_{0.96875}In_{0.03125}Te$**	125.50	6.81	13.83	26.00	46.37	65.71	0.26	0.07

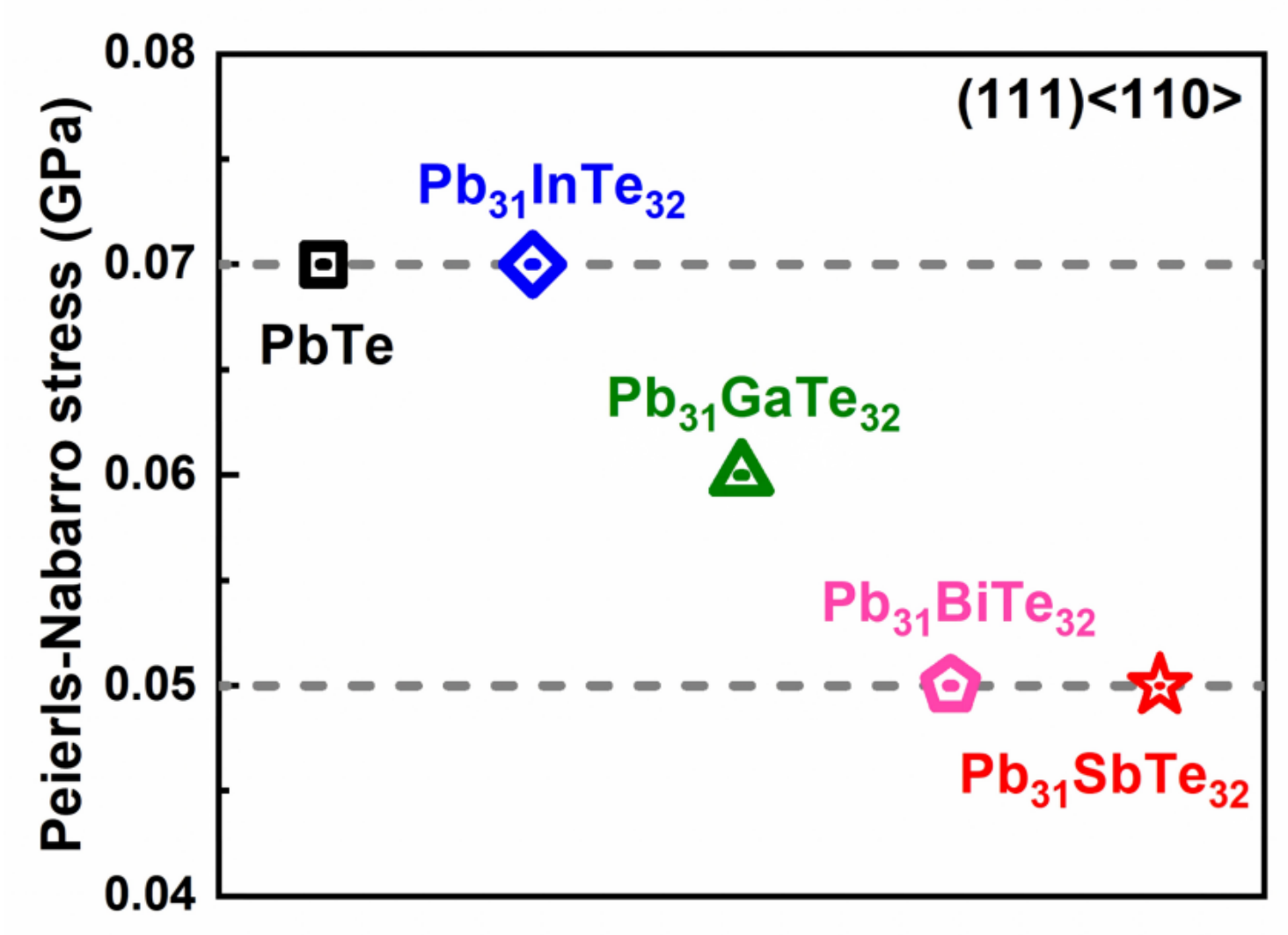

Supplementary Fig. 2 | Calculated Peierls-Nabarro stress for (111) <110> slip system of PbTe and In/Ga/Bi/Sb substituted PbTe samples.

3. **Phase characterization**

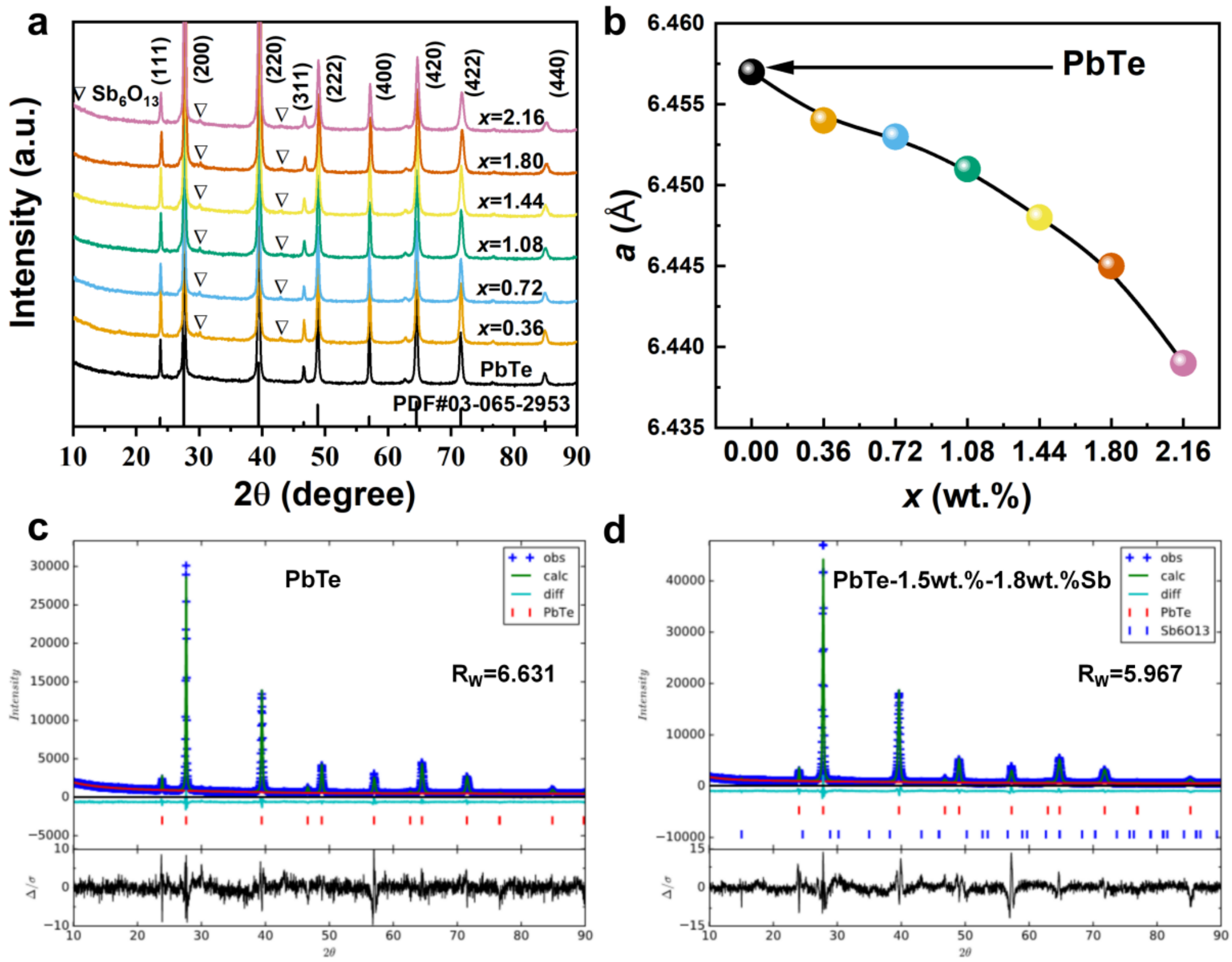

Supplementary Fig. 3 | (a) XRD patterns and **(b)** lattice parameter for PbTe-1.5wt.%TeO_2-*x*wt.%Sb samples. The Rietveld refinement results of **(c)** PbTe and **(d)** PbTe-1.5wt.%TeO_2-1.8wt.%Sb samples.

Supplementary Table 2. Relative Density (d_r), the mass fraction (f) of impurity phase Sb_6O_{13} obtained from Rietveld refinement analysis and the factor of GSAS Rietveld Refinement (R_w) for PbTe-1.5wt.%TeO_2-$x$wt.%Sb samples.

Nominal composition	d_r (%)	f (wt.%)	R_w(%)
**PbTe**	98.70	0	6.631
**PbTe-1.5wt.%TeO_2-0.36wt.%Sb**	96.87	0.2	6.178
**PbTe-1.5wt.%TeO_2-0.72wt.%Sb**	96.07	0.5	5.790
**PbTe-1.5wt.%TeO_2-1.08wt.%Sb**	95.78	0.7	5.666
**PbTe-1.5wt.%TeO_2-1.44wt.%Sb**	96.51	0.9	6.138
**PbTe-1.5wt.%TeO_2-1.80wt.%Sb**	95.95	1.2	5.967
**PbTe-1.5wt.%TeO_2-2.16wt.%Sb**	98.64	1.3	6.015

4. **Microstructure characterization**

To confirm that the PbTe matrix of PbTe-1.5wt.%TeO_2-1.8wt.%Sb composite system was doped by Sb, we performed EDS mapping of this composite sample. One can see from **Supplementary Figs. 4d-g** that all elements (Pb, Te, Sb) are homogeneously distributed in the detection area, implying that Pb was partially substituted by Sb during the vacuum melting process.

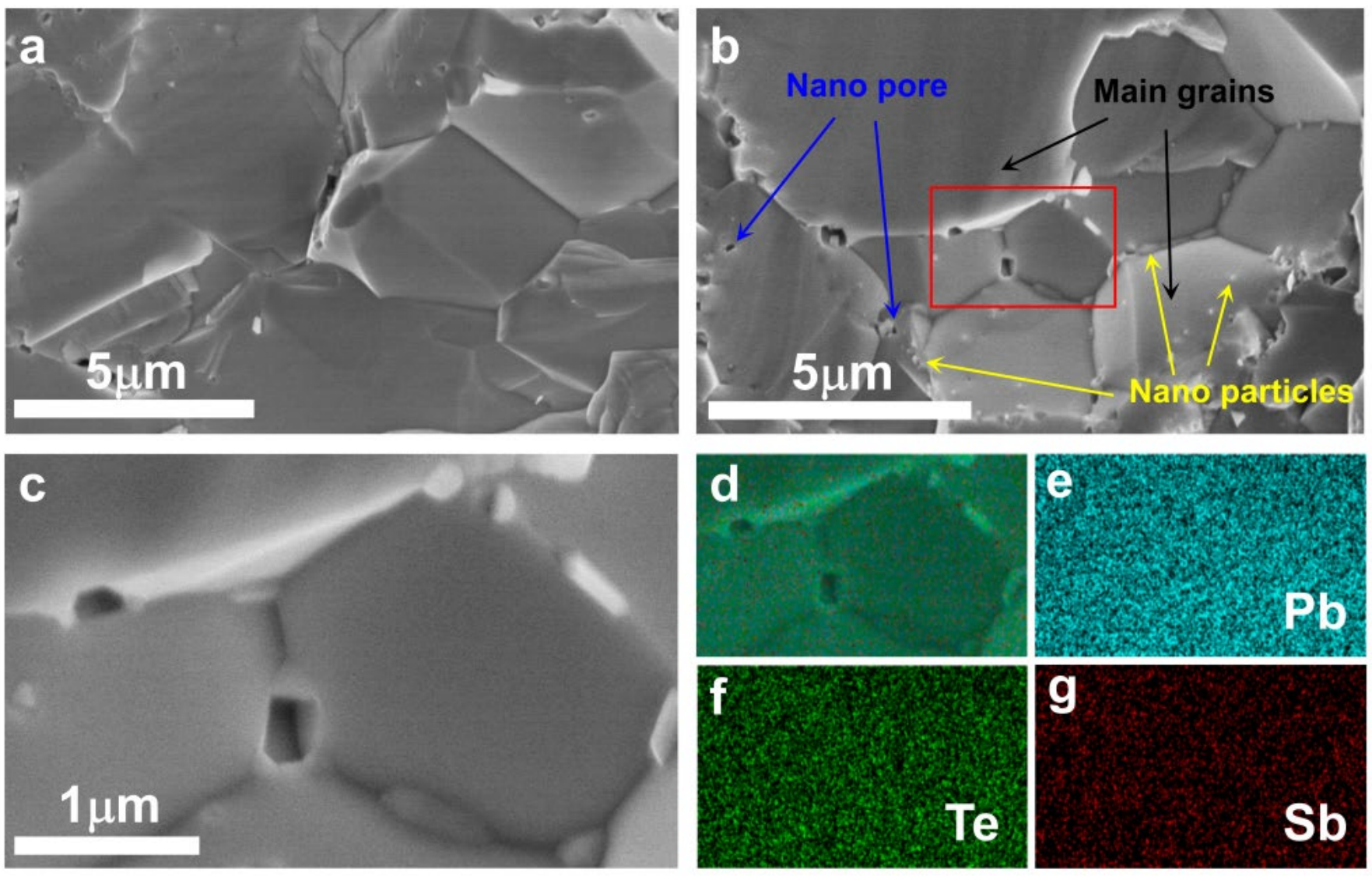

Supplementary Fig. 4 | FE-SEM images of the fracture surface of **(a)** PbTe and **(b)** PbTe-1.5wt.%TeO_2-1.8wt.%Sb sample; **(c)** magnified image of selected rectangle area in **(b)**; **(d-g)** EDS analysis of the elements Pb, Te and Sb.

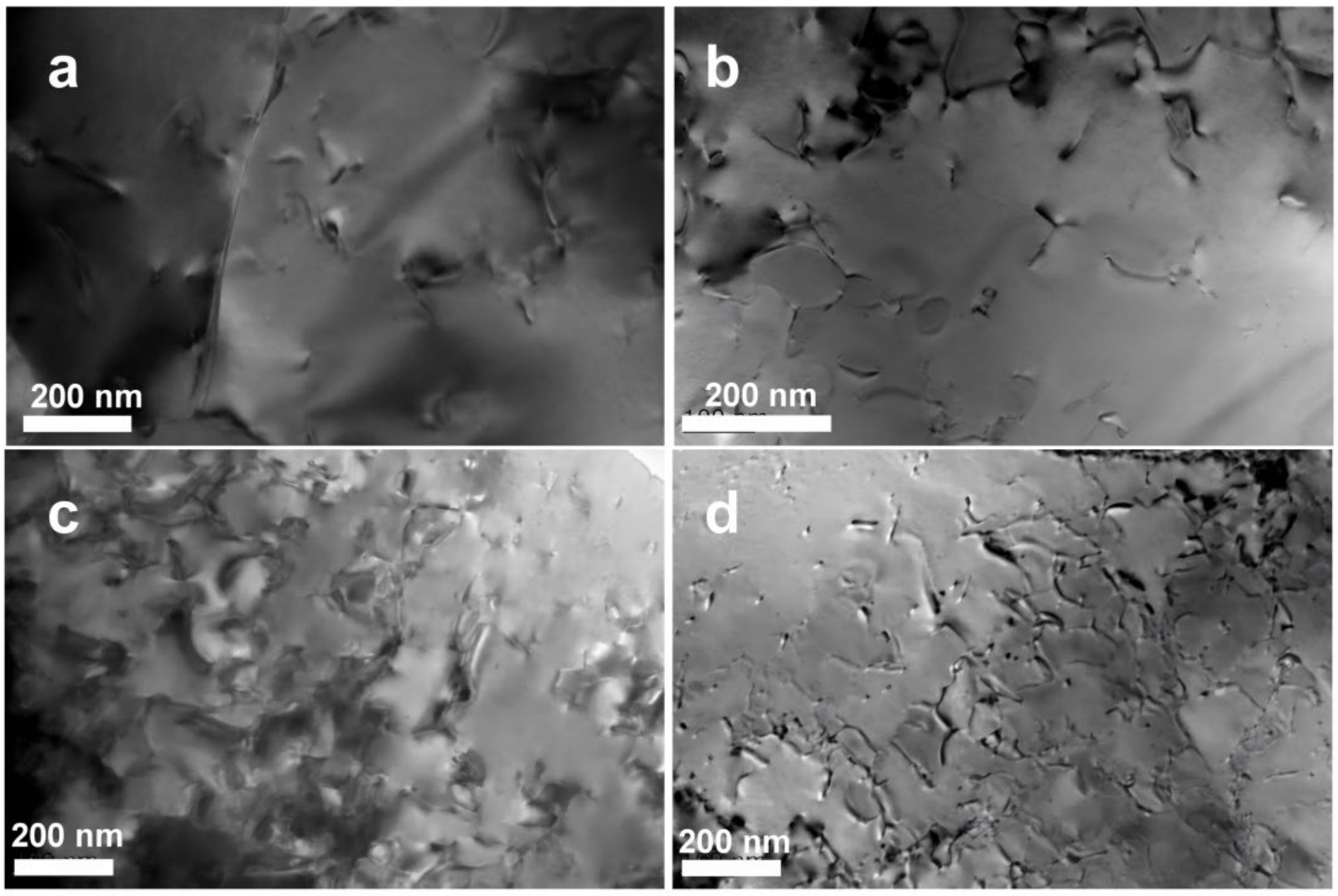

Supplementary Fig. 5 | STEM micrographs of dislocations in (a) PbTe-1.5wt.%TeO_2-0.36wt.%Sb, (b) PbTe-1.5wt.%TeO_2-1.08wt.%Sb and (c-d) PbTe-1.5wt.%TeO_2-1.80wt.%Sb samples.

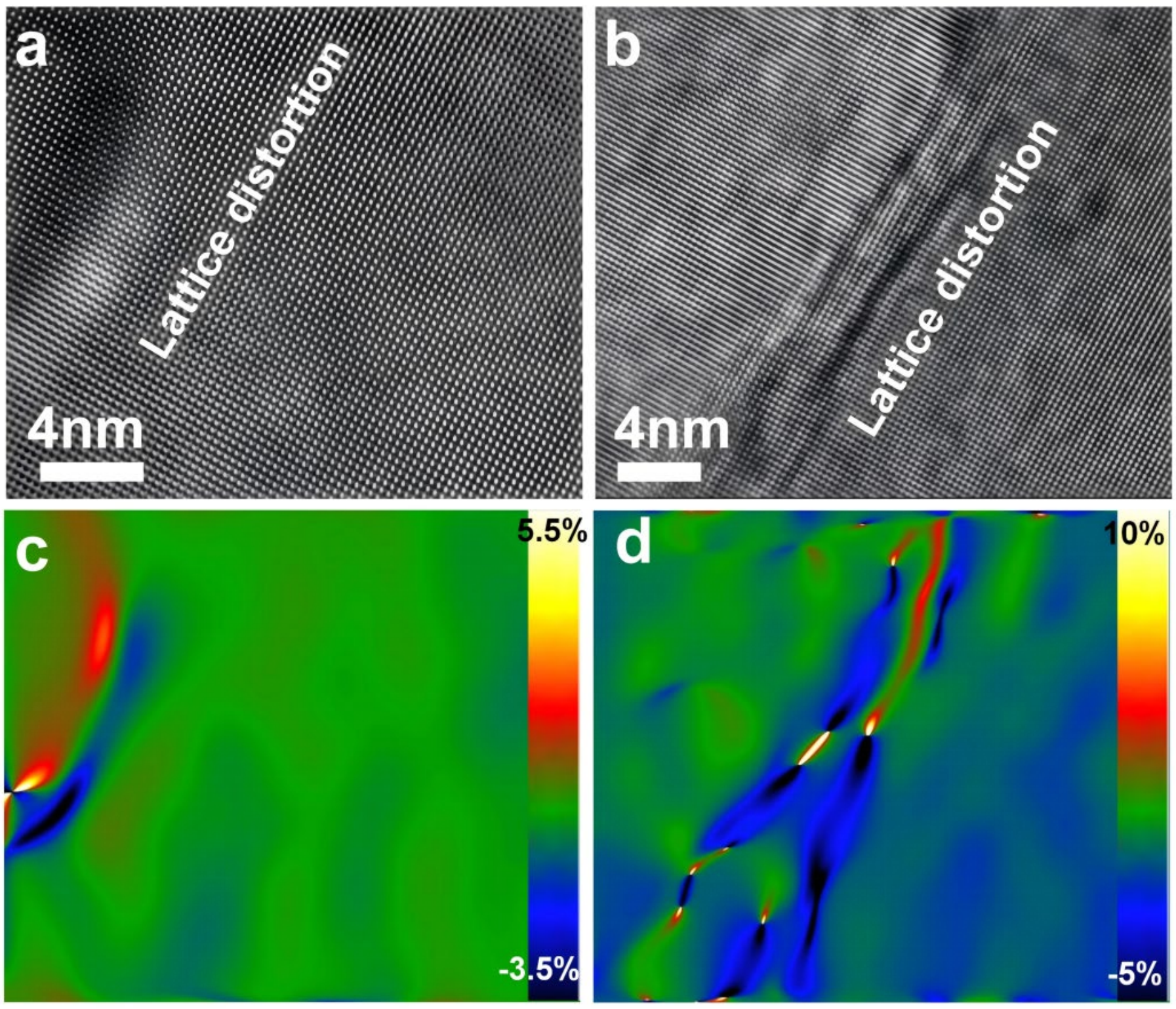

Supplementary Fig. 6 | (a-b) HRTEM micrographs of lattice distortion in PbTe-1.5wt.%TeO_2-1.8wt.%Sb sample; **(c-d)** the GPA analysis of **(a-b)**. Note that all TEM observations and GPA mapping shown here were performed specifically on the PbTe matrix.

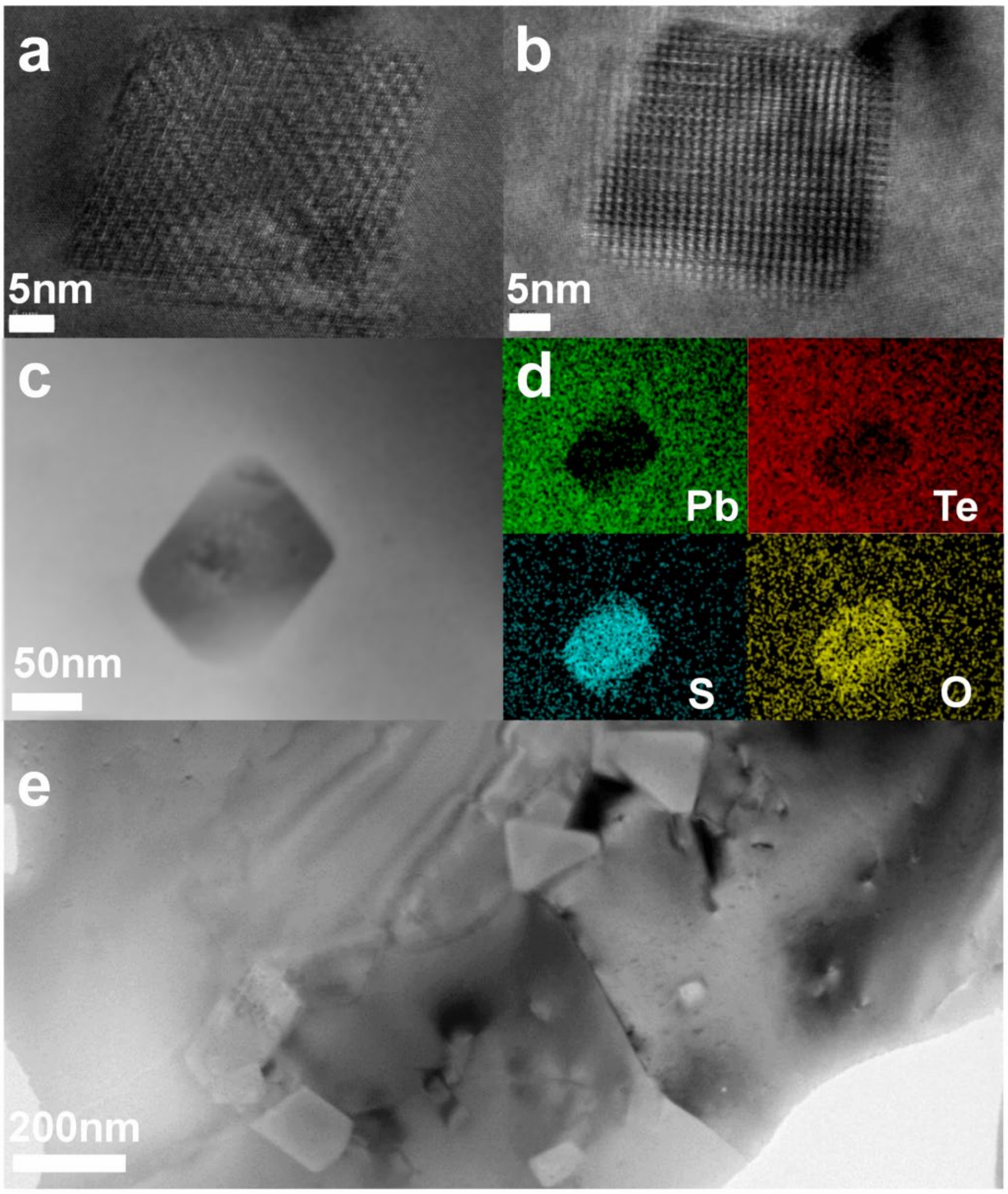

Supplementary Fig. 7 | TEM micrographs of Sb_6O_{13} nanoinclusions in **(a-b)** PbTe-1.5wt.%TeO_2-1.8wt.%Sb sample and **(c-e)** PbTe-1.5wt.%TeO_2-2.16wt.%Sb sample.

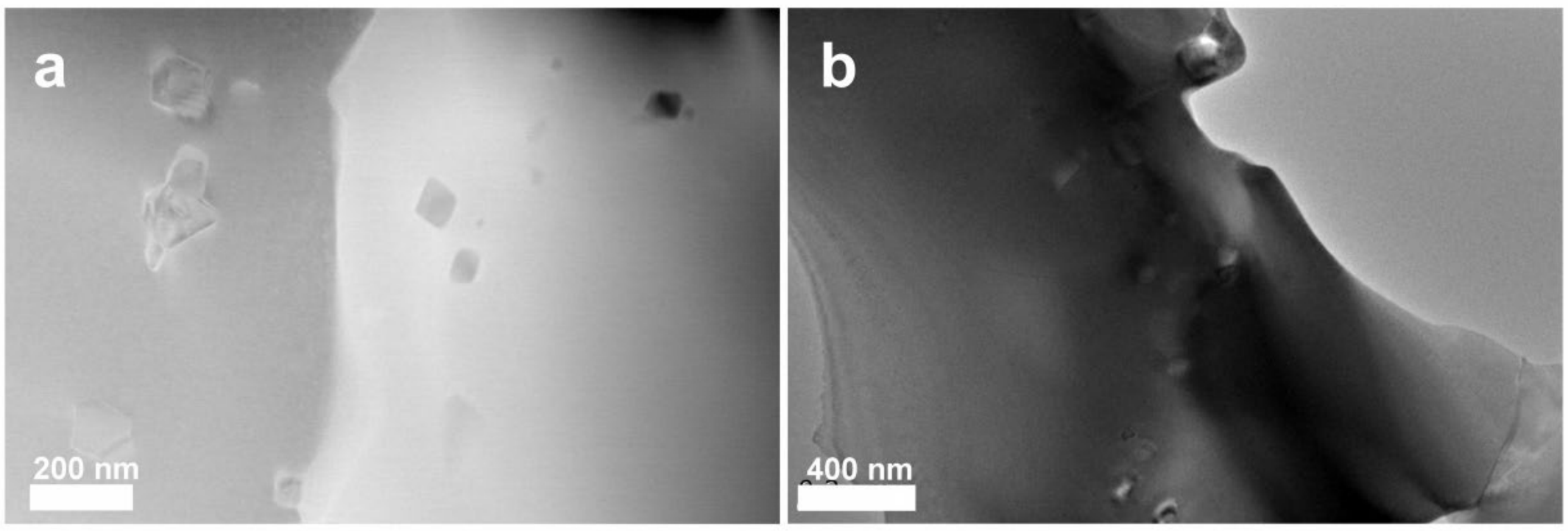

Supplementary Fig. 8 | Representative large area TEM micrographs showing the spatial distribution of Sb_6O_{13} nanoprecipitates within the PbTe matrix.

5. Total thermal conductivity and electronic thermal conductivity

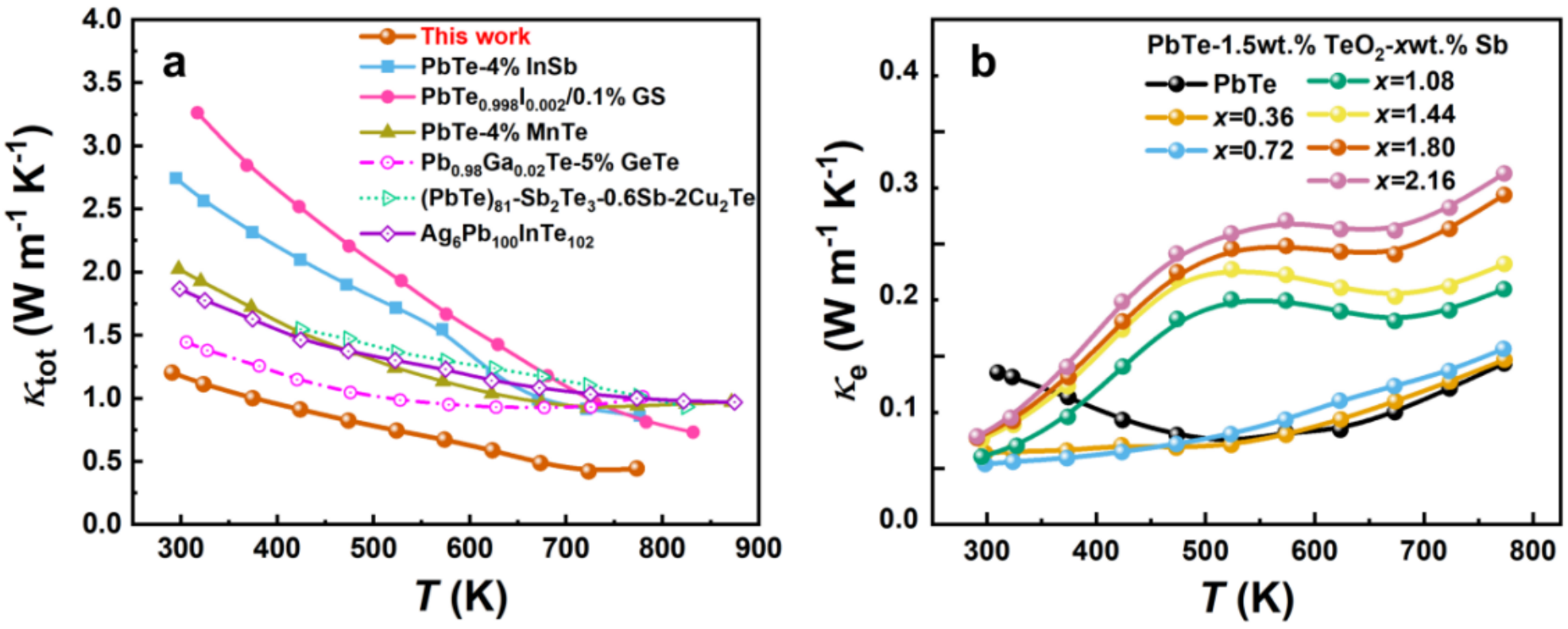

Supplementary Fig. 9 | (a) Temperature dependent total thermal conductivity κ_{tot} for PbTe-1.5wt.%TeO_2-1.8wt.%Sb sample compared with the typical reported results[3-8]; **(b)** Temperature dependent electronic thermal conductivity κ_e for PbTe-1.5wt.%TeO_2-*x*wt.%Sb samples.

6. Calculation details of lattice thermal conductivity *via* Debye-Callaway model

The variation of lattice thermal conductivity κ_L at different temperature and the spectral lattice thermal conductivity κ_s can be calculated by using the Debye-Callaway model as given below[9, 10]:

$$\kappa_L = \frac{4\pi k_B^4 T^3}{v h^3} \int_0^{\theta_D/T} \tau_T \frac{z^4 e^z}{(e^z-1)^2} dz, \tag{8}$$

$$\kappa_s = \frac{4\pi k_B^4 T^3}{v h^3} \tau_T \frac{z^4 e^z}{(e^z-1)^2}, \tag{9}$$

$$\tau_T^{-1} = \tau_U^{-1} + \tau_N^{-1} + \tau_B^{-1} + \tau_{PD}^{-1} + \tau_{DS}^{-1} + \tau_{NP}^{-1}. \tag{10}$$

Where τ_U, τ_N, τ_B, τ_{PD}, τ_{DS} and τ_{NP} are relaxation time corresponding to the scattering from phonon-phonon U- and N-process (U+N), grain/phase boundaries (B), vacancies/alloy elements (point defects, PD), dislocation strain fields and dislocation cores (DS), and nanoprecipitates (NP), respectively. The expression of the different scattering mechanisms are shown below.

The relaxation time corresponds to Umklapp phonon scattering:

$$\tau_U^{-1} = \frac{\hbar \gamma^2 \omega^2 T}{\bar{M} v_s^2 \theta_D} e^{-\theta_D/3T} \tag{11}$$

The relaxation time corresponds to Normal phonon scattering:

$$\tau_N^{-1} = \beta \tau_U^{-1} \tag{12}$$

The relaxation time corresponds to grain/phase boundaries phonon scattering:

$$\tau_B^{-1} = \frac{v_s}{l} \tag{13}$$

The relaxation time corresponds to point defects phonon scattering:

$$\tau_{PD}^{-1} = \frac{\Gamma \bar{V}}{4\pi v_s^3} \omega^4 \tag{14}$$

The relaxation time corresponds to dislocation strain fields and dislocation cores phonon scattering:

$$\tau_{\mathrm{DS}}^{-1} = 0.6B_{\mathrm{D}}^{2}N_{\mathrm{D}}\gamma^{2}\omega\left\{\frac{1}{2}+\frac{1}{24}\left(\frac{1-2r}{1-r}\right)^{2}\left[1+\sqrt{2}\left(\frac{v_{\mathrm{L}}}{v_{\mathrm{T}}}\right)\right]\right\}+N_{\mathrm{D}}\frac{V^{\frac{4}{3}}}{v_{\mathrm{s}}^{2}}\omega^{3} \qquad (15)$$

The relaxation time corresponds to nanoprecipitates phonon scattering:

$$\tau_{\mathrm{NP}}^{-1} = v_{\mathrm{s}}\left[(2\pi R^{2})^{-1}+\left(\pi R^{2}\frac{4}{9}\left(\frac{\Delta D}{D}\right)^{2}\left(\frac{\omega R}{v_{\mathrm{s}}}\right)^{4}\right)^{-1}\right]^{-1}N_{\mathrm{P}} \qquad (16)$$

To avoid unreliable physics parameters from TEM images (such as the dislocation density N_{D}, the number density of nanoprecipitates N_{P}), prefactors were introduced. The expression of the different scattering mechanisms can be rewritten as below:

The relaxation time corresponds to Umklapp and Normal phonon scattering:

$$\tau_{U}^{-1}+\tau_{N}^{-1} = A_{1}\omega^{2}Te^{-\theta_{\mathrm{D}}/3T} \qquad (17)$$

The relaxation time corresponds to point defects phonon scattering:

$$\tau_{PD}^{-1} = A_{2}\omega^{4} \qquad (18)$$

The relaxation time corresponds to dislocation strain fields and dislocation cores phonon scattering:

$$\tau_{DS}^{-1} = A_{3}\omega + A_{4}\omega^{3} \qquad (19)$$

Thus, the total relaxation time can be expressed by:

$$\tau_{\mathrm{T}}^{-1} = A_{1}\omega^{2}Te^{-\frac{\theta_{\mathrm{D}}}{3T}}+A_{2}\omega^{4}+A_{3}\omega+A_{4}\omega^{3}+\frac{v_{\mathrm{s}}}{l}$$
$$+v_{\mathrm{s}}\left[(2\pi R^{2})^{-1}+\left(\pi R^{2}\frac{4}{9}\left(\frac{\Delta D}{D}\right)^{2}\left(\frac{\omega R}{v_{\mathrm{s}}}\right)^{4}\right)\right]^{-1}N_{\mathrm{P}} \qquad (20)$$

Where A_1, A_2, A_3 and A_4 are prefactors corresponding to the scattering from phonon-phonon U- and N-process, vacancies/alloy elements (point defects), dislocation strain fields and dislocation cores, respectively. θ_{D} is Debye temperature (θ_{D}=136 K)[11], v_{s} is average sound velocity (1770 m s^{-1})[11], l is the mean spatial distance for the phase and twin boundaries, R is the average radius of nanoprecipitates (R≈30 nm for PbTe-1.5wt.%TeO_2-$x$wt.%Sb samples with x=0.36-1.8 and 60 nm for the sample with x=2.16) and $\Delta d/d=(d_{\mathrm{PbTe}}-d_{\mathrm{Sb6O13}})/d_{\mathrm{PbTe}}$=0.31. The number density of Sb_6O_{13} nanoprecipitates, denoted as N_{p}, is determined through a combination of macroscopic phase quantification and microscopic statistical analysis. First, the mass fraction of the Sb_6O_{13} phase is obtained from the Rietveld refinement of the X-ray diffraction patterns. This mass fraction is converted into a volume fraction, ϕ, using the theoretical densities of the PbTe matrix and the Sb_6O_{13} precipitates. Subsequently, the average radius, R, of the Sb_6O_{13} nanoinclusions is determined by statistically analyzing multiple high resolution TEM images. Assuming a spherical geometry for the

precipitates, the average volume of a single nanoinclusion is calculated as $V_p = 4/3\pi R^3$. Finally, the number density N_p is derived using the relation $N_p = \phi/V_p$. This experimental approach ensures that the parameters used in our thermoelectric transport modeling are grounded in the actual microstructure of the synthesized samples. The parameters used in the Debye-Callaway model for all investigated composite samples are provided in **Supplementary Table 3**.

Supplementary Table 3. Parameters used in the Debye-Callaway calculation for PbTe-1.5wt.%TeO_2-*x*wt.%Sb samples.

Parameters	*x*=0	*x*=0.36	*x*=0.72	*x*=1.08	*x*=1.44	*x*=1.80	*x*=2.16
**A_1 (10^{-17}sK^{-1})**	1.04	1.07	1.15	1.28	1.35	1.40	1.45
**A_2 (10^{-41}s^3)**	1.1	1.2	2.1	4.19	6.03	8.80	8.90
**A_3 (10^{-3})**	--	0.12	0.28	2.42	6.43	12	5.20
**A_4 (10^{-28})**	--	0.27	0.53	1.35	1.9	2.7	1.2
**L (10^2 nm)**	30	25	20	15	10	7	9
**N_P (10^{21} m^{-3})**	--	0.16	0.82	2.75	4.3	6.5	1.5

To decode the precise physical scattering characteristics of individual dislocations and establish a parameter-free microstructural validation for the Debye-Callaway model, Ham's statistical linear-intercept method (N_D = 2N/(L×t)) was applied to the representative TEM micrographs across different compositions (Supplementary Fig. 5). Based on the quantified N_D, three crucial physical indices characterizing individual dislocation-phonon interactions are further decoded under a representative thermal phonon frequency peak (ω≈1.5 THz) for PbTe at elevated temperatures:

Dislocation Network Spacing (d_{dis}): Geometrically, the average physical distance between the entangled dislocation lines within the matrix lattice is geometrically defined by $d_{dis} = 1/\sqrt{N_D}$.

Dislocation-Induced Phonon Mean Free Path (Λ_{dis}): According to the Boltzmann transport relation, the frequency-dependent phonon mean free path governed by dislocation strain fields is expressed as $\Lambda_{dis}(\omega) = v_s \times \tau_{DS}(\omega)$, where τ_{DS} is evaluated from the strain-field scattering term in Supplementary formula (15).

Individual Scattering Cross-Section (σ_{dis}): For line-like defects, the effective scattering cross-section (the effective scattering diameter per unit length) is defined by $\sigma_{dis}(\omega) = 1 / (N_D \times \Lambda_{dis}(\omega))$. The comprehensively quantified physical characteristics of individual dislocation defects are

summarized in Supplementary Table 4.

Supplementary Table 4. Detailed quantification of individual dislocation characteristics, including experimental density (N_D), spatial spacing (d_{dis}), phonon mean free path (Λ_{dis}), and scattering cross-section (σ_{dis}) evaluated at ω ≈1.5 THz.

Nominal composition	N_D(cm^{-2})	d_{dis} (nm)	Λ_{dis} (nm)	σ_{dis} (nm)
**PbTe-1.5wt.%TeO$_2$-0.36wt.%Sb**	1.14×10^{10}	~93.7	~312	~28
**PbTe-1.5wt.%TeO$_2$-1.08wt.%Sb**	3.89×10^{10}	~50.7	~91	~28
**PbTe-1.5wt.%TeO$_2$-1.80wt.%Sb**	6.99×10^{10}	~37.8	~51	~28

As tabulated, since the fundamental physical constants (Burgers vector and Grüneisen parameter) ruling the scattering cross-section remain invariant in the fixed PbTe matrix, σ_{dis} manifests as an intrinsic scattering feature size (~28 nm). Remarkably, for the peak-performance sample (x = 1.80), the actual physical network spacing ($d_{dis} \approx$ 37.8 nm) is driven down to approach this intrinsic scattering cross-section, inducing dense spatial overlapping of adjacent dislocation strain fields. This structural convergence successfully interprets the drastic suppression of lattice thermal conductivity on a rigorous physical basis.

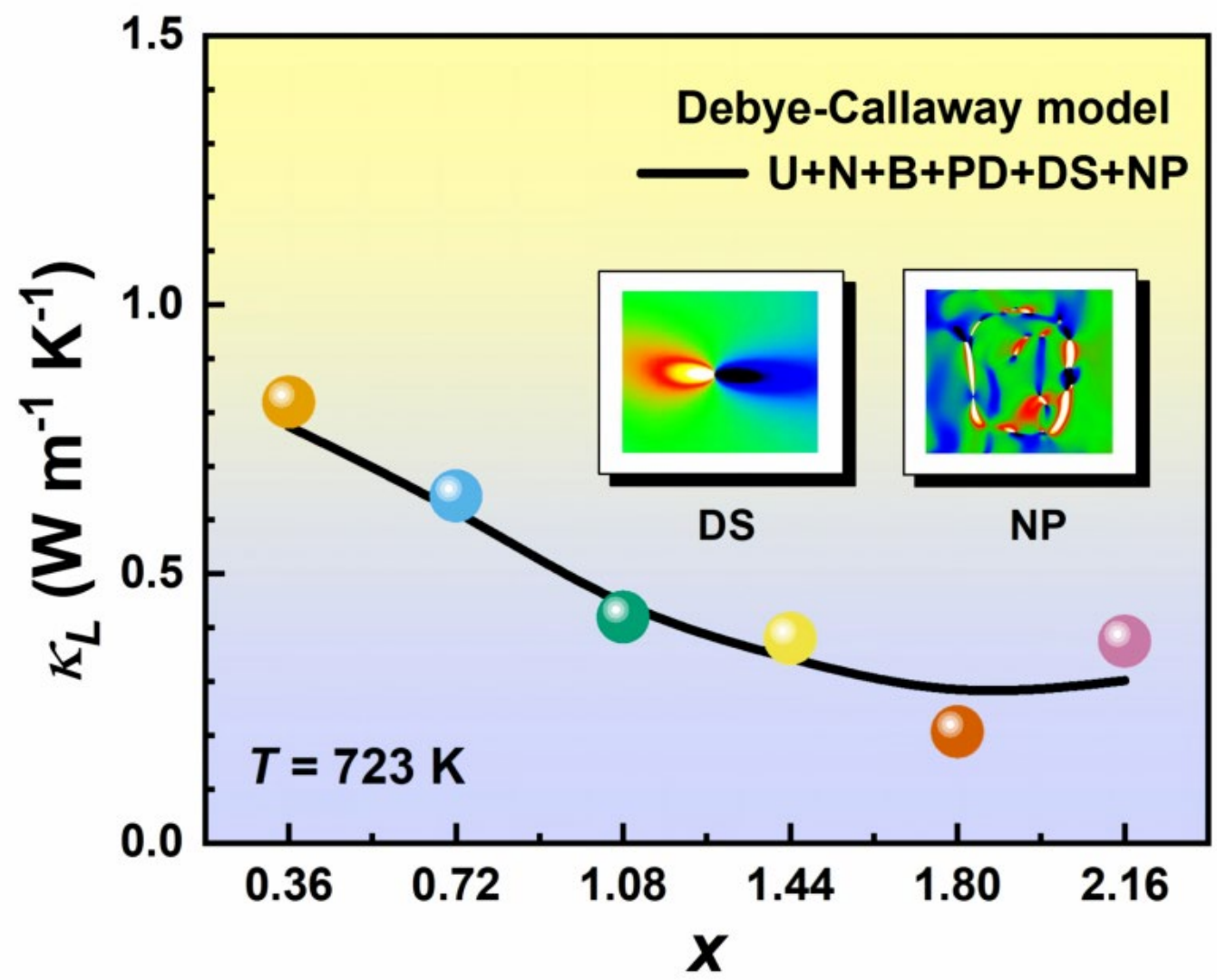

Supplementary Fig. 10 | Variation of lattice thermal conductivity at 723 K with x for PbTe-1.5wt.%TeO$_2$-$x$wt.%Sb composite samples. The symbols show the experimental results in this work, while the black curve show the calculated results based on Debye-Callaway model, including various phonon scattering models.

7. **Analysis of the electrical transport properties**

To specifically reveal the electrical transport properties of PbTe-1.5wt.%TeO_2-*x*wt.%Sb samples, we performed Hall measurements to study the electron concentration and mobility. The electron concentration in all samples shows the same trend with increasing *T*, as observed in **Supplementary Fig. 11a**. One can see that *n* first slowly rises with increasing *T* until approximately 673 K, and then it rises rapidly as temperature increases further, indicating that thermal excitation of electrons in impurity and/or defect energy levels predominate in electrical transport at high temperature range[6, 12, 13]. The rapid increment behavior of *n* offers an explanation for the anomalies around 673 K in σ and *S* (**Supplementary Figs. 4a-b**). **Supplementary Fig. 11b** shows the temperature dependence of carrier mobility for PbTe-1.5wt.%TeO_2-*x*wt.%Sb samples. One can see that the mobility initially rises with increasing temperature and then decreases as temperature increases further. Consequently, the temperature dependence of carrier mobility explicitly reveals the carrier scattering mechanism. According to Boltzmann transport theory, the temperature dependent $1/\mu$ considering several possible scattering mechanisms can be written as[14]

$$\frac{1}{\mu} = \alpha_l T^{3/2} + \alpha_i T^{-3/2} + \alpha_d T^{-1}, \tag{21}$$

where α is a temperature independent parameter and *l*, *i*, *d* denote the contributions to the scattering from the lattice, impurities and dislocations, respectively. Generally, the carrier mobility follows $\mu \approx T^{\gamma}$, and the exponents γ of -3/2, 3/2 and 1 indicate that the scattering is dominated by acoustic phonon scattering, ionized impurity scattering and dislocation scattering, respectively. Here, γ is about 3/2 for PbTe-1.5wt.%TeO_2-*x*wt.%Sb samples at lower temperature range, and then close to -2 at higher temperature range, suggesting that the carrier scattering mechanisms are ionized impurity scattering at lower temperature range and acoustic phonon scattering ($\gamma \leq$ -3/2)[15, 16] at higher temperature range. The different scattering mechanisms lead to the "increase first-decrease afterwards" behavior of μ, which should be the origin of kink at ≈523 K for σ. Combining the variation of *n* and μ, the abnormal behaviors of σ at ≈523 and 673 K are not hard to understand.

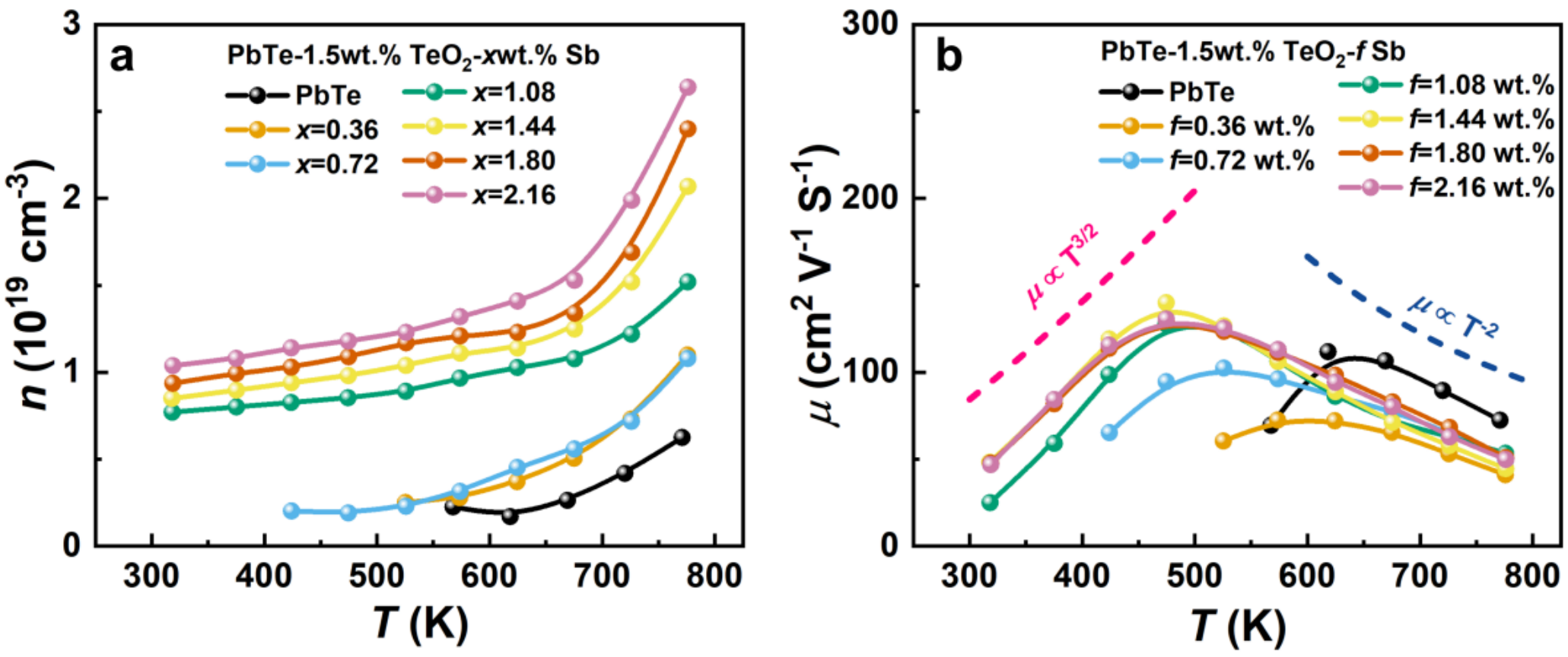

Supplementary Fig. 11 | Temperature dependent **(a)** electron concentration and **(b)** electron mobility for PbTe-1.5wt.%TeO_2-$x$wt.%Sb samples.

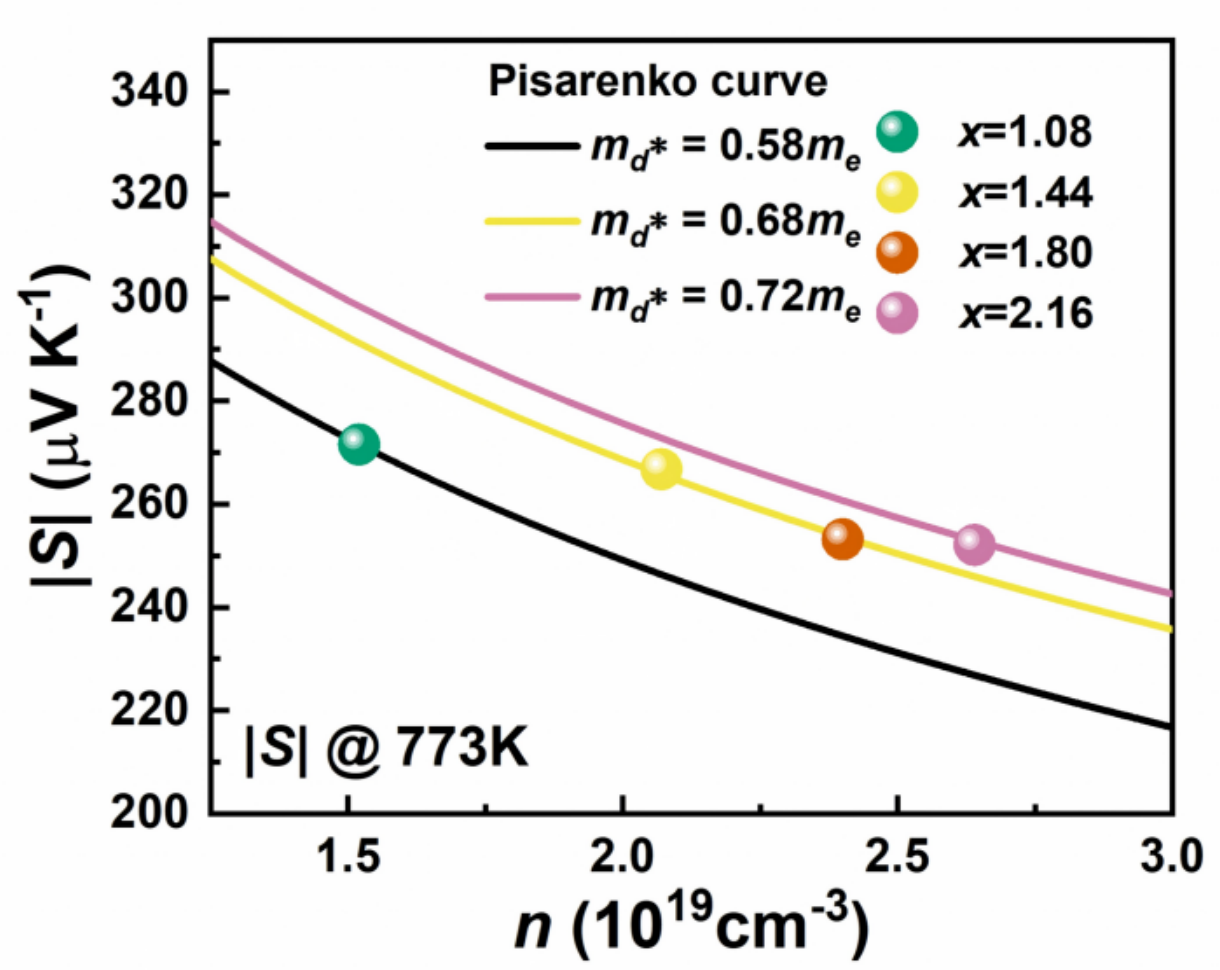

Supplementary Fig. 12 | Variation of thermopower S with electron concentration n for PbTe-1.5wt.%TeO_2-$x$wt.%Sb samples (x = 1.08, 1.44, 1.8, and 2.16), in which the lines are the Pisarenko curves obtained by SPB model.

8. **Band Structure and PDOS of PbTe and $Pb_{0.96875}Sb_{0.03125}Te$**

To clarify the mechanisms of increased m_d^*, density functional theory based first principle calculation was carried out to explore the effects of Sb substitution on electronic band structure and electronic density of states (DOS). As shown in **Supplementary Fig. 13**, the calculated band structure for $Pb_{0.96875}Sb_{0.03125}Te$ shows that impurity bands (red points in **Supplementary Fig. 13b**) are mixed with the host conduction band and shows shallow donor feature. Besides, the conduction bands of $Pb_{0.96875}Sb_{0.03125}Te$ become flatter than that of the pristine PbTe, which can increase the DOS around conduction band minimum. Besides, one can see from **Supplementary Fig. 13c** that the substitution of Sb at Pb sites creates an obviously large DOS peak at the Fermi level (E_F) and clearly modifies the DOS. The remarkable increment in DOS at E_F is mainly due to the contribution of Sb 5p orbit (**Supplementary Fig. 13e**), which should be the physical origin of the enhanced m_d^* and thermopower.

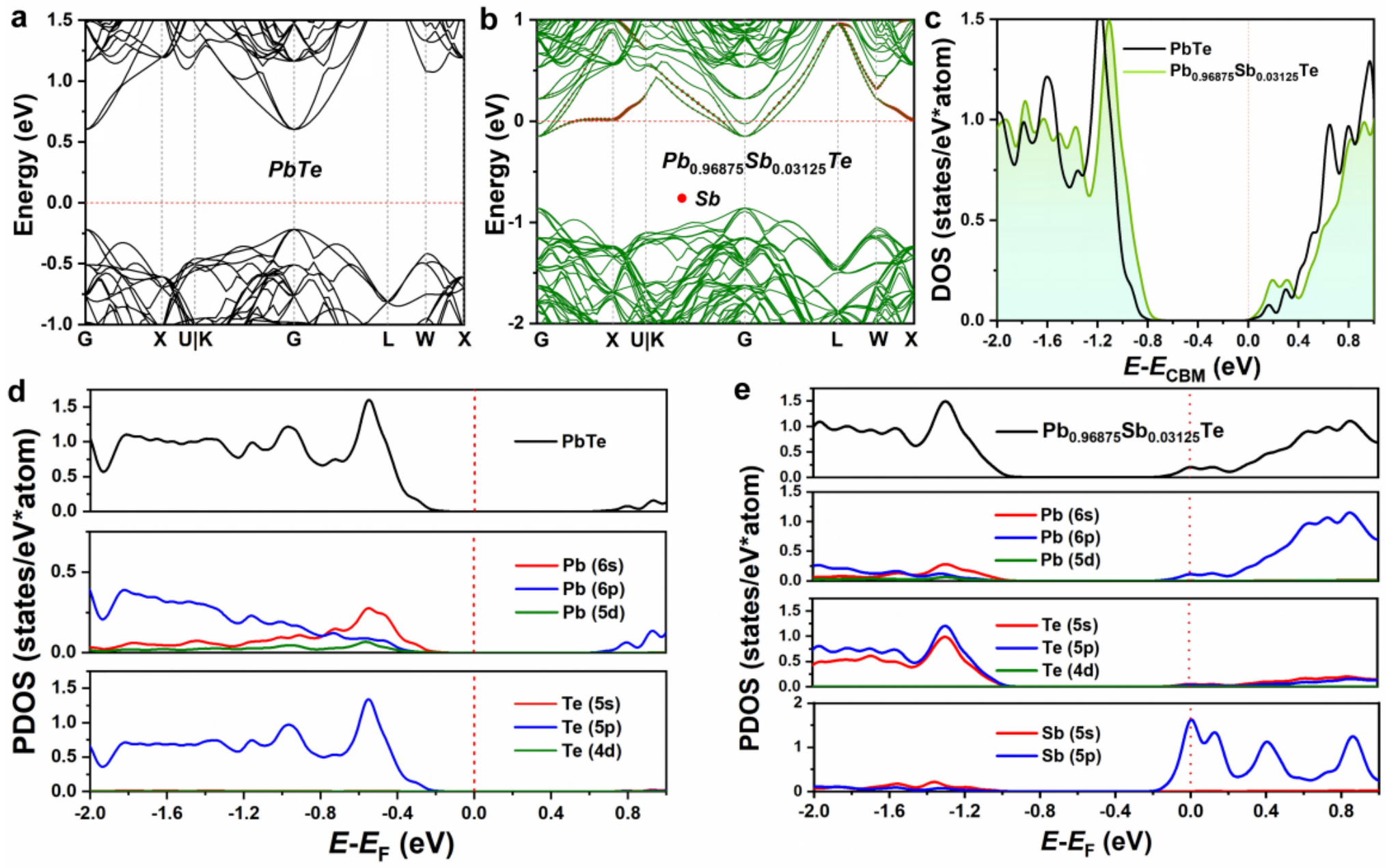

Supplementary Fig. 13 | Band structure of PbTe **(a)** and $Pb_{0.96875}Sb_{0.03125}Te$ **(b)**. **(c)** total DOS of PbTe and $Pb_{0.96875}Sb_{0.03125}Te$. PDOS of PbTe **(d)** and $Pb_{0.96875}Sb_{0.03125}Te$ **(e)**.

9. **Data repeatability and thermal stability**

As presented in Supplementary Fig. 14, independent synthesis batches confirm that the corresponding thermoelectric transport properties are robustly reproducible. This rules out the possibility that the high-strain microstructure arises from random transient deformation.

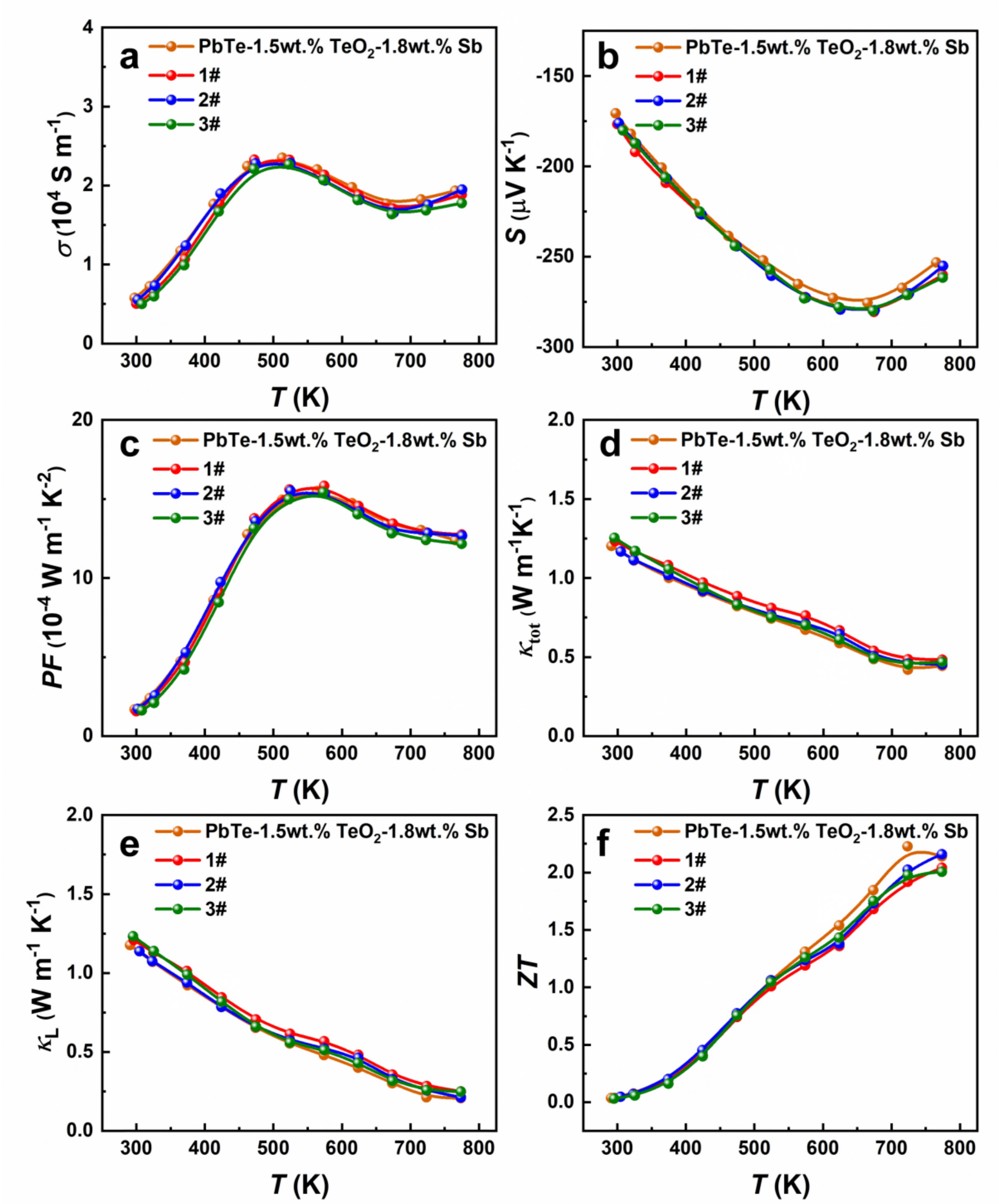

Supplementary Fig. 14 | Data repeatability of **(a)** electrical conductivity σ, **(b)** thermopower S, **(c)** power factor PF, **(d)** total thermal conductivity κ_{tot}, **(e)** lattice thermal conductivity κ_L and **(f)** ZT for PbTe-1.5wt.%TeO_2-1.8wt.%Sb composite. Note: The three samples for the repeatability test were from an independent synthesis batch, separate from the samples discussed in the main text, demonstrating excellent batch-to-batch reproducibility.

We tested the optimal PbTe-1.5 wt.% TeO_2-1.8 wt.% Sb sample over a full heating–cooling–reheating cycle (~13h) covering the entire working temperature range of 300-773 K. As demonstrated in Supplementary Fig. 15, no obvious changes were detected in electrical conductivity, thermopower, total thermal conductivity, or the peak *ZT* value after repeated high-temperature exposure.

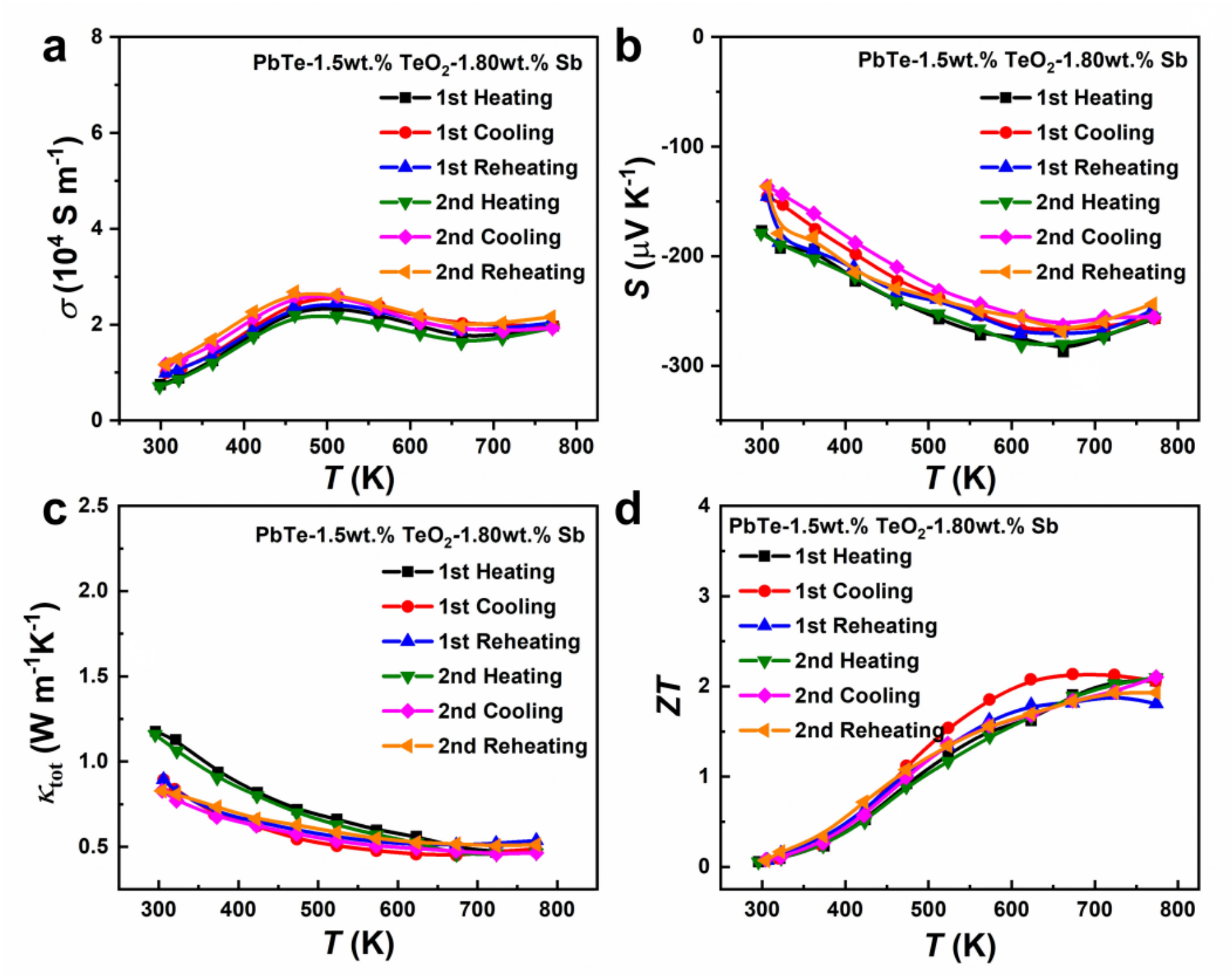

Supplementary Fig. 15 | Thermal stability of **(a)** electrical conductivity σ, **(b)** thermopower S, **(c)** total thermal conductivity κ_{tot}, and **(d)** ZT for PbTe-1.5wt.%TeO_2-1.8wt.%Sb composite.

10. **Three-dimensional finite-element simulation of electrical output and conversion efficiency**

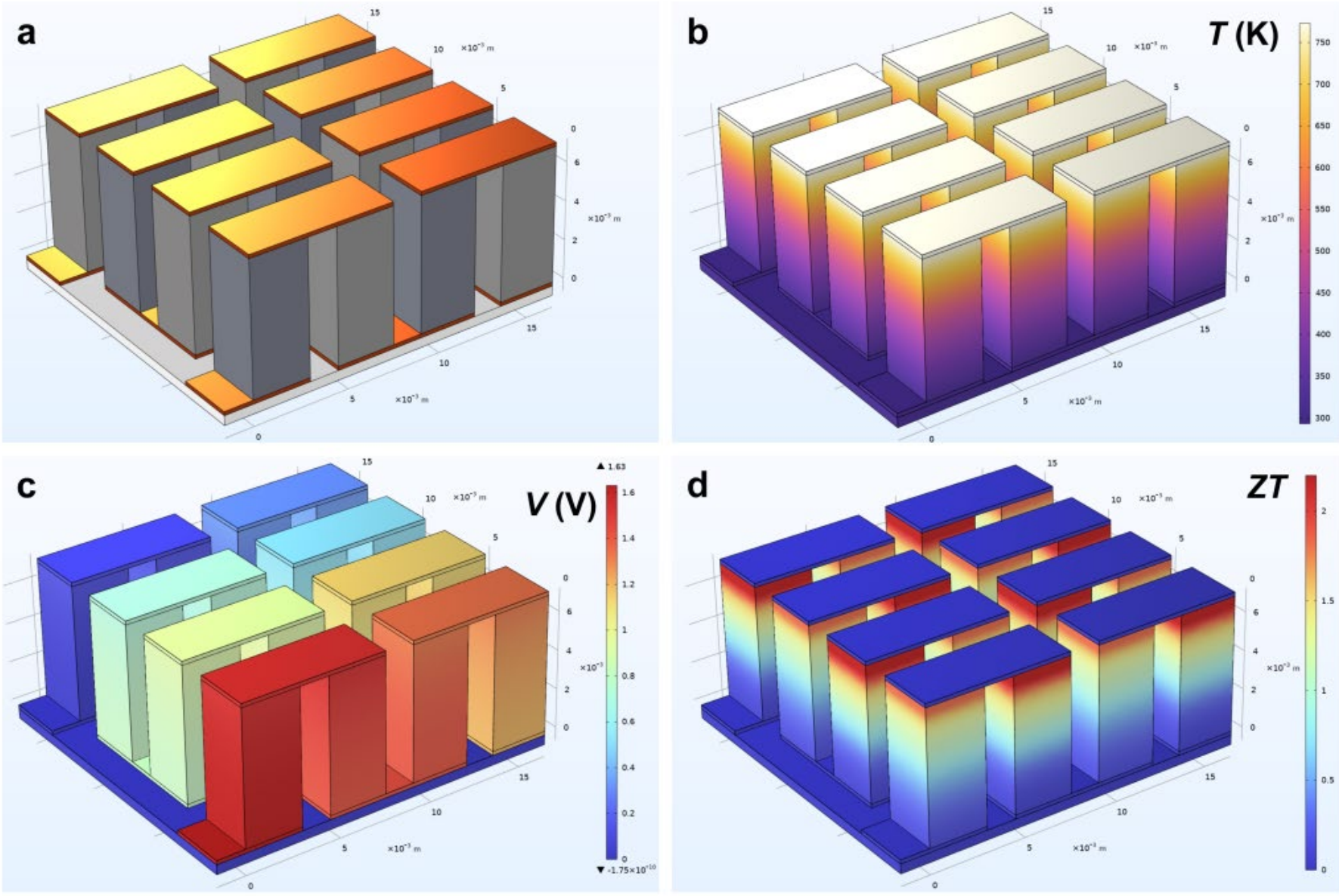

Supplementary Fig. 16 | Typical results of numerical simulations for the PbTe-based module (PbTe-1.5wt.%TeO_2-1.8wt.%Sb (n-type) and PbTe-4mol%SrTe-2mol%Na (p-type)[17]) for hot side at 773 K and cold side at 300 K. **(a)** Geometrical configuration, **(b)** temperature *T*, **(c)** electrical potential V, and **(d)** thermoelectric figure of merit *ZT*. The length, width, and height of the legs are 3, 3 and 7mm, respectively.

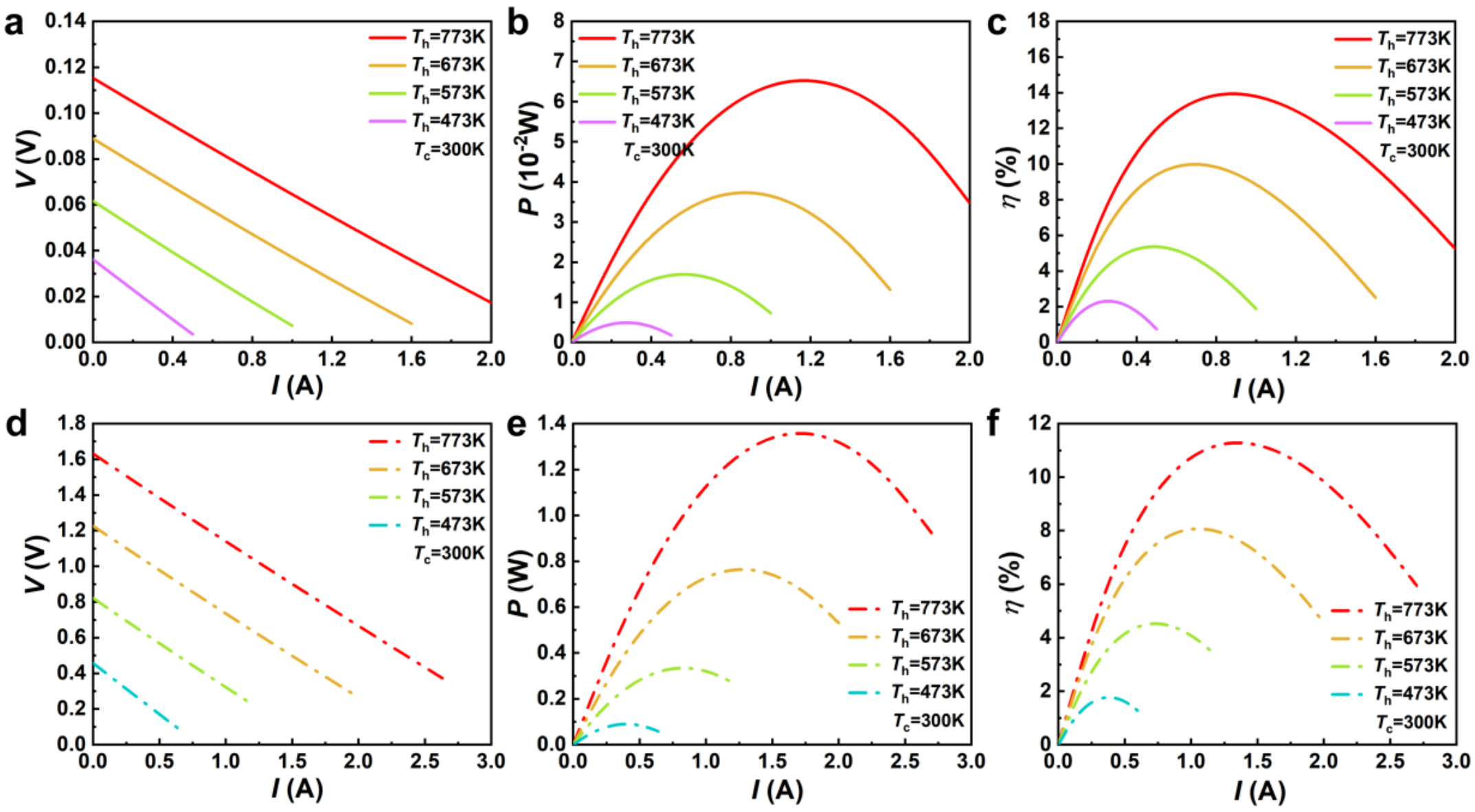

Supplementary Fig. 17 | Simulated terminal voltage V **(a)**, electrical power output P **(b)**, and conversion efficiency η **(c)** of single-leg PbTe module (PbTe-1.5wt.%TeO_2-1.8wt.%Sb (n-type)); simulated terminal voltage V **(d)**, electrical power output P **(e)**, and conversion efficiency η **(f)** of 8-couple PbTe-based module (PbTe-1.5wt.%TeO_2-1.8wt.%Sb (n-type) and PbTe-4mol%SrTe-2mol%Na (p-type)[17]); the hot-side temperature (T_h) was changed from 773 K to 473 K, the cold-side temperature (T_c) was maintained at 300 K.

References

1. Kashyap, K. T. *et al.* On Peierls Nabarro stress in Iron. *Computational Materials Science* **56**, 172-173 (2012).
2. Wu, Z.-j. *et al.* Crystal structures and elastic properties of superhard IrN2 and IrN3 from first principles. *Physical Review B* **76**, (2007).
3. Qiu, W. *et al.* Enhancing the figure of merit of n-type PbTe materials through multi-scale graphene induced interfacial engineering. *Nano Today* **39**, (2021).
4. Liu, S. *et al.* Coherent Sb/CuTe Core/Shell Nanostructure with Large Strain Contrast Boosting the Thermoelectric Performance of n-Type PbTe. *Advanced Functional Materials* **31**, (2020).
5. Xiao, Y. *et al.* Amphoteric Indium Enables Carrier Engineering to Enhance the Power Factor and Thermoelectric Performance in n-Type AgnPb100InnTe100+2n (LIST). *Advanced Energy Materials* **9**, (2019).
6. Luo, Z. Z. *et al.* High Figure of Merit in Gallium-Doped Nanostructured n-Type PbTe-xGeTe with Midgap States. *J Am Chem Soc* **141**, 16169-16177 (2019).
7. Xiao, Y. *et al.* Realizing high performance n-type PbTe by synergistically optimizing effective mass and carrier mobility and suppressing bipolar thermal conductivity. *Energy & Environmental Science* **11**, 2486-2495 (2018).
8. Zhang, J. *et al.* Extraordinary Thermoelectric Performance Realized in n-Type PbTe through Multiphase Nanostructure Engineering. *Adv Mater* **29**, (2017).
9. Callaway, J. Model for Lattice Thermal Conductivity at Low Temperatures. *Physical Review* **113**, 1046-1051 (1959).
10. Kim, W. *et al.* Thermal conductivity reduction and thermoelectric figure of merit increase by embedding nanoparticles in crystalline semiconductors. *Phys Rev Lett* **96**, 045901 (2006).
11. Yu, Y. *et al.* Ag-Segregation to Dislocations in PbTe-Based Thermoelectric Materials. *ACS Appl Mater Interfaces* **10**, 3609-3615 (2018).
12. Fu, L. *et al.* Large enhancement of thermoelectric properties in n-type PbTe via dual-site point defects. *Energy & Environmental Science* **10**, 2030-2040 (2017).
13. Zhang, J. *et al.* Design of Domain Structure and Realization of Ultralow Thermal Conductivity for Record-High Thermoelectric Performance in Chalcopyrite. *Adv Mater* **31**, e1905210 (2019).
14. Dexter, D. L., Seitz, F. Effects of Dislocations on Mobilities in Semiconductors. *Physical Review* **86**, 964-965 (1952).
15. Pei, Y. *et al.* Low effective mass leading to high thermoelectric performance. *Energy & Environmental Science* **5**, (2012).
16. Su, X. *et al.* Weak Electron Phonon Coupling and Deep Level Impurity for High Thermoelectric Performance Pb1−xGaxTe. *Advanced Energy Materials* **8**, (2018).
17. Biswas, K. *et al.* High-performance bulk thermoelectrics with all-scale hierarchical architectures. *Nature* **489**, 414-418 (2012).
