# Supplementary material for: Manipulating Peierls-Nabarro stress to realize extraordinary thermoelectric performance in n-type PbTe
Source: Nat Commun. 2026 Sep 4;17:10536. doi: 10.1038/s41467-026-77575-0 (PMC13642244; doi:10.1038/s41467-026-77575-0)
Supplement: Supplementary file 2 [file 41467_2026_77575_MOESM2_ESM.pdf]

nature portfolio

Peer Review File

Manipulating Peierls-Nabarro stress to realize extraordinary thermoelectric performance in n-type PbTe

Corresponding Author: Professor Jian Zhang

This file contains all reviewer reports in order by version, followed by all author rebuttals in order by version.

Version 0:

Reviewer comments:

Reviewer #1

(Remarks to the Author)
This study presents a compelling defect-engineering strategy to enhance the thermoelectric performance of PbTe by coupling lattice softening with electronic structure modification. By manipulating Peierls-Nabarro stress through solute alloying, the authors induce a high density of dislocations and subgrain boundaries that effectively scatter phonons, while the in-situ formation of Sb_6O_{13} nanoinclusions introduces additional multiscale phonon scattering. Simultaneously, Sb doping increases the electronic density of states, leading to an improved power factor. The resulting combination of ultralow lattice thermal conductivity and high ZT (~2.2) highlights the effectiveness of integrating defect mechanics with band engineering and provides valuable insight into designing high-performance thermoelectric materials beyond conventional alloying approaches.
The article can be accepted after major revision. I suggest the authors address the following comments:
Major comments
1. Transition between SEM-EDS and TEM observations
There is insufficient transition in the discussion from SEM-EDS results to TEM observations regarding the appearance of Sb_6O_{13} nanophases (nanoprecipitates). It would be helpful to explicitly clarify that, due to resolution limitations, SEM-EDS indicates a uniform elemental distribution in the 1.8 wt.% Sb sample (Fig. S4), whereas higher-resolution TEM clearly reveals the presence of Sb_6O_{13} nanophases. This transition from apparent compositional uniformity to the PbTe/Sb_6O_{13} two-phase microstructure is also not clearly described in the discussion of Fig. 1g.
2. Quantification of Sb_6O_{13} nanophase abundance
There is a lack of quantitative data on the abundance of Sb_6O_{13} nanophases. Figures 2a and 2b suggest a periodic microstructure of Sb_6O_{13} nanophases within a PbTe matrix grain; however, most nanoprecipitates appear to be located near PbTe/PbTe grain boundaries, with only a limited number observed within grain interiors. Quantification of precipitate density and spatial distribution would provide a stronger link to the calculated spectral lattice thermal conductivity (Ks). Additionally, the observed morphology suggests that Sb_6O_{13} nanoprecipitates may form a Widmanstätten-like pattern within the PbTe matrix, with rows seemingly aligned parallel to the {100} direction of PbTe. This hypothesis should be verified or discussed more carefully.
In lines 143–144 (Supplementary Information), the authors state that prefactors were introduced to avoid uncertainties arising from TEM-based precipitate and dislocation density calculations.
a) Although Table S3 lists Np values, it is not clear whether these values are derived directly from TEM image analysis or assumed for modeling purposes. This should be clarified.
3. Reduction in effective number of Sb_6O_{13} nanophases
In lines 206–207 of the main text, the authors state that there is a reduction in the effective number of Sb_6O_{13} nanophases; however, no clear quantification or supporting analysis is provided. Please include quantitative evidence or clarify the basis for this statement.
4. Phonon scattering parameters
For both Sb_6O_{13} nanophase phonon scattering and dislocation scattering mechanisms, it would significantly strengthen the manuscript if the authors provided more detailed quantitative information on defect densities and a more comprehensive characterization of Sb_6O_{13} spatial distribution.

Minor comments and typographical corrections
1. Please clarify in the Fig. S6 caption that all TEM and GPA analyses were performed on the PbTe matrix.

2. Line 118 (main text): revise to “Pb vacancies.”
3. Line 154 (main text): revise to “are close.”
4. Line 252 (main text): revise to “effective mass.”
5. Line 152 (main text): the text states that only a coherent PbTe/Sb6O13 interface is present; however, Fig. 2d shows a coherent interface, whereas Fig. 2h clearly shows a semi-coherent PbTe/Sb6O13 interface, as also indicated by the figure labels. Please revise the text to reflect the presence of both interface types.

Reviewer #2

(Remarks to the Author)
This paper presents a method for producing high-performance thermoelectric (TE) lead telluride (PbTe)-based alloys by controlling multidimensional defects. Various dimensional defects scatter in ways that conflict with thermal and electrical conductivity, creating an inevitable challenge in achieving an optimized balance of them. Currently, many groups around the world are working to improve the performance of TE energy-conversion PbTe alloys, especially n-type ones.
The wide-ranging, thorough results reported in the paper are expected to contribute significantly to the realization of excellent n-type PbTe alloys. While this paper does not present new physics, it does provide optimized technological parameters for TE processes. Indeed, the authors emphasize achieving the best possible results among the materials. This review was conducted to understand the mechanisms underlying in the processes. In particular, the contribution of dislocations is examined in light of the paper’s title.
The authors theoretically estimated the reduction of the Peierls-Nabarro (PN) stress in the PbTe alloys to introduce many dislocations. These alloys are known to be narrow-gap semiconductors, which are typically soft materials, even at room temperature (Acta Mater. 61, 294 (203) by Kamimura et al.). The PN stress is defined at 0 K and decreases at elevated temperatures, which is where the present samples were sintered. In this case, the dislocation densities are supposed to be similar in the alloys and to depend minimally on the composition. Thus, their contribution to the thermal conductivities through the phonon scattering is questionable. Instead, such alloying leads to mechanical solution hardening. To refuse this claim, the authors could demonstrate the density of dislocations in the samples with different compositions. This paper does not mention the effects of dislocations on electrical conductivity.
In this research, the alloys were prepared by sintering, raising the question of the size of the grains in the prepared poly-crystals. According to G.A. Slack (J. Appl. Phys. 70,12694 (1991), the barrier effects of grain boundaries against thermal conductivity only appear when the grain size is smaller than 1 μm.
Can the reported TE properties be understood as resulting from various scatterings caused by the multidimensional defects introduced in the alloys? These phenomena are known as the alloying effect of a high-entropy system. Of course, clarifying how these respective defects contribute to the relevant scatterings is important both academically and practically.

The following technological point should be considered:
As noted above, the paper thoroughly reports that the authors obtained the top ZT record in the material system. However, the descriptions are overall detailed, making it difficult to understand the results without the supplementary materials, which include important basic data, such as how the samples were prepared. This may be an attempt to emphasize the accomplishment of obtaining top data. This results in the diffusion of data between the text and supplementary materials. Describing only the most important data, such as TEM observations, thermal and electrical conductivity, and ZT for the specific alloy with the top data in the text, would be much effective. The rest can be shown in the supplementary materials. Additionally, reconsidering the overall text, including figures and English descriptions, is necessary for successful publication.

Reviewer #3

(Remarks to the Author)
PbTe based compounds has been recognized as one of the best thermoelectric materials, and microstructure modification is known as the effective way to improve the performance of this system. Authors reported the change in transport properties with different x content in PbTe-1.5wt.%TeO2-xwt.%Sb, combined with detailed microstructure analysis. The reported data is worth reporting, however some questions about transport analysis need to be answered to clarify their claims. Therefore, I recommend the present work for publication only after addressing the following queries.

1. The introduction lacks sufficient discussion of the roles of dislocations and softening. The rationale behind the chosen experimental compositions (PbTe-1.5wt.%TeO2-xwt.%Sb) is not clearly explained. Please elaborate on how these factors influenced the design of the study.
2. Given that nanostructures, dislocations, grain boundaries, strain, and lattice defects are all present and potentially interacting, it is unclear whether the fittings shown in Figure S9 and Table S3 are free from arbitrariness. The authors should clarify which parts of the interpretation are experimentally confirmed and which parts are inferred or model dependent.
3. The simulation data appears to have the limited endorsement for the conclusion in their current form. Instead of relying on simulations to demonstrate high performance, it may be more convincing to fabricate and evaluate an actual module to claim the higher conversion efficiency.
4. Why is Sb content expressed in wt%? What is the actual composition of the samples? If Sb substituted Te site, where did the excess Te go?
5. While repeatability within a batch appears good, what about reproducibility across different batches? Can similar performance be consistently reproduced in independently synthesized samples?
6. Nanostructures in thermoelectric materials often coarsen or disappear at high temperatures, leading to a loss of

performance. In the present work, the authors suggest that this issue does not apply, but the reasoning behind this claim is not sufficiently explained. Could the authors clarify why the nanostructure is expected to remain stable under high temperature conditions in this system?
7. The manuscript contains unnecessary adverbs such as "apparently," "inevitably," and "obviously." These weaken the scientific tone and should be removed or replaced with more precise wording.

Reviewer #4

(Remarks to the Author)
The authors report on the high thermoelectric performance of n-type PbTe-1.5wt.%TeO2-1.8wt.%Sb material. A simulated conversion efficiency of 14% is calculated for three dimensional single-leg finite elements. The manuscript, together with the supplementary materials, provides a comprehensive and well-documented account of the experimental methods and analytical techniques employed to obtain the reported results and conclusions. The manuscript is well written and of great importance to the materials science community. I would recommend it to be published in Nature Communications after the following points are sufficiently addressed.

1. Figure S7(c) is presented to show the formation of relatively larger nanoinclusions in PbTe-1.5wt.%TeO2-2.16wt.%Sb sample. However, it is not clearly shown. An EDS elemental mapping of Pb, Te, Sb, and O, similar to Figure 2 (b), can better support this argument.

2. Please ensure all acronyms are introduced with their full names upon first mention. For example, the acronyms for FFT and ABF are not addressed in the Supplementary Materials.

3. The authors cite several relevant studies throughout the manuscript; however, references are missing in places where they are particularly needed. For example, the second-to-last line of the second paragraph in the Introduction "which is well below the target of 2.2–2.8" requires an appropriate citation. A similar issue occurs in the third-to-last line of the fourth paragraph of the Introduction "which is about 10-fold larger than that of pristine PbTe", where a supporting reference should be provided.

4. For the 4th paragraph of "Phase and microstructure characterization": Providing a citation for "the theoretical value in PDF database" would be helpful, as it would allow readers to easily locate and verify the referenced data.

5. The claim of in-situ Sb_6O_{13} nanoinclusion formation would benefit from a clearer discussion of the conditions under which this secondary phase forms and whether its presence is consistently reproducible across independently prepared samples. Clarifying whether the repeatability tests were performed will further strengthen the manuscript.

Reviewer #5

(Remarks to the Author)
I co-reviewed this manuscript with one of the reviewers who provided the listed reports. This is part of the Nature Communications initiative to facilitate training in peer review and to provide appropriate recognition for Early Career Researchers who co-review manuscripts.

Version 1:

Reviewer comments:

Reviewer #2

(Remarks to the Author)
I appreciate the structural revisions you have made to the paper in response to my suggestions, and I now have a better understanding of your research strategy. However, I am not satisfied with your response to my previous request for the authors to demonstrate the density of dislocations in samples with different compositions. While the authors did present TEM photographs depending on the composition (Figure R3 in the response and Figure S5 in the supplementary information), they did not provide any numerical characterizations of dislocations or other defects. I estimate the dislocation density to be between 10^9 and 10^{11} cm^{-2} in the samples.
Reviewer 1 also noted the lack of quantitative data in the paper. The authors introduced pre-factors to simplify the parameters that characterize phonon scattering by various defects, except for nanoparticles, in order to avoid unreliable physical parameters from TEM images, such as dislocation density (ND) and number density of nano-precipitates (NP), as noted in the supplementary information. This results in an excellent evaluation, as shown in Fig. 3. At the same time, however, the scattering parameters, such as the scattering power, cross-section (or diameter) and mean-free path of individual defects, have been disregarded as if they were thrown into a black box. These parameters are the most important indexes from a physical viewpoint and for the future development of thermoelectric materials.
The authors replied that "SPS-processed bulk samples usually exhibit a non-equilibrium microstructure." (The acronym SPS requires the full notation.) Does this mean that the density of induced defects, including dislocations, depends on the sample preparation procedure, such as temperature, strain and stress? For instance, helical dislocations that are formed by absorbing point defects through screw dislocations are easily annihilated during annealing. From a reproducibility

standpoint, as Reviewer 3 also noted, the reported results lack generality or universality for future research as defect densities depend heavily on the preparation procedure.
While this paper presents valuable results in the field of thermos-electrics through the manipulation of multidimensional defects and doping, I think that their research strategy would be more appropriate for a practice-oriented journal, unless the information on the physical characteristics of individual defects is provided.

Reviewer #3

(Remarks to the Author)
In the revised manuscript their claims are understandable with additional information and explanation. The authors tried to understand the mechanisms by combining transport properties and microscopic analysis. The discovery does not have a huge impact on the field; however, the data itself is original and worth reporting. I agree to accept this as the publication.

Reviewer #4

(Remarks to the Author)
All my comments have been properly addressed. I believe this manuscript is now ready to be accepted for publishing.

Reviewer #5

(Remarks to the Author)
I co-reviewed this manuscript with one of the reviewers who provided the listed reports. This is part of the Nature Communications initiative to facilitate training in peer review and to provide appropriate recognition for Early Career Researchers who co-review manuscripts.

Version 2:

Reviewer comments:

Reviewer #2

(Remarks to the Author)
I have reviewed the revised manuscript and supplementary materials, as well as the authors' responses to the previous comments regarding the physical characteristics of individual defects. The authors responded appropriately o my questions and provided the necessary information, primarily in the supplementary materials. I am currently inclined to give the paper a positive review, provided that the dislocation densities are described explicitly in the manuscript, possibly around Figs. 1 or 2, rather than in the supplementary materials. The numerical magnitudes are key information in the present research, given that the authors claim success in manipulating P-N stress to introduce an extremely high density of dislocations by the severe deformation. The lack of notation for the density is rather strange.

The following is an optional extra, not necessary to act. I am interested in whether the thermoelectric properties of such a high-energy or high-entropy state can be maintained on the TE timescale under practical heat conditions.

Version 3:

Reviewer comments:

Reviewer #2

(Remarks to the Author)
I am satisfied with your responses and the revised manuscript, which perfectly addressed my comments. I am now happy to support the publication of your paper in this journal. I appreciate your thoughtful responses to my comments and wish your further success in exploring materials in various fields including TE.

Open Access This Peer Review File is licensed under a Creative Commons Attribution 4.0 International License, which permits use, sharing, adaptation, distribution and reproduction in any medium or format, as long as you give appropriate credit to the original author(s) and the source, provide a link to the Creative Commons license, and indicate if changes were made.
In cases where reviewers are anonymous, credit should be given to 'Anonymous Referee' and the source.
The images or other third party material in this Peer Review File are included in the article's Creative Commons license, unless indicated otherwise in a credit line to the material. If material is not included in the article's Creative Commons license and your intended use is not permitted by statutory regulation or exceeds the permitted use, you will need to obtain permission directly from the copyright holder.
To view a copy of this license, visit https://creativecommons.org/licenses/by/4.0/

Dear reviewers,

Thank you very much for your thorough review of our manuscript entitled "**Manipulating Peierls-Nabarro stress to realize extraordinary thermoelectric performance in n-type PbTe**" (Manuscript ID: NCOMMS-26-005960A). We sincerely appreciate your positive evaluation of our manuscript, describing it as a "***compelling defect-engineering strategy***" (Reviewer #1), providing "***thorough results [that] are expected to contribute significantly***" (Reviewer #2), containing data that is "***worth reporting***" (Reviewer #3), and being "***of great importance to the materials science community***" (Reviewer #4).

We also value your insightful comments and constructive suggestions, particularly those regarding the quantification of Sb_6O_{13} nanophases, the mechanistic role of dislocations, the rationalization of experimental compositions, and the thermal stability of the nanostructures. These comments are extremely valuable for improving the clarity, rigor, and overall quality of the manuscript.

A detailed, point-by-point response to all comments is provided below, and the manuscript has been revised accordingly. For details please see "The Revised Manuscript with Changes Marked", in which main revisions are highlighted in blue. We hope that this updated version addresses your concerns and is now suitable for publication in ***Nature Communications***.

Yours sincerely,

Prof. Dr. Jian Zhang.

On behalf of all co-authors

E-mail: zhangjian@issp.ac.cn

Reply to the reviewers' comments

Reply to reviewer 1

Comments: This study presents a compelling defect-engineering strategy to enhance the thermoelectric performance of PbTe by coupling lattice softening with electronic structure modification. By manipulating Peierls-Nabarro stress through solute alloying, the authors induce a high density of dislocations and subgrain boundaries that effectively scatter phonons, while the in-situ formation of Sb_6O_{13} nanoinclusions introduces additional multiscale phonon scattering. Simultaneously, Sb doping increases the electronic density of states, leading to an improved power factor. The resulting combination of ultralow lattice thermal conductivity and high ZT (~2.2) highlights the effectiveness of integrating defect mechanics with band engineering and provides valuable insight into designing high-performance thermoelectric materials beyond conventional alloying approaches. The article can be accepted after major revision. I suggest the authors address the following comments:

Thank you for your positive comments on our manuscript. Following is our response to your comments.

Major comments

1. Transition between SEM-EDS and TEM observations

There is insufficient transition in the discussion from SEM-EDS results to TEM observations regarding the appearance of Sb_6O_{13} nanophases (nanoprecipitates). It would be helpful to explicitly clarify that, due to resolution limitations, SEM-EDS indicates a uniform elemental distribution in the 1.8 wt.% Sb sample (Fig. S4), whereas higher-resolution TEM clearly reveals the presence of Sb_6O_{13} nanophases. This transition from apparent compositional uniformity to the PbTe/Sb_6O_{13} two-phase microstructure is also not clearly described in the discussion of Fig. 1g.

Response: Thank you for this constructive suggestion. We agree that the transition from micrometer scale SEM observations to nanometer scale TEM analysis requires more explicit clarification. In the revised manuscript, we have clarified that the apparent uniform elemental distribution in the SEM-EDS results (**Supplementary Fig. 4**) is attributed to the spatial resolution limitations of the technique. While the SEM results suggest macroscopic homogeneity, the technique cannot resolve individual Sb_6O_{13} nanophases because their diameters range from ~20 nm to ~80 nm. Therefore, high resolution TEM is indispensable for revealing the actual PbTe/Sb_6O_{13} two phase microstructure. We have updated the discussion of **Fig. 1g** to bridge these two scales of observation.

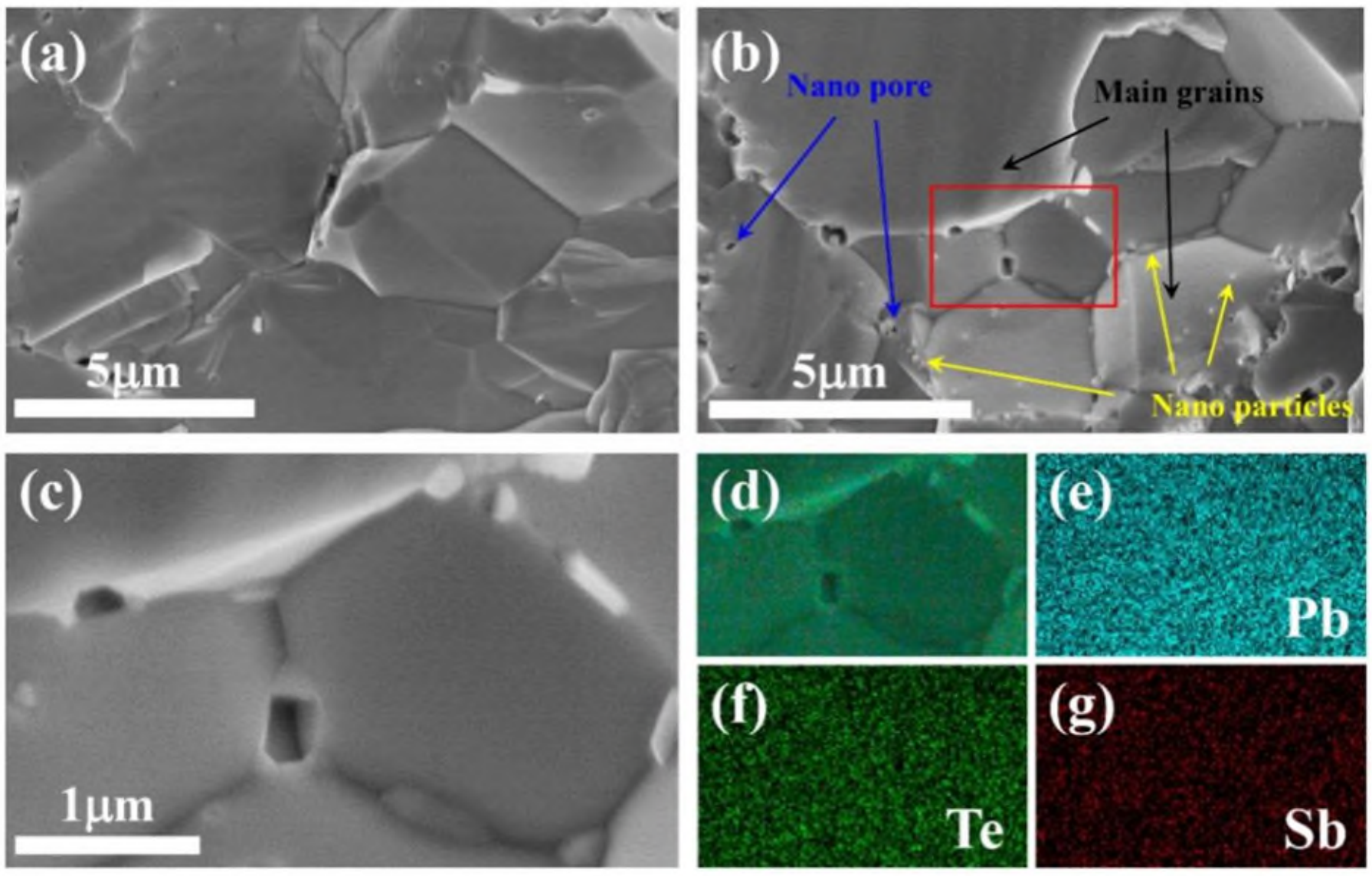

Supplementary Fig. 4 | FE-SEM images of the fracture surface of **(a)** PbTe and **(b)** PbTe-1.5wt.%TeO_2-1.8wt.%Sb sample; **(c)** magnified image of selected rectangle area in **(b)**; **(d-g)** EDS analysis of the elements Pb, Te and Sb.

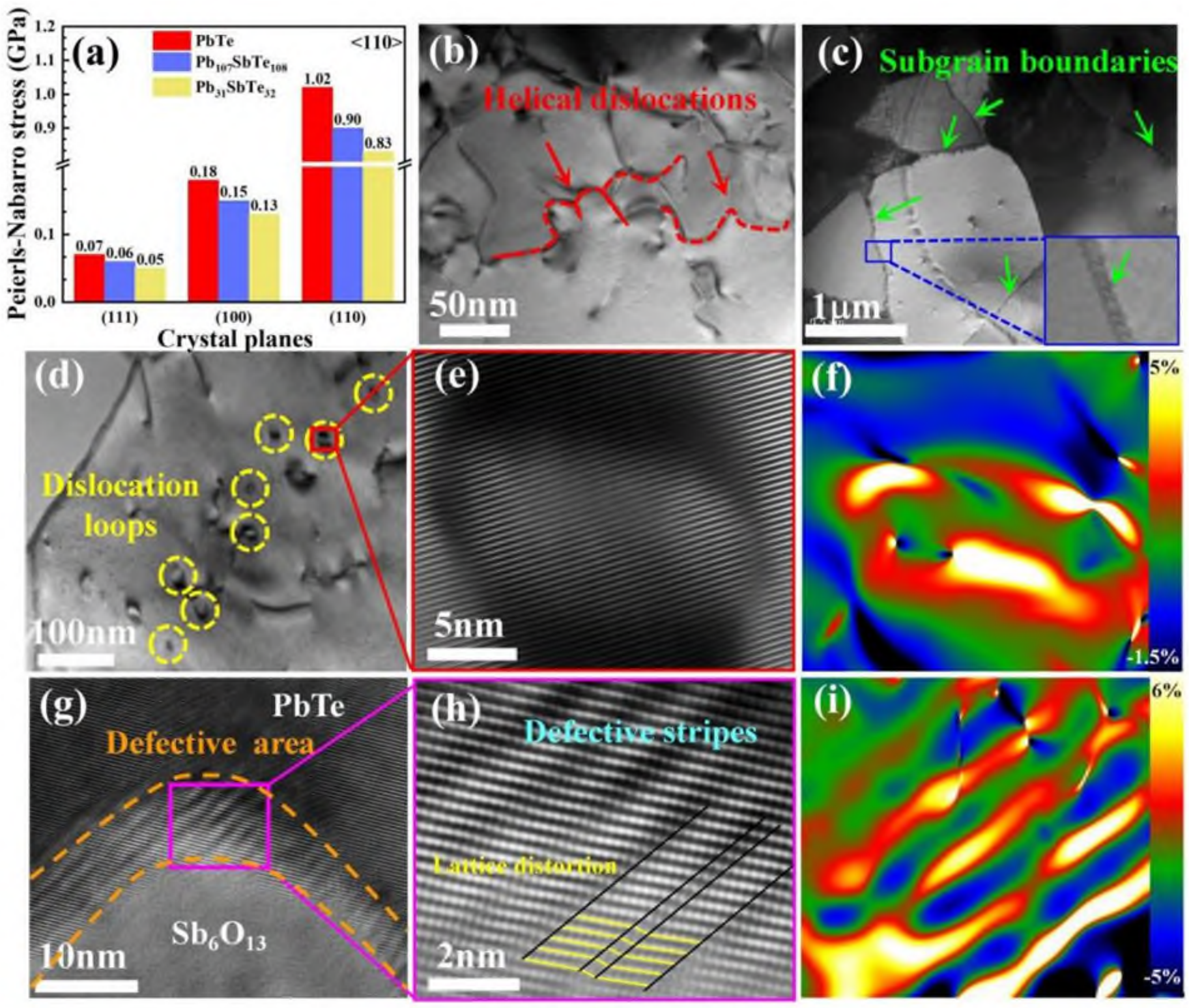

Fig. 1 | Peierls-Nabarro Stress and Microstructure characterization. (a) Low-index planes Peierls-Nabarro stress of PbTe and Sb substituted PbTe samples. **(b-d)** Low-magnification TEM images of helical dislocations, subgrain boundaries and dislocation loops in PbTe-1.5wt.%TeO_2-1.8wt.%Sb composite sample; the HRTEM images of dislocation loop **(e)**; **(f)** GPA strain analysis of **(e)**; the HRTEM images of defective area **(g)** and lattice distortion **(h)**; **(i)** GPA strain analysis of **(h)**.

2. Quantification of Sb_6O_{13} nanophase abundance

There is a lack of quantitative data on the abundance of Sb_6O_{13} nanophases. Figures 2a and 2b suggest a periodic microstructure of Sb_6O_{13} nanophases within a PbTe matrix grain; however, most nanoprecipitates appear to be located near PbTe/PbTe grain boundaries, with only a limited number observed within grain interiors. Quantification of precipitate density and spatial distribution would provide a stronger link to the calculated spectral lattice thermal conductivity (Ks).

Additionally, the observed morphology suggests that Sb_6O_{13} nanoprecipitates may form a Widmanstätten-like pattern within the PbTe matrix, with rows seemingly aligned parallel to the {100} direction of PbTe. This hypothesis should be verified or discussed more carefully.

In lines 143–144 (Supplementary Information), the authors state that prefactors were introduced to avoid uncertainties arising from TEM-based precipitate and dislocation density calculations.

Although Table S3 lists Np values, it is not clear whether these values are derived directly from TEM image analysis or assumed for modeling purposes. This should be clarified.

Response: Thank you for your detailed and insightful evaluation of our microstructural analysis and theoretical modeling. We address each of these excellent points below:

(1) We agree that bridging the quantitative microstructural data with the spectral lattice thermal conductivity (Ks) is crucial. Regarding the abundance of the nanophases, we have provided macroscopic quantitative data via XRD Rietveld refinement. As shown in **Supplementary Table 2** , the bulk mass fraction of Sb_6O_{13} increases systematically from 0 to 1.3 wt.% with increasing Sb content. This bulk measurement provides a more statistically representative quantification of the precipitate abundance than local TEM counting. Regarding the spatial distribution, the Sb_6O_{13} nanophases are primarily concentrated within the PbTe grain interiors as oriented Widmanstätten-like structures, although a smaller portion is also distributed at the grain boundaries (**Fig. R1**). The Fast Fourier Transform (FFT) analysis in **Fig. R2a** and the atomic-resolution images in **Fig. R2b** and **Fig. R2c** confirm a coherent or semi-coherent orientation relationship between the precipitates and matrix. Such specific crystallographic alignments typically occur during solid-state precipitation inside the grains.

Supplementary Table 2. Relative Density (d_r), the mass fraction (f) of impurity phase Sb_6O_{13} obtained from Rietveld refinement analysis and the factor of GSAS Rietveld Refinement (R_w) for PbTe-1.5wt.%TeO_2-$x$wt.%Sb samples.

Nominal composition	d_r (%)	f (wt.%)	R_w(%)
**PbTe**	98.70	0	6.631
**PbTe-1.5wt.%TeO_2-0.36wt.%Sb**	96.87	0.2	6.178
**PbTe-1.5wt.%TeO_2-0.72wt.%Sb**	96.07	0.5	5.790
**PbTe-1.5wt.%TeO_2-1.08wt.%Sb**	95.78	0.7	5.666
**PbTe-1.5wt.%TeO_2-1.44wt.%Sb**	96.51	0.9	6.138
**PbTe-1.5wt.%TeO_2-1.80wt.%Sb**	95.95	1.2	5.967
**PbTe-1.5wt.%TeO_2-2.16wt.%Sb**	98.64	1.3	6.015

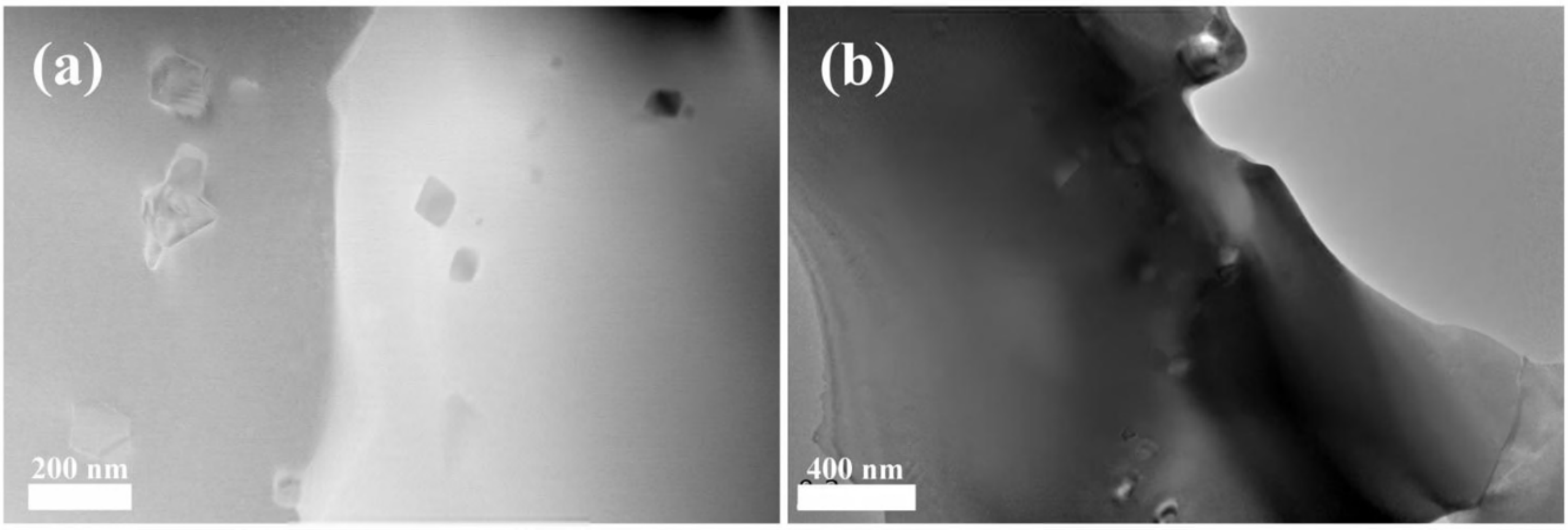

Fig. R1 | Representative large area TEM micrographs showing the spatial distribution of Sb_6O_{13} nanoprecipitates within the PbTe matrix.

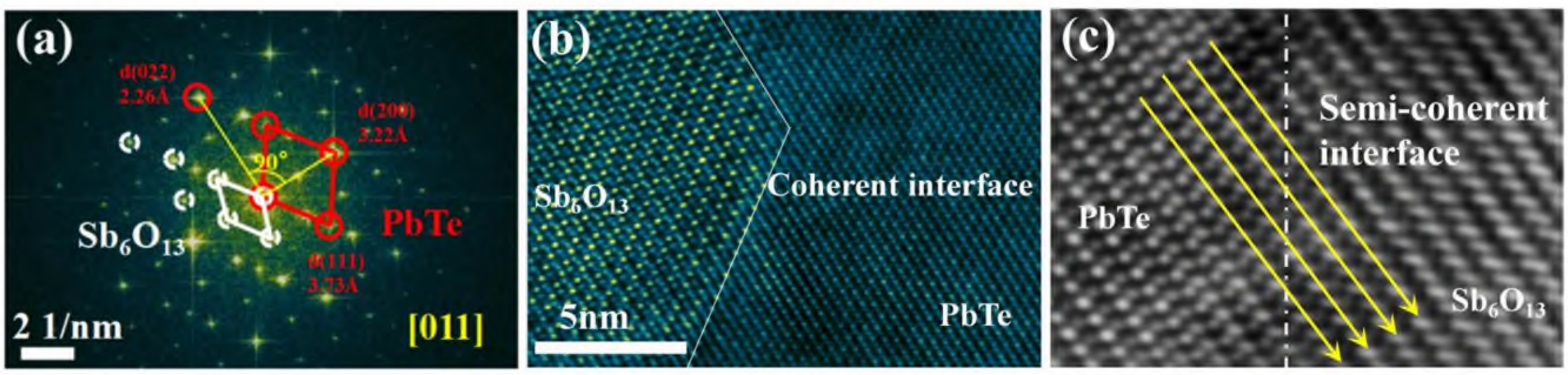

Fig. R2 | Microstructures of nanoprecipitate in PbTe-1.5wt.%TeO_2-1.8wt.%Sb sample. (a) FFT image; (b) atomic-resolution interface image, where the coherent interface relations are shown; (c) atomic-resolution interface image, where the semi-coherent interface relations are shown.

(2) Based on the rigorously indexed FFT pattern of the PbTe matrix recorded along the [011] zone axis in **Fig. R2a**, we have carefully verified the orientation relationship using the standard matrix reflections with d(200) = 3.20 Å, d(022) = 2.26 Å, and d(111) = 3.73 Å. Consistent with your analysis, the real-space morphology indeed confirms a Widmanstätten-like growth pattern, and our analysis reveals that the precipitates are aligned with the {111} habit planes of PbTe. As illustrated in the FFT image, the characteristic reflections of the Sb_6O_{13} phase (indicated by white circles) do not lie along the radial direction of the PbTe (200) spot; instead, they exhibit a clear coaxial alignment along the diagonal vector corresponding to the PbTe {111} reflection (d = 3.73 Å}). This precise vector alignment in reciprocal space demonstrates a strict cube-on-cube orientation relationship where the Sb_6O_{13} phase preferentially precipitates along the most close-packed {111} planes of the matrix to minimize interfacial strain energy. Consequently, we have revised the manuscript to accurately describe this {111}-oriented Widmanstätten-like morphology, ensuring that our structural discussion is fully consistent with the provided crystallographic evidence.

(3) We would like to clarify that the N_p values listed in **Supplementary Table 3** are not assumed for modeling but are calculated by integrating results from both X-ray diffraction and transmission electron microscopy. Specifically, the mass fraction of the Sb_6O_{13} phase is first determined through Rietveld refinement of the XRD patterns (**Supplementary Table 2**). This mass fraction is then converted into a volume fraction by utilizing the theoretical densities of the PbTe matrix and the Sb_6O_{13} precipitates. Subsequently, the average volume of individual Sb_6O_{13} nanoinclusions is statistically obtained from multiple TEM images. By dividing the total volume fraction by the average volume per precipitate, we derive the N_p values used in the Debye-Callaway model. The prefactors mentioned in Supplementary Information Section 6 are introduced to account for potential statistical variations such as local distribution inhomogeneities. We have updated the Supplementary Information to include this detailed explanation.

3. Reduction in effective number of Sb_6O_{13} nanophases

In lines 206–207 of the main text, the authors state that there is a reduction in the effective number of Sb_6O_{13} nanophases; however, no clear quantification or supporting analysis is provided. Please include

quantitative evidence or clarify the basis for this statement.

Response: Thank you for this insightful comment. The statement regarding the "reduction in the effective number of Sb_6O_{13} nanophases" for the x = 2.16 sample is based on the quantitative analysis of the nanostructure parameters used in the Debye-Callaway model (**Supplementary Table 3**). Specifically, as the Sb content x increases from 1.80 to 2.16, the Sb_6O_{13} nanophases undergo coarsening, with the average radius R increasing from ~30 nm to ~60 nm. Consequently, the number density N_P of these nanoprecipitates significantly decreases from 6.5×10^{21} m^{-3} for x = 1.80 to 1.5×10^{21} m^{-3} for x = 2.16. This sharp reduction in the number density of scattering centers explains the weakened phonon scattering and the subsequent increase in lattice thermal conductivity observed in the x = 2.16 sample. We have added these quantitative details to the revised manuscript to clarify this point.

Supplementary Table 3. Parameters used in the Debye-Callaway calculation for PbTe-1.5wt.%TeO_2-$x$wt.%Sb samples.

Parameters	x=0	x=0.36	x=0.72	x=1.08	x=1.44	x=1.80	x=2.16
**A_1 ($10^{-17}sK^{-1}$)**	1.04	1.07	1.15	1.28	1.35	1.40	1.45
**A_2 ($10^{-41}s^3$)**	1.1	1.2	2.1	4.19	6.03	8.80	8.90
**A_3 (10^{-3})**	--	0.12	0.28	2.42	6.43	12	5.20
**A_4 (10^{-28})**	--	0.27	0.53	1.35	1.9	2.7	1.2
**L (10^2 nm)**	30	25	20	15	10	7	9
**N_P (10^{21} m^{-3})**	--	0.16	0.82	2.75	4.3	6.5	1.5

4. Phonon scattering parameters

For both Sb_6O_{13} nanophase phonon scattering and dislocation scattering mechanisms, it would significantly strengthen the manuscript if the authors provided more detailed quantitative information on defect densities and a more comprehensive characterization of Sb_6O_{13} spatial distribution.

Response: We appreciate your suggestion to provide more detailed quantitative information regarding defect densities and the spatial distribution of Sb_6O_{13} nanophases. In the revised manuscript and Supplementary Information, we have included a comprehensive quantitative analysis to support our scattering mechanisms. For nanophase scattering, we utilized the statistical data from XRD Rietveld

refinement and multiple TEM images to determine the number density N_P of Sb_6O_{13} precipitates for PbTe-1.5wt.%TeO_2-$x$wt.%Sb samples (**Supplementary Table 3**). The spatial distribution was further characterized by large-area TEM imaging, confirming that these nanoprecipitates are not only distributed at grain boundaries but also densely populated within the PbTe grain interiors as oriented Widmanstätten-like structures, maximizing the phonon-boundary scattering area, as shown in **Fig. R1**. For dislocation scattering, As noted in the Supplementary Information, we introduced the prefactors A3 and A4 to mitigate potential uncertainties associated with calculating absolute dislocation densities solely from TEM images. Within the framework of the Debye-Callaway model, the scattering rates for dislocation strain fields and dislocation cores are expressed by **Supplementary Equations 15 and 19**. These equations explicitly demonstrate that the parameters A3 and A4 are directly proportional to the dislocation density N_D. Consequently, the values of A3 and A4 listed in **Supplementary Table 3** serve as a robust semi quantitative measure for monitoring the evolution of dislocation density across different samples. The significant increase in these prefactors as the Sb content x rises from 0 to 1.80 provides clear quantitative evidence of the enhanced dislocation density, which is in excellent agreement with our microstructural observations. Notably, for the sample with $x = 2.16$, the coarsening of the secondary nanophases reduces the total interfacial area and the density of geometric connection points for dislocations. Simultaneously, the increased inter particle spacing weakens the pinning and stabilization effects on the dislocation network. This structural evolution leads to a macroscopic reduction in dislocation density, which is quantitatively reflected by the decreased values of the prefactors A3 and A4 in our model.

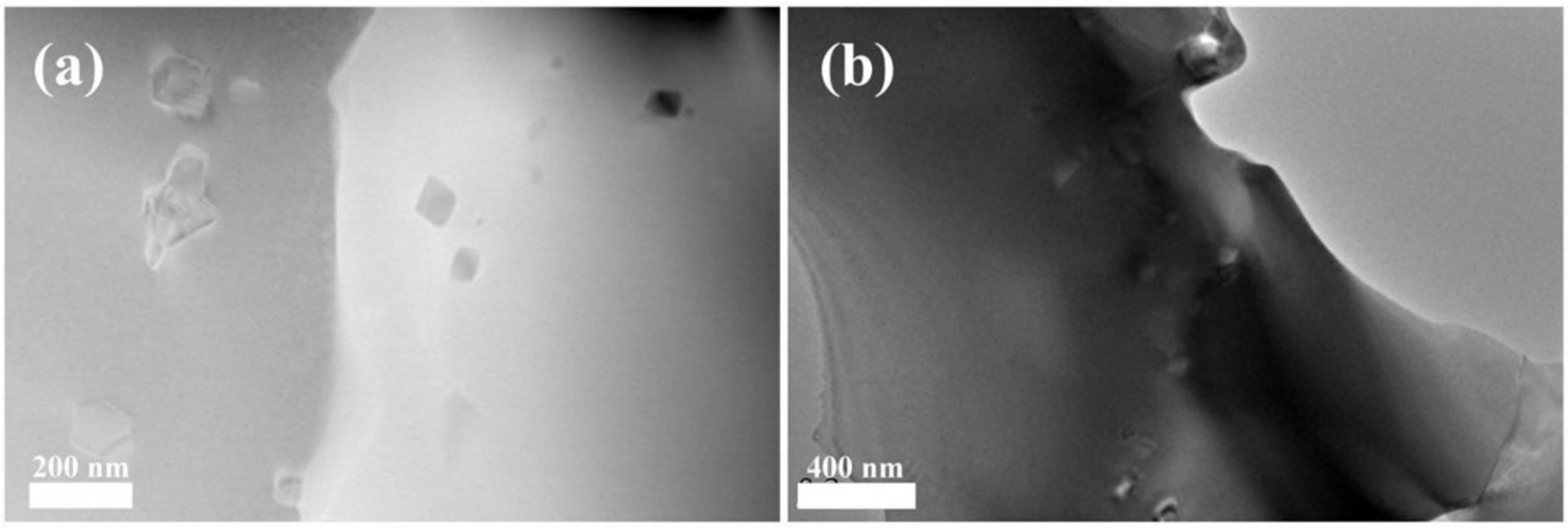

Fig. R1 | Representative large area TEM micrographs showing the spatial distribution of Sb_6O_{13} nanoprecipitates within the PbTe matrix.

The relaxation time corresponds to dislocation strain fields and dislocation cores phonon scattering:

$$\tau_{\mathrm{DS}}^{-1} = 0.6B_{\mathrm{D}}^{2}N_{\mathrm{D}}\gamma^{2}\omega\left\{\frac{1}{2}+\frac{1}{24}\left(\frac{1-2r}{1-r}\right)^{2}\left[1+\sqrt{2}\left(\frac{v_{\mathrm{L}}}{v_{\mathrm{T}}}\right)\right]\right\} + N_{\mathrm{D}}\frac{V^{\frac{4}{3}}}{v_{\mathrm{S}}^{2}}\omega^{3} \quad (15)$$

The relaxation time corresponds to dislocation strain fields and dislocation cores phonon scattering:

$$\tau_{DS}^{-1} = A_3\omega + A_4\omega^3 \quad (19)$$

Supplementary Table 3. Parameters used in the Debye-Callaway calculation for PbTe-1.5wt.%TeO_2-*x*wt.%Sb samples.

Parameters	*x*=0	*x*=0.36	*x*=0.72	*x*=1.08	*x*=1.44	*x*=1.80	*x*=2.16
**A_1 (10^{-17}sK^{-1})**	1.04	1.07	1.15	1.28	1.35	1.40	1.45
**A_2 (10^{-41}s^{3})**	1.1	1.2	2.1	4.19	6.03	8.80	8.90
**A_3 (10^{-3})**	--	0.12	0.28	2.42	6.43	12	5.20
**A_4 (10^{-28})**	--	0.27	0.53	1.35	1.9	2.7	1.2
**L (10^{2} nm)**	30	25	20	15	10	7	9
**N_P (10^{21} m^{-3})**	--	0.16	0.82	2.75	4.3	6.5	1.5

Minor comments and typographical corrections

1. Please clarify in the Fig. S6 caption that all TEM and GPA analyses were performed on the PbTe matrix.

Response: We have revised the caption of Supplementary Fig. 6 as suggested. We have now explicitly clarified that all TEM and GPA analyses presented in this figure were performed on the PbTe matrix to avoid any potential ambiguity regarding the analyzed phase.

2. Line 118 (main text): revise to "Pb vacancies."

Response: We have revised the text in Line 118 as suggested, replacing the original term with "Pb vacancies" to accurately describe the point defects in the PbTe lattice.

3. Line 154 (main text): revise to "are close."

Response:We have revised the text in Line 154 by replacing the original phrase with "are close" as suggested, ensuring the sentence is concise and grammatically standard.

4. Line 252 (main text): revise to "effective mass."

Response:We have revised the term in Line 252 to "effective mass" as suggested.

5. Line 152 (main text): the text states that only a coherent PbTe/Sb6O13 interface is present; however, Fig. 2d shows a coherent interface, whereas Fig. 2h clearly shows a semi-coherent PbTe/Sb_6O_{13} interface, as also indicated by the figure labels. Please revise the text to reflect the presence of both interface types.

Response: We sincerely apologize for the imprecise description in the original manuscript. We agree with you that both coherent and semi-coherent interfaces coexist between the PbTe matrix and Sb_6O_{13} nanoprecipitates, as accurately demonstrated in Fig. 2d and 2h, respectively. The coherent interface (Fig. 2d) typically occurs at the early stages of precipitation or along specific low-index planes with minimal lattice mismatch, while the semi-coherent interface (Fig. 2h) forms as the precipitates grow or rotate, necessitating the introduction of interfacial dislocations (as shown in the GPA and HRTEM analysis) to accommodate the lattice strain. We have revised Line 152 and the surrounding discussion to accurately reflect the presence of both interface types and their role in phonon scattering.

Reply to reviewer 2

Comments: This paper presents a method for producing high-performance thermoelectric (TE) lead telluride (PbTe)-based alloys by controlling multidimensional defects. Various dimensional defects scatter in ways that conflict with thermal and electrical conductivity, creating an inevitable challenge in achieving an optimized balance of them. Currently, many groups around the world are working to improve the performance of TE energy-conversion PbTe alloys, especially n-type ones. The wide-ranging, thorough results reported in the paper are expected to contribute significantly to the realization of excellent n-type PbTe alloys. While this paper does not present new physics, it does provide optimized technological parameters for TE processes. Indeed, the authors emphasize achieving the best possible results among the materials. This review was conducted to understand the mechanisms underlying in the processes. In particular, the contribution of dislocations is examined in light of the paper's title. The authors theoretically estimated the reduction of the Peierls-Nabarro (PN) stress in the PbTe alloys to introduce many dislocations. These alloys are known to be narrow-gap semiconductors, which are typically soft materials, even at room temperature (Acta Mater. 61, 294 (203) by Kamimura

et al.). The PN stress is defined at 0 K and decreases at elevated temperatures, which is where the present samples were sintered. In this case, the dislocation densities are supposed to be similar in the alloys and to depend minimally on the composition. Thus, their contribution to the thermal conductivities through the phonon scattering is questionable. Instead, such alloying leads to mechanical solution hardening. To refuse this claim, the authors could demonstrate the density of dislocations in the samples with different compositions. This paper does not mention the effects of dislocations on electrical conductivity. In this research, the alloys were prepared by sintering, raising the question of the size of the grains in the prepared poly-crystals. According to G.A. Slack (J. Appl. Phys. 70,12694 (1991), the barrier effects of grain boundaries against thermal conductivity only appear when the grain size is smaller than 1 μm. Can the reported TE properties be understood as resulting from various scatterings caused by the multidimensional defects introduced in the alloys? These phenomena are known as the alloying effect of a high-entropy system. Of course, clarifying how these respective defects contribute to the relevant scatterings is important both academically and practically.

Response: We sincerely value your profound assessment of our work. Your expertise in the mechanical and thermal properties of PbTe alloys is evident, and the questions you raised regarding the Peierls-Nabarro (P-N) stress and the role of dislocations are essential for clarifying the underlying physics of our "multidimensional defect" strategy. Our responses to your comments are shown below.

(1) **On P-N Stress and Temperature**: While we agree with your observation that PbTe is a relatively soft material and its P-N stress is temperature-dependent, our study focuses on the residual dislocation density after the complete thermal-mechanical cycle of sintering and cooling. It should be noted that SPS-processed bulk samples usually exhibit a non-equilibrium microstructure. According to your suggestions, the TEM micrographs of dislocations in PbTe-1.5wt.%TeO_2-0.36wt.%Sb and PbTe-1.5wt.%TeO_2-1.08wt.%Sb samples have been added in **Fig. R3**. We can see that the number of dislocations in PbTe-1.5wt.%TeO_2-1.80wt.%Sb sample is higher than in PbTe-1.5wt.%TeO_2-0.36wt.%Sb and PbTe-1.5wt.%TeO_2-1.08wt.%Sb samples. This suggests that solute-induced lattice softening does not merely lower the "barrier" for a moment, but facilitates a massive proliferation of dislocations during the rapid sintering process, which are then effectively "pinned" or "locked" by the nanostructures and solute atoms during cooling. Therefore, the manipulation of P-N stress is the kinetic trigger that enables a steady-state high density of defects that would otherwise not be preserved.

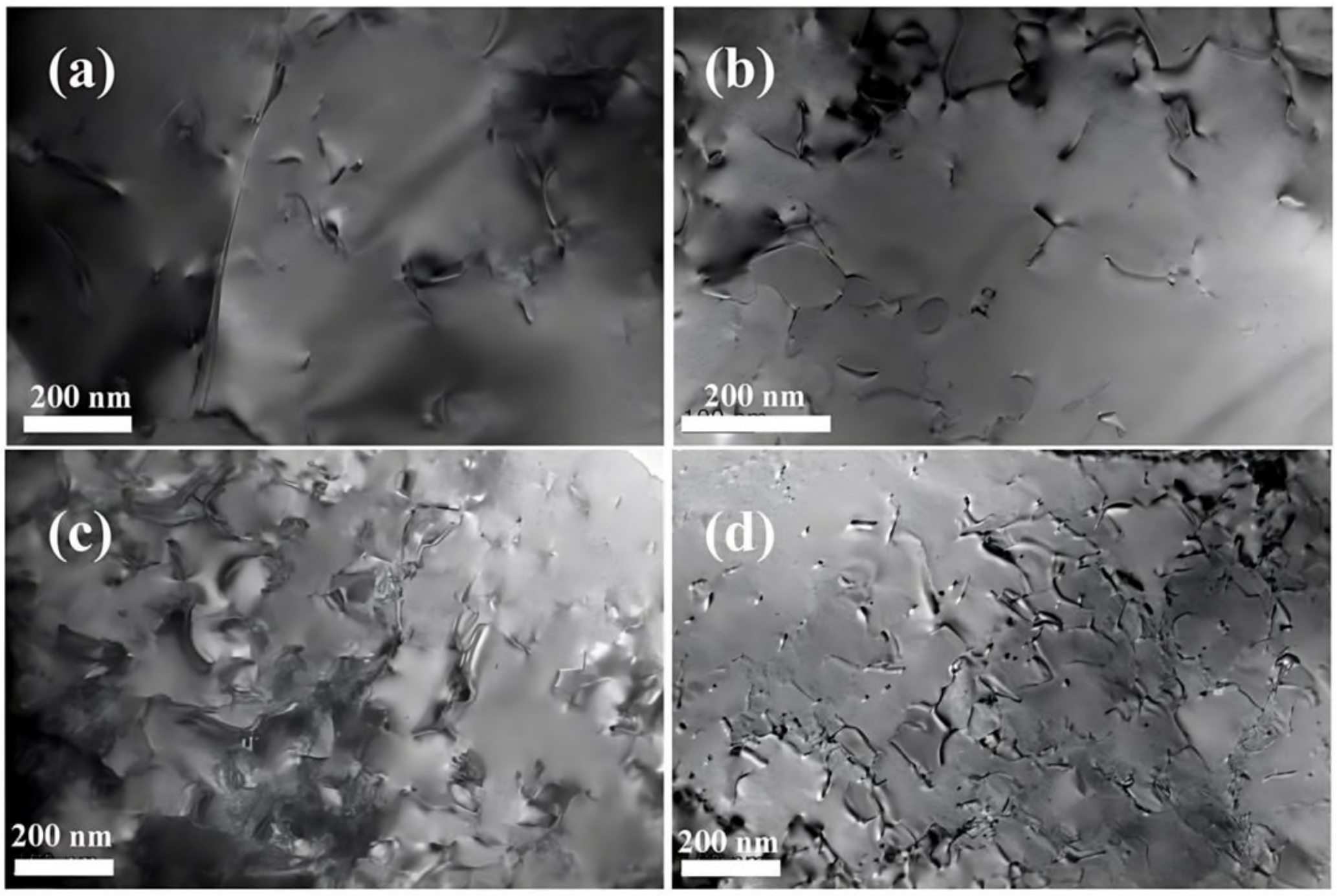

Fig. R3 | TEM micrographs of dislocations in PbTe-1.5wt.%TeO_2-0.36wt.%Sb (a), PbTe-1.5wt.%TeO_2-1.08wt.%Sb (b) and PbTe-1.5wt.%TeO_2-1.80wt.%Sb (c-d) samples.

(2) **Effects on Electrical Transport:** We appreciate your focus on the potential trade-off between thermal and electrical transport. To clarify your concerns, the temperature-dependent carrier mobility and carrier concentration are summarized in **Supplementary Fig. 11**, which can clearly demonstrate our relevant results. The carrier mobility for the optimized x = 1.80 sample does not exhibit a significant reduction compared to other samples despite the high density of multidimensional defects. This experimental evidence is crucial for two reasons. First, it directly addresses your point regarding electrical transport. According to the Dexter-Seitz model, the strain fields from dislocations are highly effective at scattering phonons while remaining relatively transparent to carriers in this n-type PbTe system. This allows the material to maintain high electrical performance while suppressing heat transport. Second, this high mobility strongly refutes the claim that the thermal conductivity reduction is purely an alloying effect. In a typical heavily alloyed system, the intense mass and size fluctuations would cause strong alloy scattering, which inevitably leads to a dramatic decline in carrier mobility. The fact that our mobility remains largely uncompromised indicates that the scattering centers in our samples are frequency selective. These multidimensional defects target mid to long wavelength phonons effectively but do not significantly hinder carrier transport. Therefore, the ultra-low lattice

thermal conductivity results from the synergistic scattering of specific dislocations and nanophases instead of a general alloying effect.

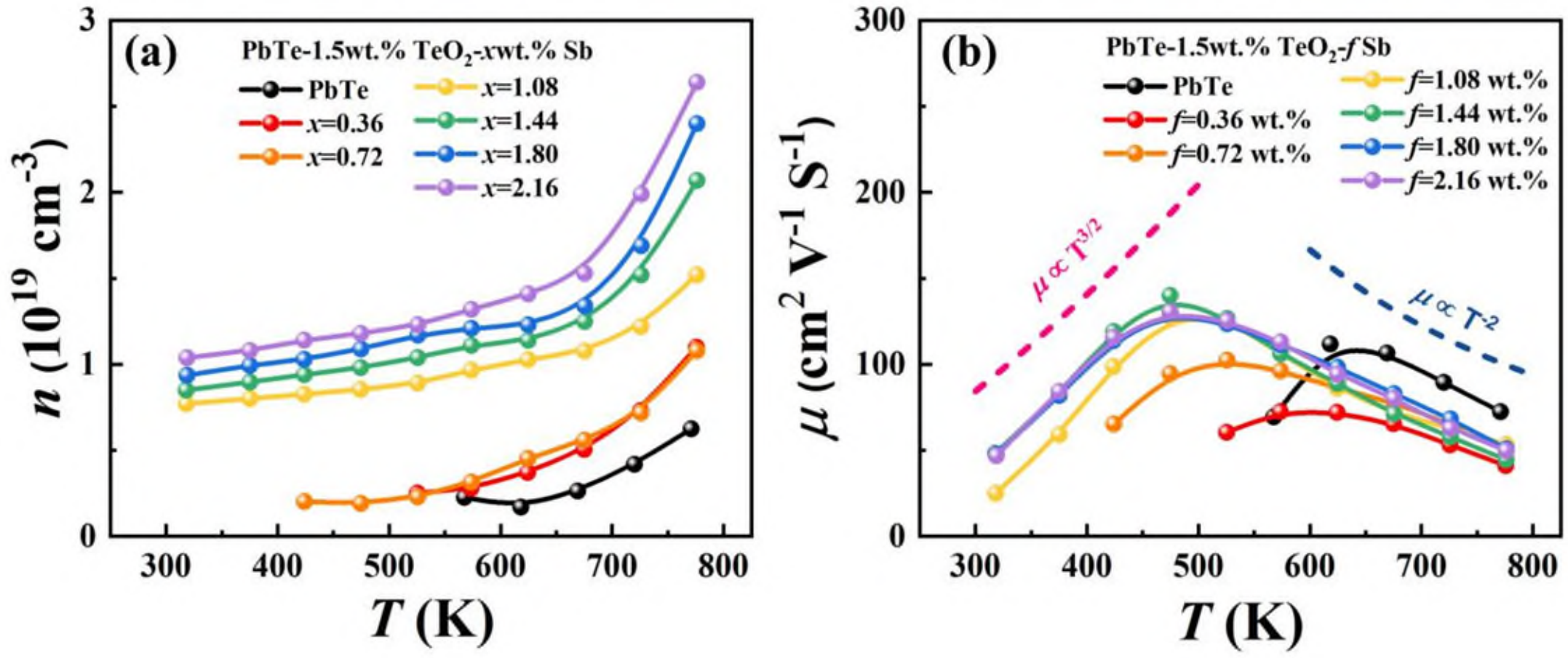

Supplementary Fig. 11 | Temperature dependent **(a)** electron concentration and **(b)** electron mobility for PbTe-1.5wt.%TeO_2-*x*wt.%Sb samples.

(3) **Grain Boundary Effects:** We concur with your reference to Slack's theory. Since our grain sizes are in the 2-10μm range, we agree that the contribution of macroscopic grain boundaries to phonon scattering is minimal. This is precisely why we emphasize intra grain multidimensional defects. The "multidimensional" strategy we describe focuses on defects within the grains, specifically, the high density helical dislocations and nanoprecipitates, whose characteristic spacing is significantly shorter than the phonon mean free path.We have clarified this in the text to ensure our focus on intra-grain engineering is not confused with traditional grain-boundary engineering.

The following technological point should be considered:

As noted above, the paper thoroughly reports that the authors obtained the top ZT record in the material system. However, the descriptions are overall detailed, making it difficult to understand the results without the supplementary materials, which include important basic data, such as how the samples were prepared. This may be an attempt to emphasize the accomplishment of obtaining top data. This results in the diffusion of data between the text and supplementary materials. Describing only the most important data, such as TEM observations, thermal and electrical conductivity, and ZT for the specific alloy with the top data in the text, would be much effective. The rest can be shown in the supplementary materials. Additionally, reconsidering the overall text, including figures and English descriptions, is

necessary for successful publication.

Response: Thank you for your suggestion. We sincerely appreciate your constructive suggestions regarding the organization and clarity of our manuscript. We agree that the current presentation can be streamlined to create a more focused and impactful narrative. First, we have restructured the main text to follow your recommendation. We now focus the primary discussion on the champion x = 1.80 sample. This includes its TEM observations, thermal and electrical transport properties, and the record ZT performance. The data for other compositions and intermediate optimization steps have been moved to the Supplementary Information. This modification ensures that the core physical mechanisms and our primary accomplishments are highlighted without the distraction of excessive secondary data.Second, we have moved the essential details of the sample preparation and experimental methods from the Supplementary Information back into the main text. This change ensures that the manuscript is more self-contained. It allows readers to understand the fundamental basis of our work without the need for constant cross-referencing. Finally, we have performed a comprehensive revision of the entire manuscript. This includes a thorough linguistic polish of the English descriptions to ensure professional clarity. We have also refined the figures to improve their visual impact and technical precision. We believe these changes significantly enhance the overall quality and readability of our work for the *Nature Communications* audience.

Reply to reviewer 3

Comments: PbTe based compounds has been recognized as one of the best thermoelectric materials, and microstructure modification is known as the effective way to improve the performance of this system. Authors reported the change in transport properties with different x content in PbTe-1.5wt.%TeO_2-xwt.%Sb, combined with detailed microstructure analysis. The reported data is worth reporting, however some questions about transport analysis need to be answered to clarify their claims. Therefore, I recommend the present work for publication only after addressing the following queries.

Thank you for your positive comments on our manuscript. Following is our response to your comments.

1. The introduction lacks sufficient discussion of the roles of dislocations and softening. The rationale behind the chosen experimental compositions (PbTe-1.5wt.%TeO_2-xwt.%Sb) is not clearly explained.

Please elaborate on how these factors influenced the design of the study.

Response: Thank you for this suggestion. We sincerely appreciate your constructive feedback on the theoretical rationale and material design. We agree that the introduction requires clearer articulation of these mechanisms. We have now expanded the introduction to explicitly detail the roles of lattice softening and the specific logic behind our composition design. Regarding the role of softening and dislocations, our strategy relies on lowering the activation energy barrier for dislocation glide. The first principle calculations demonstrate that substituting Sb for Pb significantly reduces the Peierls-Nabarro stress for the (111)<110> slip system from 0.07 to 0.05 GPa. This solute induced lattice softening makes the lattice more susceptible to plastic deformation. When this softened lattice is coupled with the thermal stress introduced by endotaxial nanoinclusions, the generation and proliferation of high density dislocations are massively facilitated during the material processing. Regarding the rationale for the PbTe-1.5wt.%TeO_2-xwt.%Sb composition, the design involves a dual function strategy. The addition of TeO_2 is specifically designed to trigger an in situ displacement reaction with Sb ($13TeO_2 + 12Sb \rightarrow 2Sb_6O_{13} + 13Te$). This reaction generates the Sb_6O_{13} nanophases which provide the necessary thermal stress and act as phonon scattering centers. The variation of the Sb content x is crucial because Sb acts as both a reactant and a dopant. A portion of the Sb reacts with the fixed TeO_2 source, while the excess unreacted Sb substitutes Pb in the matrix. This substituting Sb is responsible for softening the lattice to induce dislocations and simultaneously optimizing the electron concentration and effective mass for enhanced electrical transport. We have modified the Introduction and the Results sections to explicitly reflect this comprehensive design logic.

2. Given that nanostructures, dislocations, grain boundaries, strain, and lattice defects are all present and potentially interacting, it is unclear whether the fittings shown in Figure S9 (Supplementary Fig. 10 in the revised manuscript) and Table S3 are free from arbitrariness. The authors should clarify which parts of the interpretation are experimentally confirmed and which parts are inferred or model dependent.

Response: Thank you for the comment regarding the potential arbitrariness in our lattice thermal conductivity fittings. We clarify that the fitting procedure is strictly constrained by experimental data to ensure physical validity and uniqueness. The interpretation is categorized into experimentally confirmed and model dependent parts. First, the experimentally confirmed parameters include the mass

fraction of Sb_6O_{13} determined by XRD Rietveld refinement and the average size of nanoprecipitates statistically obtained from TEM images. These data are used to fix the nanophase number density N_p in our model. The measured lattice thermal conductivity serves as the primary target for the fitting, while standard physical constants for PbTe are adopted from established literature. Second, the model dependent parts involve the dislocation scattering prefactors A3 and A4. To avoid arbitrariness, N_p is maintained as a constant based on experimental statistics, and A3 and A4 are only allowed to vary within physically reasonable orders of magnitude. The fact that the fitted trends of A3 and A4 across all samples correlate perfectly with our TEM observations of dislocation density evolution provides strong evidence for the reliability of our interpretation.

3. The simulation data appears to have the limited endorsement for the conclusion in their current form. Instead of relying on simulations to demonstrate high performance, it may be more convincing to fabricate and evaluate an actual module to claim the higher conversion efficiency.

Response: We completely agree with you that fabricating and evaluating an actual thermoelectric module is the ultimate goal to demonstrate the practical viability of any new material. We appreciate your constructive feedback on the presentation of our conversion efficiency data. To clarify the reliability of our COMSOL finite element simulation: The simulations were not based on idealized theoretical calculations. All input parameters used in the simulation, including the temperature dependent electrical conductivity, thermopower, and thermal conductivity, were taken directly from our experimental measurements of the optimized x = 1.80 sample. The purpose of this three dimensional simulation was to evaluate the theoretical performance limit of our material under realistic geometric and thermal flow conditions, thereby moving beyond oversimplified one dimensional analytical models. However, we fully acknowledge your point that finite element simulations represent an upper bound performance. In a real physical device, factors such as contact resistance at the metal semiconductor interfaces and thermal parasitics would inevitably lower the measured efficiency. To bridge this gap, fabricating high performance modules requires overcoming numerous independent engineering challenges. These include the precise control of contact resistance, matching suitable p-type partners with compatible thermal expansion coefficients, and ensuring interface stability. These engineering tasks are highly complex and beyond the scope of this present work which focuses on the fundamental physics of multidimensional defect scattering in bulk materials. To address your concerns, we have revised the manuscript to explicitly state that the simulated efficiency values represent a

theoretical projection of material potential.

4. Why is Sb content expressed in wt%? What is the actual composition of the samples? If Sb substituted Te site, where did the excess Te go?

Response: We sincerely appreciate your detailed attention to our compositional design. We are happy to clarify the rationale behind our notation, the actual phase composition, and the underlying defect chemistry. First, regarding the use of weight percentage (wt%): We chose this notation because our material is essentially a multiphase composite prepared through a metallurgical adding process, rather than a simple single phase solid solution. The system involves a base matrix of PbTe with specific mass additions of TeO_2 and Sb. Using weight percentage directly reflects the actual experimental weighing process for synthesis. It avoids the highly complex and impractical fractional subscripts that would be required if we used atomic percentages for a composite containing in situ generated oxides and varying defect concentrations. Second, regarding the substitution site of Sb: We would like to respectfully clarify a fundamental point in our defect design. In our n-type PbTe system, Sb atoms do not substitute the Te sites. Instead, Sb substitutes the Pb sites. Since Sb has three valence electrons and Pb has two, substituting Sb on the Pb site acts as an effective electron donor. Both our first principle calculations and experimental Hall effect data confirm that Sb replaces Pb, which provides the necessary electrons for n-type conduction and simultaneously induces the lattice softening discussed in the text. Third, regarding the actual composition and the fate of the excess Te: The actual composition of the sintered bulk is a composite consisting of a heavily Sb doped PbTe matrix, in situ generated Sb_6O_{13} nanoprecipitates, and a high concentration of specific point defects. During the displacement reaction ($13TeO_2 + 12Sb \rightarrow 2Sb_6O_{13} + 13Te$), elemental Te is indeed released. This generated Te plays two crucial roles. A portion of it compensates for the Te volatilization loss that inevitably occurs during the high temperature melting process. The remaining excess Te drives the matrix stoichiometry toward a Pb deficient state. In thermodynamics, an excess of anions Te in the lattice naturally promotes the formation of cation vacancies. This perfectly aligns with our discussion of Pb vacancies in the manuscript. Therefore, the excess Te is fully integrated into the defect chemistry equilibrium of the material without forming macroscopic secondary phases.

5. While repeatability within a batch appears good, what about reproducibility across different batches? Can similar performance be consistently reproduced in independently synthesized samples?

Response: We appreciate your constructive comment regarding the reproducibility of the thermoelectric performance. To address this concern and demonstrate the robustness of our experimental results, we would like to clarify that the three samples used for the repeatability tests (shown in **Supplementary Fig. 14**) were synthesized in a newly prepared, independent batch, which is entirely distinct from the batch used for the primary data presented in the main text. As shown in the comparison between the main text data and the supplementary data, the thermoelectric properties (including electrical conductivity, thermopower, and thermal conductivity) remain highly consistent across these different synthesis runs. This high degree of consistency between two independent batches provides strong evidence for the excellent batch-to-batch reproducibility of our PbTe-1.5wt.%TeO_2-1.8wt.%Sb composites and confirms that the reported high figure of merit *ZT*~2.2 is not an accidental result of a single synthesis cycle. We have added a clarifying statement in the revised Supplementary Information to explicitly specify the independent nature of these sample batches.

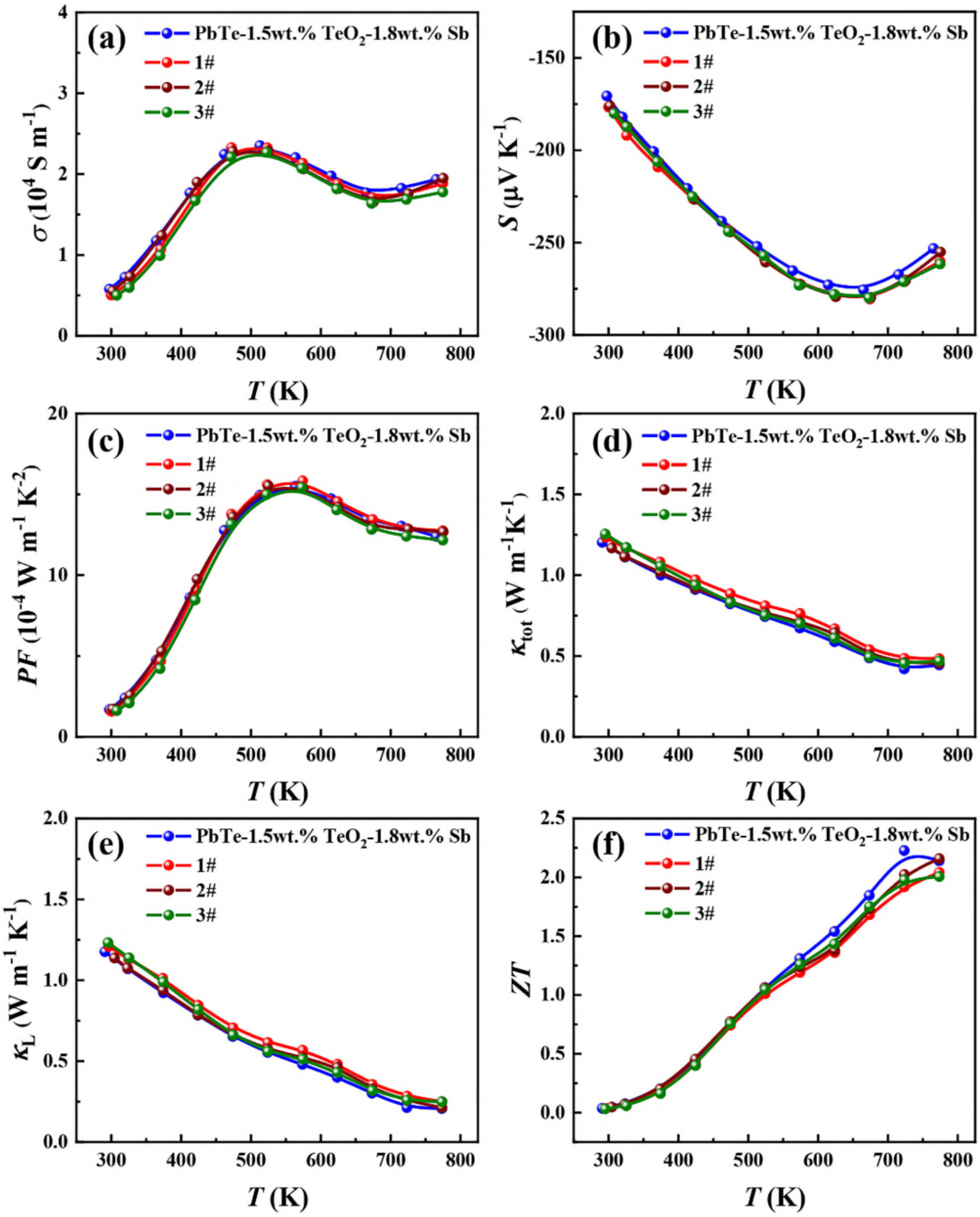

Supplementary Fig. 14 | Data repeatability of **(a)** electrical conductivity *σ*, **(b)** thermopower *S*, **(c)** power factor *PF*, **(d)** total thermal conductivity κ_{tot}, **(e)** lattice thermal conductivity κ_L and **(f)** *ZT* for PbTe-1.5wt.%TeO_2-1.8wt.%Sb composite. Note: The three samples for the repeatability test were from an independent synthesis batch, separate from the samples discussed in the main text, demonstrating excellent batch-to-batch reproducibility.

6. Nanostructures in thermoelectric materials often coarsen or disappear at high temperatures, leading to a loss of performance. In the present work, the authors suggest that this issue does not apply, but the reasoning behind this claim is not sufficiently explained. Could the authors clarify why the nanostructure is expected to remain stable under high temperature conditions in this system?

Response: We agree with you that the thermal stability of nanostructures is a critical factor for the practical application of thermoelectric materials. In our n-type PbTe system, the stability of the nanostructure and the resulting performance is ensured by the following three aspects:

(1) In-situ Formation and Endotaxial Interfaces: The Sb_6O_{13} nanoinclusions in our work are formed through an in-situ displacement reaction between Sb and TeO_2. Unlike externally added nanoparticles, these in-situ formed phases possess endotaxial or semi-coherent interfaces with the PbTe matrix. This leads to a lower interfacial energy, which significantly reduces the thermodynamic driving force for grain coarsening even at elevated temperatures.

(2) Strain-Induced Pinning Effect: The significant mismatch in thermal expansion coefficients between the Sb_6O_{13} nanoinclusions and the PbTe matrix generates persistent strain fields and a high density of dislocations. As captured by our GPA analysis **(Fig. R4)**, these intense strain lines and the complex dislocation networks act as "pinning centers" that mutually stabilize each other, effectively "freezing" the nanostructure at high temperatures.

(3) Experimental Validation via Independent Batches: The consistency observed across different synthesis batches (as shown in the repeatability tests in **Supplementary Fig. 14**) further validates that the formation of these stabilized nanostructures is a robust and intrinsic characteristic of our synthesis protocol, rather than a transient state.

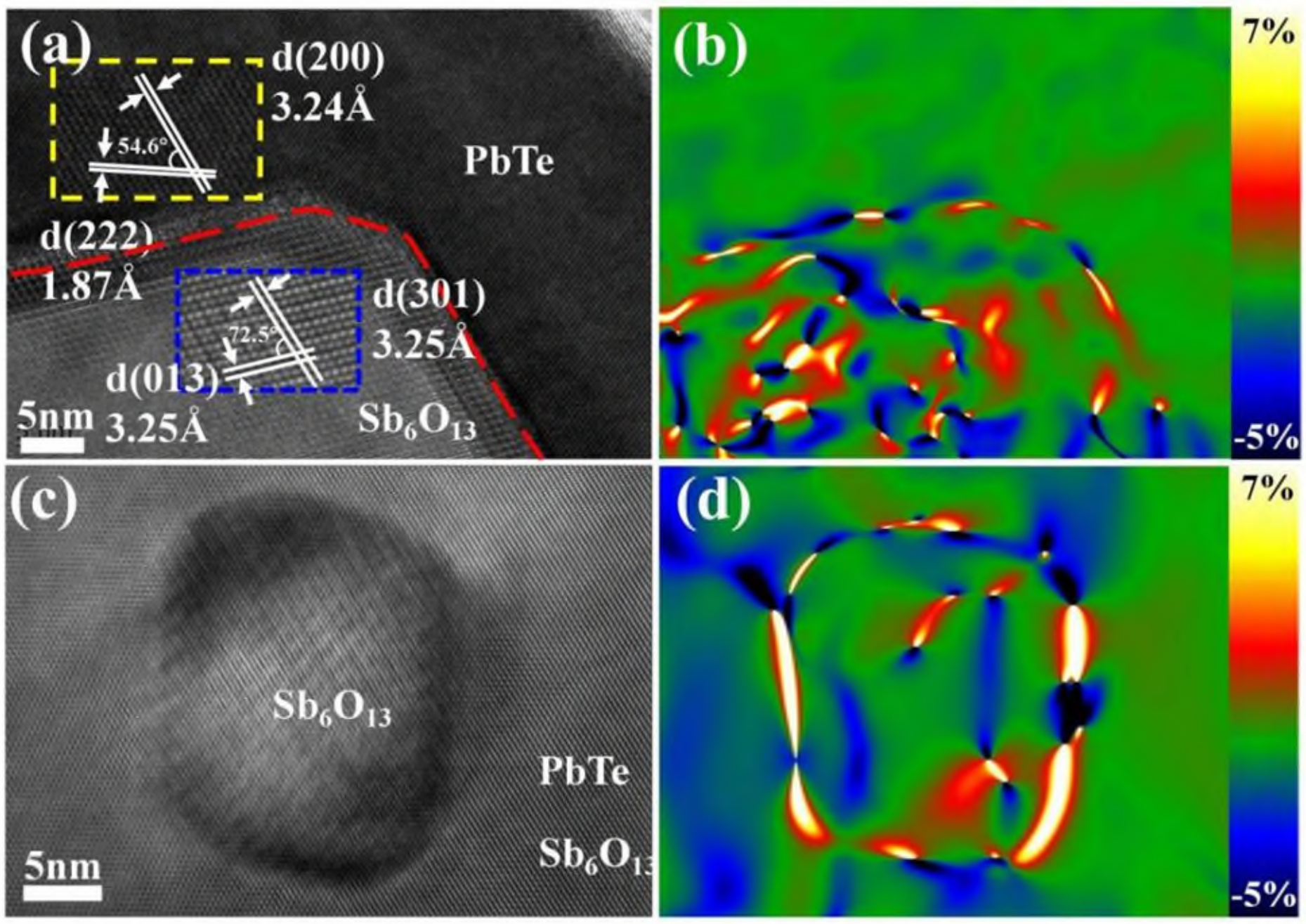

Fig. R4 | Microstructures of nanoprecipitate in PbTe-1.5wt.%TeO_2-1.8wt.%Sb sample. (a) high-magnification ABF image of Sb_6O_{13} nanoprecipitate; **(b)** GPA analysis of **(a)**; **(c)** high-magnification ABF image of Sb_6O_{13} nanoprecipitate; **(d)** GPA analysis of **(c)**.

7. The manuscript contains unnecessary adverbs such as “apparently,” “inevitably,” and “obviously.” These weaken the scientific tone and should be removed or replaced with more precise wording.

Response: Thank you for this constructive suggestion. We sincerely apologize for the inappropriate use of these adverbs. We agree with you that scientific claims should be supported by objective data rather than subjective emphasis. In the revised manuscript, we have conducted a thorough linguistic check and removed or replaced these adverbs with more precise, evidence-based descriptions.

Reply to reviewer 4

Comments: The authors report on the high thermoelectric performance of n-type PbTe-1.5wt.%TeO_2-1.8wt.%Sb material. A simulated conversion efficiency of 14% is calculated for three dimensional single-leg finite elements. The manuscript, together with the supplementary materials, provides a comprehensive and well-documented account of the experimental methods and analytical techniques employed to obtain the reported results and conclusions. The manuscript is well written and of great importance to the materials science community. I would recommend it to be published in Nature Communications after the following points are sufficiently addressed.

Thank you for your positive comments on our manuscript. Following is our response to your comments.

1. Figure S7(c) is presented to show the formation of relatively larger nanoinclusions in PbTe-1.5wt.%TeO_2-2.16wt.%Sb sample. However, it is not clearly shown. An EDS elemental mapping of Pb, Te, Sb, and O, similar to Figure 2 (b), can better support this argument.

Response: We appreciate your valuable suggestion to improve the clarity of our microstructural evidence. In accordance with your comment, we have performed EDS elemental mapping (Pb, Te, Sb, and O) on the PbTe-1.5wt.%TeO_2-2.16wt.%Sb sample to more clearly visualize the larger nanoinclusions. The new mapping results have been added as Supplementary Figs. 7c-d in the revised Supplementary Information, as shown in **Fig. R5**. These results clearly demonstrate the enrichment of Sb and O within the larger inclusions, confirming the in-situ formation of Sb_6O_{13} phases as discussed in the manuscript. We believe this additional characterization provides the necessary support for our argument regarding the morphology and composition of the secondary phases in this specific composition.

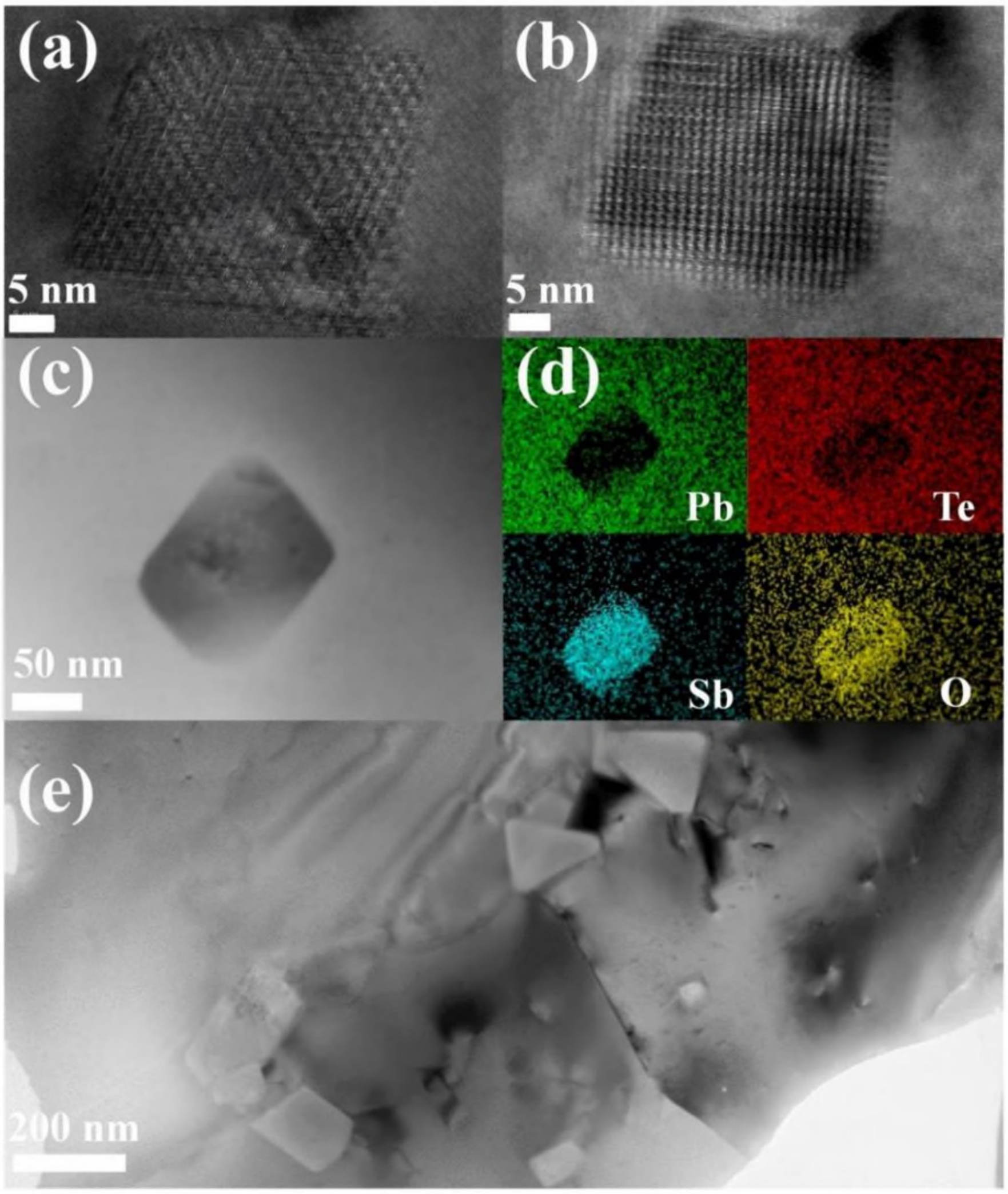

Fig. R5 | TEM micrographs of Sb_6O_{13} nanoinclusions in **(a-b)** PbTe-1.5wt.%TeO_2-1.8wt.%Sb sample and **(c-e)** PbTe-1.5wt.%TeO_2-2.16wt.%Sb sample.

2. Please ensure all acronyms are introduced with their full names upon first mention. For example, the acronyms for FFT and ABF are not addressed in the Supplementary Materials.

Response: Thank you for this helpful suggestion. We apologize for the oversight in the initial submission. In the revised Supplementary Information, we have ensured that all acronyms are defined upon their first mention. Specifically, FFT (Fast Fourier Transform) and ABF (Annular Bright-Field) have been addressed as requested. Furthermore, we have conducted a thorough check of both the main manuscript and the Supplementary Information to ensure that all other technical abbreviations are consistently and clearly introduced.

3. The authors cite several relevant studies throughout the manuscript; however, references are missing in places where they are particularly needed. For example, the second-to-last line of the second paragraph in the Introduction "which is well below the target of 2.2–2.8" requires an appropriate citation. A similar issue occurs in the third-to-last line of the fourth paragraph of the Introduction "which is about 10-fold larger than that of pristine PbTe", where a supporting reference should be provided.

Response: We thank you for this comment and apologize for the lack of clarity in our initial phrasing. The range of 2.2-2.8 refers to the state of the art *ZT* values achieved in p-type PbTe materials, which serves as the performance benchmark for our n-type counterpart. For a high-efficiency thermoelectric module, it is well-established that the performance of the p-type and n-type legs must be closely matched. While p-type PbTe has achieved record-high *ZT* values of ~2.2 to 2.8 (e.g., as reported in Ref. [1&13] cited in our manuscript), the *ZT* of n-type PbTe has historically lagged far behind. Therefore, the "target of 2.2-2.8" represents the performance level n-type materials need to reach to realize high-efficiency PbTe-based devices. In the revised manuscript, we have rephrased this sentence to explicitly state that this target is based on the benchmark performance of p-type PbTe and have added the supporting references as you suggested.

Besides, we would like to clarify that the "10-fold larger" comparison mentioned in the Introduction is made against the pristine PbTe sample synthesized and measured in our own experiments, rather than external literature values. The experimental data supporting this claim is quantitatively presented in **Fig. R6**, where the *ZT* values of our optimized samples are compared directly with those of our pristine PbTe baseline. To make this clearer for you and future readers, we have revised the text in the Introduction.

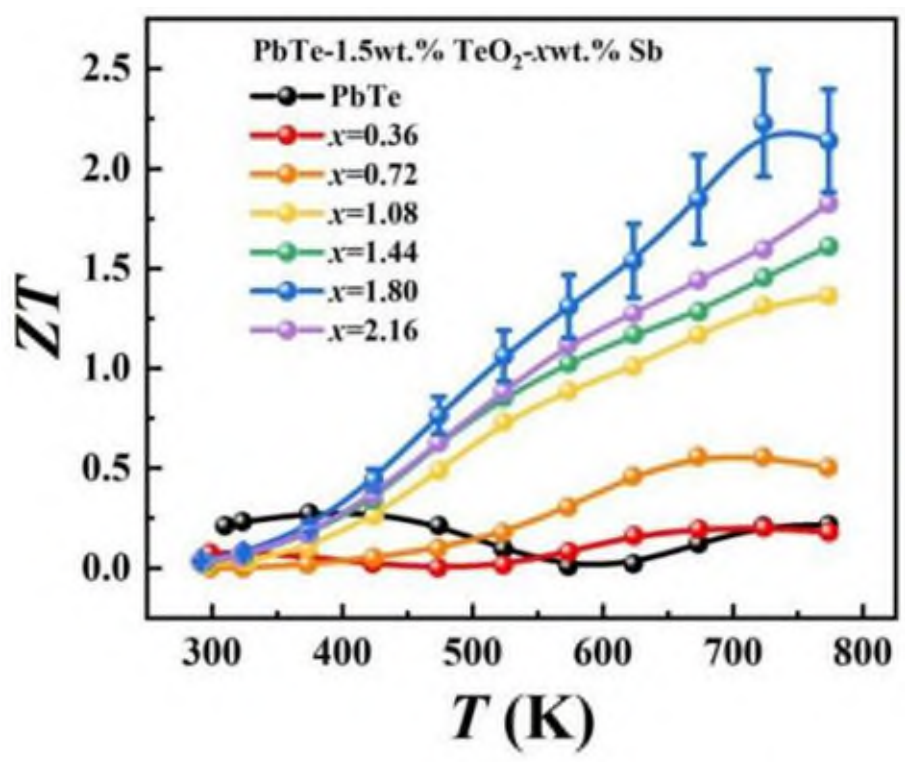

Fig. R6 | Temperature-dependent *ZT* of PbTe-1.5wt.%TeO_2-*x*wt.%Sb samples.

4. For the 4th paragraph of “Phase and microstructure characterization”: Providing a citation for “the theoretical value in PDF database” would be helpful, as it would allow readers to easily locate and verify the referenced data.

Response: Thank you for this constructive suggestion. We agree that providing specific PDF identifiers is essential for data traceability. In the revised manuscript (Section: Phase and microstructure characterization), we have included the PDF card numbers for both the primary PbTe phase (PDF No. 03-065-2953) and the secondary Sb_6O_{13} phase (PDF No. 01-071-1091). These citations will allow readers to easily locate and verify the referenced crystallographic data.

5. The claim of in-situ Sb_6O_{13} nanoinclusion formation would benefit from a clearer discussion of the conditions under which this secondary phase forms and whether its presence is consistently reproducible across independently prepared samples. Clarifying whether the repeatability tests were performed will further strengthen the manuscript.

Response: Thank you for this insightful comment. We agree that clarifying the formation mechanism and the reproducibility of the Sb_6O_{13} nanoinclusions is crucial for the impact of our work. Regarding the formation conditions, the Sb_6O_{13} phase is generated via an in-situ displacement reaction between Sb and TeO_2 during the high-temperature melting and subsequent sintering processes. Most importantly, to address your concern regarding reproducibility, we would like to clarify that the repeatability tests presented in the supplementary Information (**Supplementary Fig. 14**) were performed on samples from entirely independent synthesis batches. We have confirmed through both XRD and TEM characterizations that the Sb_6O_{13} nanoinclusions consistently form with similar morphology and distribution across these separate batches. This consistency in both microstructural evolution and the resulting thermoelectric performance demonstrates that the in-situ formation of Sb_6O_{13} is a highly reproducible process in our optimized material system. We have added a statement in the revised text to explicitly clarify that these repeatability tests involve independent batches.

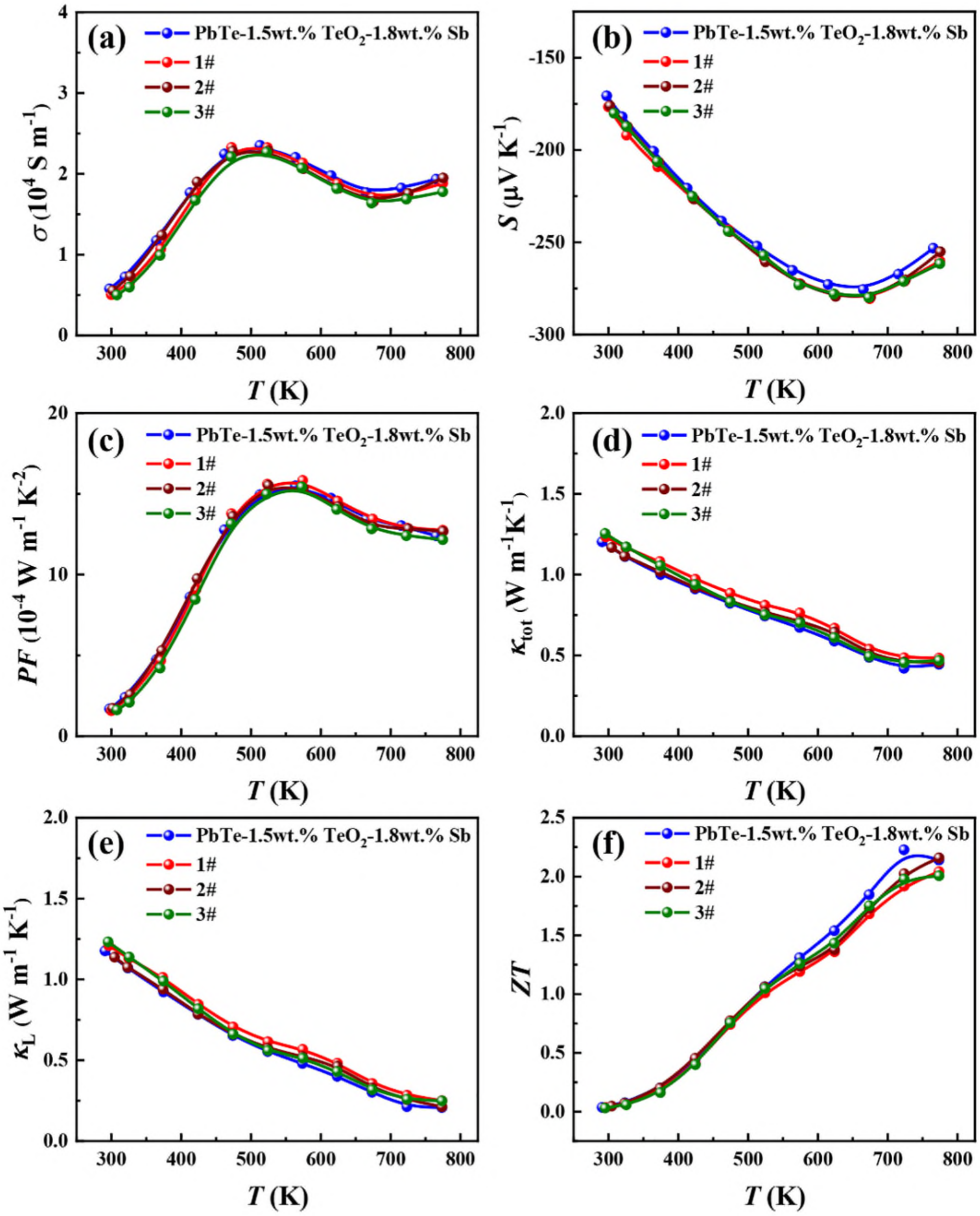

Supplementary Fig. 14. Data repeatability of **(a)** electrical conductivity σ, **(b)** thermopower S, **(c)** power factor PF, **(d)** total thermal conductivity κ_{tot}, **(e)** lattice thermal conductivity κ_L and **(f)** ZT for PbTe-1.5wt.%TeO_2-1.8wt.%Sb composite. Note: The three samples for the repeatability test were from an independent synthesis batch, separate from the samples discussed in the main text, demonstrating excellent batch-to-batch reproducibility.

Reply to reviewer 5

Comments: I co-reviewed this manuscript with one of the reviewers who provided the listed reports. This is part of the Nature Communications initiative to facilitate training in peer review and to provide appropriate recognition for Early Career Researchers who co-review manuscripts.

We sincerely appreciate the insightful and constructive comments provided by you and the co-reviewer. We also highly value the Nature Communications initiative to support the training of Early Career Researchers through the co-review process. The collaborative feedback has significantly helped us improve the quality and rigor of our manuscript.

Dear reviewers,

Thank you very much for your thorough review of our manuscript entitled "**Manipulating Peierls-Nabarro stress to realize extraordinary thermoelectric performance in n-type PbTe**" (Manuscript ID: NCOMMS-26-005960B). We sincerely appreciate the positive evaluation of our manuscript from reviewer 3 & reviewer 4, and value the insightful comments from reviewer 2. These comments are extremely valuable for improving the clarity, rigor, and overall quality of the manuscript.

A detailed, point-by-point response to all comments is provided below, and the manuscript has been revised accordingly. For details please see "The Revised Supplementary Information with Changes Marked", in which main revisions are highlighted in blue. We hope that this updated version addresses your concerns and is now suitable for publication in ***Nature Communications***.

Yours sincerely,

Prof. Dr. Jian Zhang.

On behalf of all co-authors

E-mail: zhangjian@issp.ac.cn

Reply to the reviewers' comments

Reply to reviewer 2

Comments: I appreciate the structural revisions you have made to the paper in response to my suggestions, and I now have a better understanding of your research strategy. However, I am not satisfied with your response to my previous request for the authors to demonstrate the density of dislocations in samples with different compositions. While the authors did present TEM photographs depending on the composition (Figure R3 in the response and Figure S5 in the supplementary information), they did not provide any numerical characterizations of dislocations or other defects. I estimate the dislocation density to be between 10^9 and 10^{11} cm^{-2} in the samples.

Reviewer 1 also noted the lack of quantitative data in the paper. The authors introduced pre-factors to simplify the parameters that characterize phonon scattering by various defects, except for nanoparticles, in order to avoid unreliable physical parameters from TEM images, such as dislocation density (N_D) and number density of nano-precipitates (N_P), as noted in the supplementary information. This results in an excellent evaluation, as shown in Fig. 3. At the same time, however, the scattering parameters, such as the scattering power, cross-section (or diameter) and mean-free path of individual defects, have been disregarded as if they were thrown into a black box. These parameters are the most important indexes from a physical viewpoint and for the future development of thermoelectric materials.

The authors replied that "SPS-processed bulk samples usually exhibit a non-equilibrium microstructure." (The acronym SPS requires the full notation.) Does this mean that the density of induced defects, including dislocations, depends on the sample preparation procedure, such as temperature, strain and stress? For instance, helical dislocations that are formed by absorbing point defects through screw dislocations are easily annihilated during annealing. From a reproducibility standpoint, as Reviewer 3 also noted, the reported results lack generality or universality for future research as defect densities depend heavily on the preparation procedure.

While this paper presents valuable results in the field of thermos-electrics through the manipulation of multidimensional defects and doping, I think that their research strategy would be more appropriate for a practice-oriented journal, unless the information on the physical characteristics of individual defects is provided.

Response: Thank you very much for your careful reading and thoughtful summary of our work. We sincerely appreciate your constructive and insightful comments, which help us further clarify the innovation and positioning of this work. With the response and supplements, we believe this work can better reveal the internal mechanism of performance improvement, and meet the publication standard of this journal. We sincerely hope that the revised manuscript can get your recognition.

(1) We completely agree with you that unlocking the "black box" by quantifying the physical characteristics of individual defects, such as the dislocation density (N_D), actual network spacing (d_{dis}), phonon mean free path (Λ_{dis}), and scattering cross-section (σ_{dis}), is paramount for a rigorous physical analysis. We sincerely apologize for omitting these explicit parameters in the previous version. In the revised manuscript and supplementary information, we have completely addressed this concern by systematically decoding these individual defect indexes. First, the experimental dislocation densities (N_D) were rigorously quantified from representative TEM micrographs (Supplementary Fig. 5) using the classic Ham's statistical linear-intercept method[1-3] (N_D = 2N/(L×t)). The results demonstrate a clear, monotonic increase from 1.14×10^{10} cm^{-2} (x = 0.36) to 3.89×10^{10} cm^{-2} (x = 1.08), and finally peaking at 6.99×10^{10} cm^{-2} (x = 1.80). This experimental gradient perfectly validates the physical baseline of the prefactors (A_3 and A_4) used in our Debye-Callaway model (Supplementary Table 3). To completely demystify the transport equations, we have derived the concrete physical characteristics of individual dislocation defects based on Klemens and Dexter-Seitz scattering formulations at a representative mid-to-high frequency acoustic phonon mode (ω≈1.5 THz, which corresponds to the dominant thermal phonon spectrum peak for PbTe at elevated temperatures):

Dislocation Network Spacing (d_{dis}): Structurally, the average physical distance between the entangled dislocation lines within the matrix lattice is geometrically defined by $d_{dis} = 1/\sqrt{N_D}$. For the optimal sample (x = 1.80), this geometric relation yields an intrinsic spatial mesh size of d_{dis} ≈37.8 nm. This derived spacing beautifully echoes the real-space dimensions of the dislocation cells and helical loops directly observed under our TEM characterizations (~40-60 nm), bridging the gap between transport equations and real microstructure.

Phonon Mean Free Path (Λ_{dis}): According to the Boltzmann transport relation, the dislocation induced phonon mean free path is given by $\Lambda_{dis}(\omega) = v_s \times \tau_{DS}(\omega)$, where τ_{DS} is evaluated from Supplementary formula (15) using our quantified experimental N_D. For the low-doping control sample

(x = 0.36), the calculated individual dislocation-induced phonon mean free path is as large as ~312 nm. For the peak-performance composite sample (x = 1.80), due to the intensive multiplication of dislocations, the phonon mean free path is rigorously suppressed down to $\Lambda_{dis} \approx 51$ nm.

Scattering Cross-Section (σ_{dis}): For line-like defects, the effective scattering cross-section (the effective scattering diameter per unit length) is defined by $\sigma_{dis}(\omega) = 1 / (N_D \times \Lambda_{dis}(\omega))$. Under the same frequency mode ($\omega \approx 1.5$ THz), the individual dislocation scattering cross-section is quantified to be $\sigma_{dis} \approx 28$ nm across the series.

We have summarized these parameters into the newly added Supplementary Table 4 and expanded the physical discussions in the Revised Supplementary Information.

Table R1. Detailed quantification of individual dislocation characteristics, including experimental density (N_D), spatial spacing (d_{dis}), phonon mean free path (Λ_{dis}), and scattering cross-section (σ_{dis}) evaluated at $\omega \approx 1.5$ THz.

Nominal composition	N_D(cm^{-2})	d_{dis} (nm)	Λ_{dis} (nm)	σ_{dis} (nm)
**PbTe-1.5wt.%TeO_2-0.36wt.%Sb**	1.14×10^{10}	~93.7	~312	~28
**PbTe-1.5wt.%TeO_2-1.08wt.%Sb**	3.89×10^{10}	~50.7	~91	~28
**PbTe-1.5wt.%TeO_2-1.80wt.%Sb**	6.99×10^{10}	~37.8	~51	~28

Since the fundamental physical constants (e.g., the Burgers vector and Grüneisen parameter) ruling the dislocation scattering cross-section (σ_{dis}) remain invariant within the fixed PbTe matrix, σ_{dis} manifests as an intrinsic characteristic scattering diameter of the material, evaluated at ~28 nm under the given phonon frequency. Remarkably, for the peak-performance sample (x = 1.80), the spatial spacing of the dislocation network (d_{dis}) is drastically compressed down to ~37 nm. This spatial dimension is exceptionally close to its intrinsic scattering cross-section of 28 nm, resulting in severe geometrically driven spatial overlapping of adjacent dislocation strain fields. This structural convergence provides a definitive physical elucidation for the dramatic suppression of the lattice thermal conductivity toward its amorphous limit.

(2) Thank you for your reminder, we have provided the full notation for spark plasma sintering (SPS) in the revised manuscript. We sincerely apologize for the less-than-accurate description in our previous

response regarding "SPS-processed bulk samples usually exhibit a non-equilibrium microstructure." To clarify, in Spark Plasma Sintering (SPS), a "non-equilibrium microstructure" does not imply chaotic or unrepeatable defect arrangements. Instead, it refers to a thermodynamically metastable state utilizing kinetic trapping: a widely adopted, cornerstone strategy to achieve state-of-the-art high performance (e.g., high-density dislocations induced by liquid-phase extraction or severe plastic deformation). The reproducibility of our synthesis route is exceptionally high because the generation and stabilization of these dislocations are governed by deterministic chemical and energetic driving forces, rather than random processing fluctuations:

Thermodynamic Driving Force (Solute-Induced Lattice Softening): As demonstrated by our DFT calculations, the incorporation of solute atoms alters the baseline elastic properties of the PbTe matrix, intrinsically lowering the Peierls-Nabarro stress and dislocation formation energy. Therefore, as long as the chemical composition (*x*) is precisely controlled, the thermodynamic driving force for yielding high-density dislocations remains identical across different batches.

Kinetic Stabilization (In-Situ Phase Pinning): You points out an important physical phenomenon that high-energy defect configurations, such as helical dislocations, are prone to annihilation during subsequent annealing. However, in our synthesized system, the concurrent in-situ displacement reaction between Sb and TeO_2 forms highly dispersed Sb_6O_{13} nanoinclusions. These rigid nanoinclusions act as powerful Zener pinning centers that strongly lock the dislocation lines, preventing them from climbing or slipping. Consequently, the induced helical dislocations and dislocation loops are mechanically and thermally anchored, rendering them highly resistant to annihilation during both the high-temperature spark plasma sintering process and subsequent transport measurements.

To demonstrate the rigorous reproducibility of this strategy, we have fabricated three independent batches of the optimal performance sample (x = 1.80) using the identical spark plasma sintering parameters . The resulting lattice thermal conductivity and overall figure of merit exhibit an extremely narrow margin of error (Supplementary Fig. 14). Therefore, our strategy offers strong universality and generality, providing a predictable and highly reproducible paradigm for defect engineering in advanced thermoelectrics.

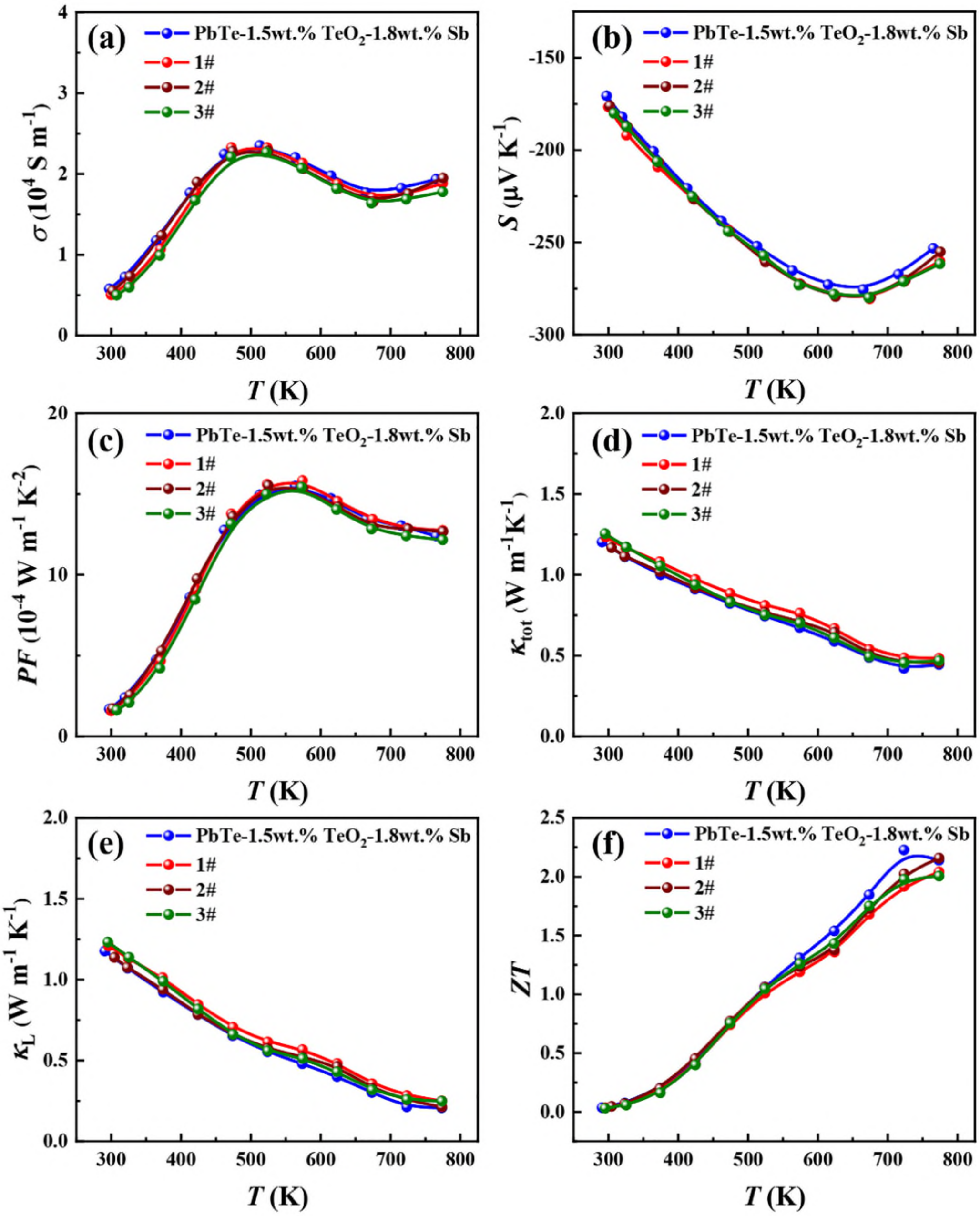

Supplementary Fig. 14 | Data repeatability of **(a)** electrical conductivity σ, **(b)** thermopower S, **(c)** power factor PF, **(d)** total thermal conductivity κ_{tot}, **(e)** lattice thermal conductivity κ_{L} and **(f)** ZT for PbTe-1.5wt.%TeO_2-1.8wt.%Sb composite. Note: The three samples for the repeatability test were from an independent synthesis batch, separate from the samples discussed in the main text, demonstrating excellent batch-to-batch reproducibility.

Reply to reviewer 3

Comments: In the revised manuscript their claims are understandable with additional information and explanation. The authors tried to understand the mechanisms by combining transport properties and microscopic analysis. The discovery does not have a huge impact on the field; however, the data itself is original and worth reporting. I agree to accept this as the publication.

Response: We are deeply encouraged by your final agreement to accept our manuscript for publication. Your constructive insights throughout the review process have been invaluable in enhancing the physical depth and clarity of our work. Thank you once again for your professional endorsement and strong support for publishing our work in *Nature Communications*.

Reply to reviewer 4

Comments: All my comments have been properly addressed. I believe this manuscript is now ready to be accepted for publishing.

Response: We are extremely grateful for your positive feedback and final endorsement for the publication of our manuscript. We sincerely thank you for your time, effort, and constructive comments throughout the peer-review process, which have greatly helped us improve the quality and presentation of our work. Thank you once again for your strong support for publishing our work in *Nature Communications*.

Reply to reviewer 5

Comments: I co-reviewed this manuscript with one of the reviewers who provided the listed reports. This is part of the Nature Communications initiative to facilitate training in peer review and to provide appropriate recognition for Early Career Researchers who co-review manuscripts.

Response: We sincerely thank you for participating in the co-review of our manuscript under this excellent *Nature Communications* initiative. We truly appreciate your time, effort, and dedicated support throughout the peer-review process to help improve the quality of our work.

References:

1. Ham, R. K. The determination of dislocation densities in thin films. *Philosophical Magazine* **6**, 1183-1184 (1961).
2. Agudo Jácome, L. *et al.* High-temperature and low-stress creep anisotropy of single-crystal superalloys. *Acta Materialia* **61**, 2926-2943 (2013).
3. Wang, X. *et al.* High entropy oxide (Co,Cu,Mg,Ni,Zn)O exhibits grain size dependent room temperature deformation. *Materials Research Letters* **11**, 196-204 (2022).

Dear reviewer,

Thank you for your thorough review and positive evaluation of our manuscript entitled "**Manipulating Peierls-Nabarro stress to realize extraordinary thermoelectric performance in n-type PbTe**" (Manuscript ID: NCOMMS-26-005960C). We have carefully addressed your insightful comments, which have significantly improved the clarity and rigor of our work.

A detailed, point-by-point response to all comments is provided below, and the manuscript has been revised accordingly. For details please see "The Revised Manuscript with Changes Marked", in which main revisions are highlighted in blue. We hope that this updated version addresses your concerns and is now suitable for publication in ***Nature Communications***.

Yours sincerely,

Prof. Dr. Jian Zhang.
On behalf of all co-authors
E-mail: zhangjian@issp.ac.cn

Reply to the reviewers' comments

Reply to reviewer 2

Comments: I have reviewed the revised manuscript and supplementary materials, as well as the authors' responses to the previous comments regarding the physical characteristics of individual defects. The authors responded appropriately to my questions and provided the necessary information, primarily in the supplementary materials. I am currently inclined to give the paper a positive review, provided that the dislocation densities are described explicitly in the manuscript, possibly around Figs. 1 or 2, rather than in the supplementary materials. The numerical magnitudes are key information in the present research, given that the authors claim success in manipulating P-N stress to introduce an extremely high density of dislocations by the severe deformation. The lack of notation for the density is rather strange.

The following is an optional extra, not necessary to act. I am interested in whether the thermoelectric properties of such a high-energy or high-entropy state can be maintained on the TE timescale under practical heat conditions.

Response: We sincerely thank you for the positive assessment and constructive suggestions. Regarding your specific points:

(1) We completely agree with your insightful judgment that quantified dislocation densities form the foundational evidence validating our Peierls-Nabarro (P-N) stress engineering mechanism. Following your suggestion, we have incorporated the content from Supplementary Information into the main text, placing it immediately after the microstructural characterizations shown in Figure 1. The exact paragraph to be inserted into the revised manuscript is provided below, while the full quantitative calculation workflow via Ham's linear-intercept method remains archived in Supplementary Section 6 and Supplementary Table 4 to retain complete methodological transparency for readers.

Main-text insertion paragraph (paste after Figure 1 TEM description in revised manuscript):

Besides, quantitative statistical analysis of multiple TEM micrographs via Ham's linear-intercept method reveals a pronounced elevation of dislocation density (N_D) with increasing Sb solute content. For PbTe-1.5 wt.% TeO_2-0.36 wt.% Sb, N_D reaches 1.14×10^{10} cm^{-2}; this value rises to 3.89×10^{10} cm^{-2} for PbTe-1.5 wt.% TeO_2-1.08 wt.% Sb, and peaks at an ultrahigh magnitude of 6.99×10^{10} cm^{-2} in the optimal PbTe-1.5 wt.% TeO_2-1.8 wt.% Sb composite. In this record-performance sample, the average

spatial separation of entangled dislocation networks is reduced to ~37.8 nm, a length scale comparable to the intrinsic phonon scattering cross-section of individual dislocations (~28 nm). Such dense dislocation populations originate from Sb-induced lattice softening and suppressed P-N stress, which drastically lower the energetic barrier for dislocation multiplication during melt quenching and spark plasma sintering. The overlapping strain fields emanating from interconnected dislocation lines deliver robust broadband scattering for mid- and low-frequency heat-carrying phonons, and play a significant role in suppressing lattice thermal conductivity.

(2) We sincerely thank you for raising this practically oriented question. We completely agree that maintaining thermoelectric properties under actual heat conditions is crucial for this high-strain state. In fact, to address this practical concern, we had already conducted comprehensive thermal stability and reproducibility evaluations, which are detailed in our Supplementary Information:

First, robust thermal cycling stability (Supplementary Fig. 15): We tested the optimal PbTe-1.5 wt.% TeO_2-1.8 wt.% Sb sample over a full heating–cooling–reheating cycle (~13h) covering the entire working temperature range of 300-773 K. As demonstrated in Supplementary Fig. 15, no obvious changes were detected in electrical conductivity, thermopower, total thermal conductivity, or the peak *ZT* value after repeated high-temperature exposure.

Second, intrinsic defect pinning mechanisms: Two mechanisms stabilize our architecture against high-temperature defect recovery. The in-situ formed Sb_6O_{13} nanoprecipitates develop coherent/semi-coherent interfaces with the PbTe matrix along {111} habit planes, acting as immobile pinning barriers that block dislocation glide, climb, and permanent annihilation. Moreover, the homogeneous lattice strain fields introduced by aliovalent Sb^{3+} substitution for Pb^{2+} further restrict dislocation mobility. Since the maximum service temperature (773 K) is markedly lower than the recrystallization threshold (823 K) of spark-plasma-sintered PbTe, large-scale dislocation recovery is thermodynamically unfavorable.

Third, batch-to-batch reproducibility (Supplementary Fig. 14): As presented in Supplementary Fig. 14, independent synthesis batches confirm that the ultrahigh dislocation density and corresponding thermoelectric transport properties are robustly reproducible. This rules out the possibility that the high-strain microstructure arises from random transient deformation.

Collectively, the persistent pinning effect of Sb_6O_{13} nanoprecipitates is proven to sustain efficient phonon scattering over typical thermoelectric operating durations, guaranteeing excellent stability for

practical applications.

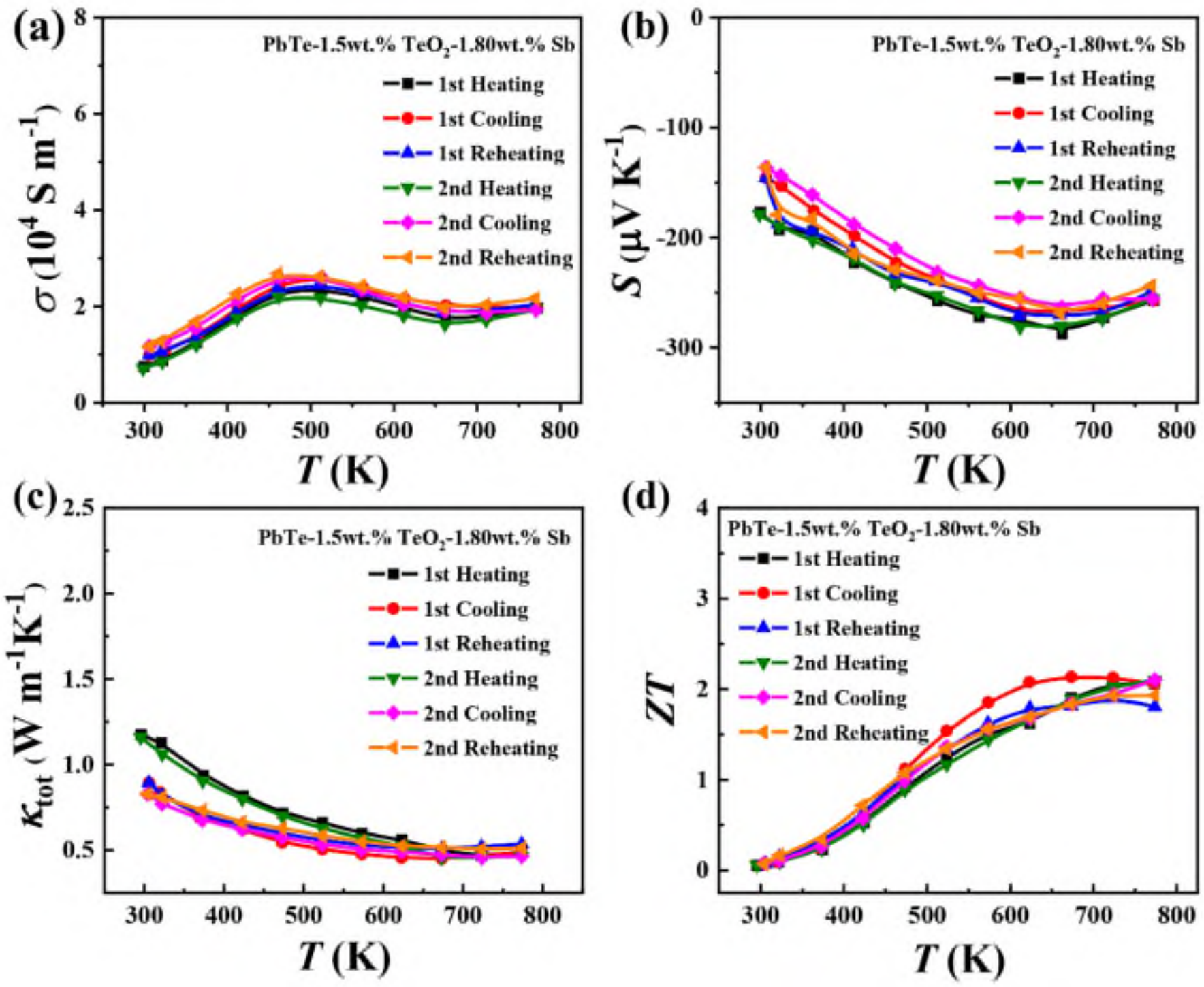

Supplementary Fig. 15 | Thermal stability of **(a)** electrical conductivity σ, **(b)** thermopower S, **(c)** total thermal conductivity κ_{tot}, and **(d)** ZT for PbTe-1.5wt.%TeO_2-1.8wt.%Sb composite.

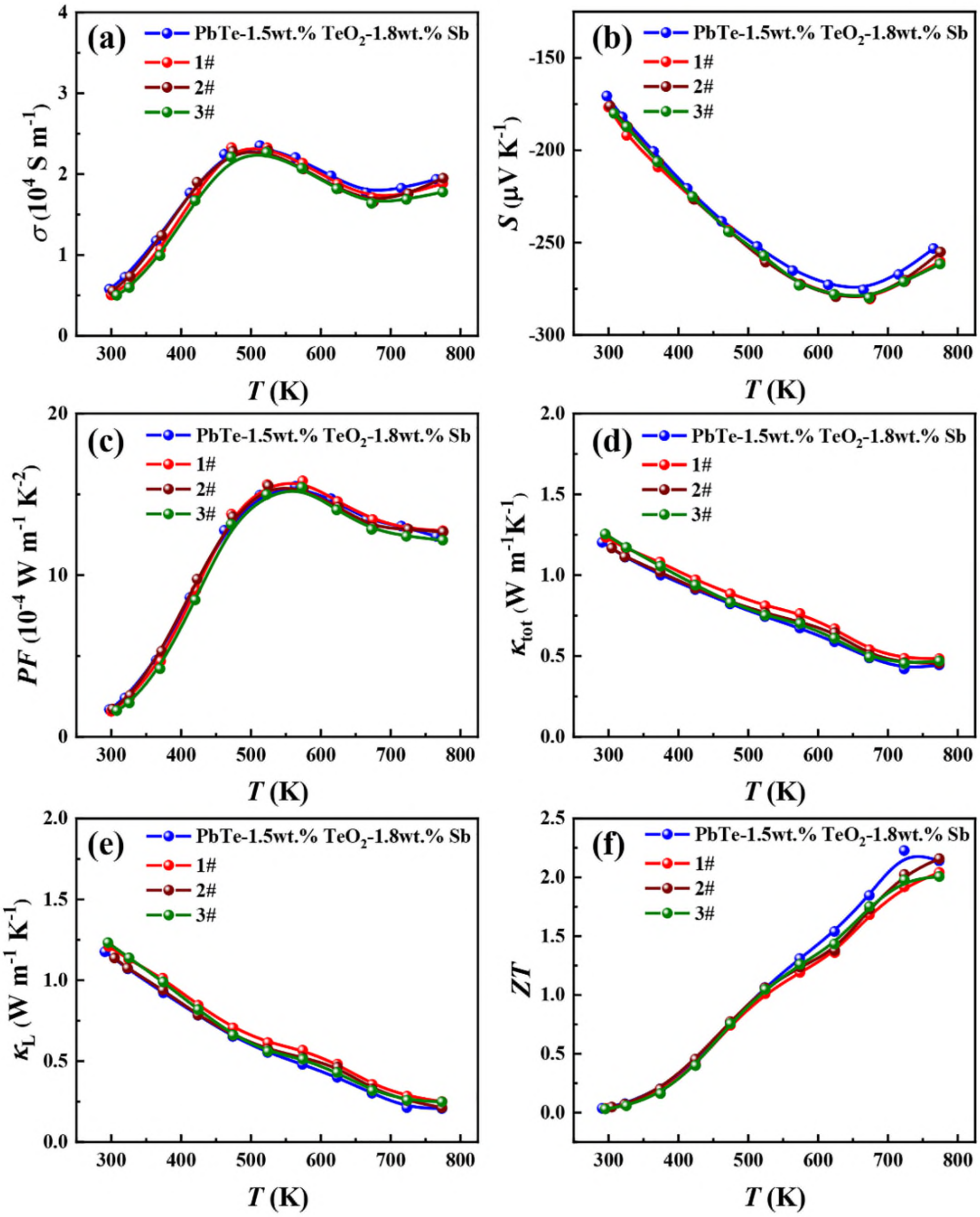

Supplementary Fig. 14 | Data repeatability of **(a)** electrical conductivity σ, **(b)** thermopower S, **(c)** power factor PF, **(d)** total thermal conductivity κ_{tot}, **(e)** lattice thermal conductivity κ_L and **(f)** ZT for PbTe-1.5wt.%TeO_2-1.8wt.%Sb composite. Note: The three samples for the repeatability test were from an independent synthesis batch, separate from the samples discussed in the main text, demonstrating excellent batch-to-batch reproducibility.

Dear reviewer,

Thank you for your final positive evaluation of our manuscript entitled "**Manipulating Peierls-Nabarro stress to realize extraordinary thermoelectric performance in n-type PbTe**" (Manuscript ID: NCOMMS-26-005960D). We are delighted to learn that our previous revisions have fully addressed your concerns, and we sincerely appreciate your support for the publication of our work.

We have included your final remarks and our corresponding acknowledgment below. We hope the finalized manuscript is now fully ready for publication in ***Nature Communications***.

Yours sincerely,

Prof. Dr. Jian Zhang

On behalf of all co-authors

E-mail: zhangjian@issp.ac.cn

Reply to the reviewers' comments

Reply to reviewer 2

Comments:

I am satisfied with your responses and the revised manuscript, which perfectly addressed my comments. I am now happy to support the publication of your paper in this journal. I appreciate your thoughtful responses to my comments and wish your further success in exploring materials in various fields including TE.

Response: We sincerely thank you for your positive feedback and your strong support for the publication of our manuscript. We are delighted that our revisions and previous responses have perfectly addressed your concerns. We would also like to express our deepest gratitude for the time and effort you have dedicated to reviewing our work, as well as for your constructive suggestions throughout the review process, which have significantly improved the quality of our paper. Finally, we truly appreciate your kind wishes for our future research endeavors.
